# Ancient genomes from eastern Kazakhstan reveal dynamic genetic legacy of Inner Eurasian hunter-gatherers

**DOI:** 10.1126/sciadv.adw8219

**Published:** 2025-10-15

**Authors:** Haechan Gill, Madina Seidualy, Juhyeon Lee, Jiyoung Lee, Hyungmin Moon, Antonia Walter, Raffaela Angelina Bianco, Arman Kurmangaliev, Erbolat Rakhmankulov, Zainolla Samashev, Azat Aitkali, Galymzhan Kiyasbek, Zhuldyz Tashmanbetova, Aidyn Zhuniskhanov, Johannes Krause, Taylor Hermes, Maxat Zhabagin, Paula Doumani Dupuy, Christina Warinner, Choongwon Jeong

**Affiliations:** ^1^ School of Biological Sciences, Seoul National University, Seoul, 08826, Republic of Korea.; ^2^ Institute for Data Innovation in Science, Seoul National University, Seoul, 08826, Republic of Korea.; ^3^ National Center for Biotechnology, Astana, 010000, Kazakhstan.; ^4^ School of Sciences and Humanities, Nazarbayev University, Astana, 010000, Kazakhstan.; ^5^ Department of Anthropology, University of Texas at Austin, Austin, TX 78712, USA.; ^6^ Max Planck Institute for Evolutionary Anthropology, Leipzig, 04103, Germany.; ^7^ National Museum of the Republic of Kazakhstan, Astana, 010000, Kazakhstan.; ^8^ State Historical and Cultural Museum-Reserve “Bozok,” Astana, 010000, Kazakhstan.; ^9^ Margulan Institute of Archaeology, Almaty, 050000, Kazakhstan.; ^10^ Department of Anthropology, Washington University in St. Louis, St. Louis, MO 63130, USA.; ^11^ Department of Anthropology, University of Arkansas, Fayetteville, AR 72701, USA.; ^12^ Department of Anthropology, Harvard University, Cambridge, MA 02138, USA.

## Abstract

Because of limited availability of ancient genomes, the genetic history of prehistoric Inner Asian hunter-gatherers remains incomplete, especially for eastern Kazakhstan where the Eurasian Steppe meets mountain forests of Inner Asia. Here we report genome-wide data of two Early Neolithic (EN) hunter-gatherers and 19 Middle-Late Bronze Age (MLBA) pastoralists, from the site of Koken in the Upper Irtysh River region in eastern Kazakhstan. We find that the two EN individuals differed in their genetic profiles and yet were second-degree relatives. They were genetically most similar to subsequent Neolithic individuals in the Irtysh region, while contemporaneous hunter-gatherers from the Tobol-Ishim and Upper Ob River regions had distinct genetic profiles, likely influenced by riverine geography. The Koken MLBA individuals were genetically similar to other MLBA steppe pastoralists, while genetic outliers provide evidence of two distinct trajectories of admixture with local hunter-gatherer populations. These findings illuminate the dynamic population structure of Inner Asian hunter-gatherers and their genetic legacy in subsequent pastoralist populations.

## Teaser

Inner Asian hunter-gatherers were structured by riverine geography and contributed genetically to later pastoralists.

## INTRODUCTION

The populations of Inner Asia are remarkably diverse yet structured into at least three distinct major admixture clines reflecting geography and ecozones (*1*). Recent archaeogenomic studies have reconstructed many of the major events and demographic processes that contributed to the present-day genetic diversity of this region (*2*–*7*). However, while the genetic history of Inner Asia from the Bronze Age onward is now coming into focus, the region’s hunter-gatherer past and its genetic legacy among later populations are much less understood. Broad attempts to map the genetic diversity of hunter-gatherer populations before the rise of pastoralism as the major economic lifeway on the Eurasian steppes and adjacent forest zones have identified multiple distinct hunter-gatherer ancestries, revealing a glimpse into a complex history of hunter-gatherer mobility and genetic mixing since the end of the Pleistocene (*4*, *8*–*13*). However, poor resolution of local hunter-gatherer diversity has challenged population modeling of subsequent admixed populations, such as the Early Bronze Age (EBA) Khemtseg (Chemurchek) of the Altai-Sayan region and Dzungarian Basin, leading to conflicting models of their genetic histories (*4*, *7*, *12*). A high-resolution reconstruction of the genetic landscape of Inner Asian hunter-gatherers is therefore critical for understanding the region’s population history and long-term genetic legacy throughout Eurasia and the Americas.

The mortuary record of Inner Asia is well-attested from the Bronze Age onward, in large part due to pastoralist traditions that mark inhumations with large earthen funerary mounds or stone architecture (*14*, *15*). Such conspicuous burials, which are easily visible both by remote sensing and pedestrian surveys, have attracted strong archaeological interest and consequently represent the majority of the documented archaeological record of prehistoric Inner Asia (*16*). Earlier hunter-gatherer burials, in contrast, are far less archaeologically visible and represent only a small fraction of the human remains available for paleogenomic study. Currently known hunter-gatherer burial sites are sparsely distributed throughout the region, often with large temporal and geographic gaps between them. Those that are available are mostly concentrated in the Altai-Sayan region of southern Russia and areas surrounding Lake Baikal (*9*–*11*, *13*, *17*) and Mongolia (*18*), leaving most of the Eurasian steppe zone and Inner Asian Mountain Corridor (IAMC) represented by only a handful of hunter-gatherer genomes (*4*, *9*). As the Eurasian steppe and IAMC were the two main geographic routes for the spread of pastoralism into Asia during the Bronze Age (*19*–*22*), the dearth of local hunter-gatherer genomes from these regions makes it difficult to reconstruct the social and demographic processes that led to the rise of Inner Asian pastoralist societies and the later development of nomadic empires.

**Fig. 1.**
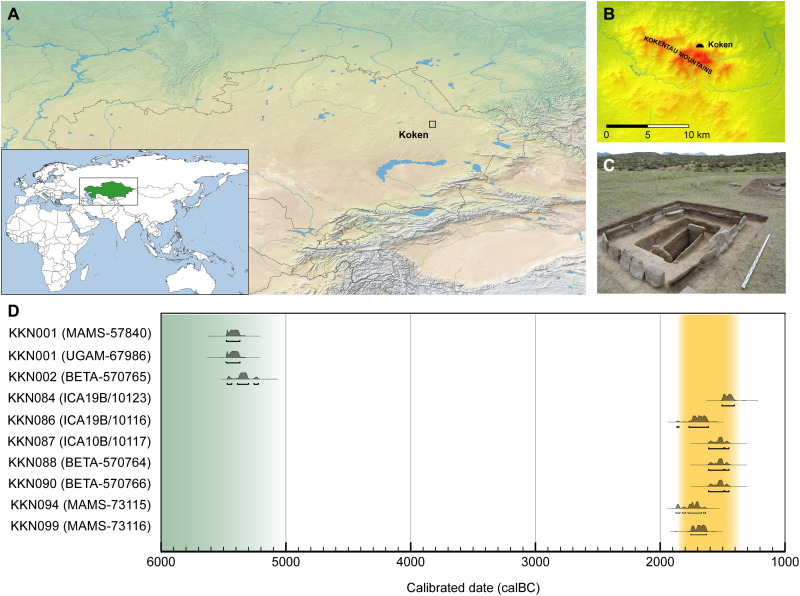
Archaeological site of Koken. (**A**) Koken is a multiperiod site occupied since the Epipaleolithic in Abai region of eastern Kazakhstan (inset, green). The basemaps used in (A) are in the public domain and accessible through the Natural Earth website (https://naturalearthdata.com/downloads/10m-raster-data/; last accessed 3 March 2022). (**B**) Koken is situated in a semiarid steppe zone along the northern foothills of the Kokentau Mountains, and it falls within a biodiverse ecosystem of steppe grasslands, seasonal streams, birch and juniper groves, marshland, and mineral outcrops. (**C**) Excavation photograph of Burial 13 (KKBR13), a representative MLBA Andronovo culture cist grave surrounded by a stone fence containing the remains of individual KKN090. (**D**) Calibrated radiocarbon dates for individuals at Koken, showing 95.4% probability. The burials date broadly within the EN (green) and MLBA (yellow) periods. Dates were calibrated with OxCal v4.4.4 (*99*) using atmospheric data from Reimer *et al.* (*55*) (Photo Credit: P.D.D., Nazarbayev University).

Here, we report new genomic data from 21 individuals at the site of Koken (Fig. 1A), a multiperiod and multicomponent site with both hunter-gatherer and pastoralist occupations located in the Upper Irtysh River region of eastern Kazakhstan in the semiarid grasslands of the Eurasian steppe, bordering the foothills of the Altai Mountains at the northeastern extent of the IAMC (*23*, *24*). Intermittently inhabited since the Epipaleolithic (ca. 12,000 BCE), the site contains a settlement and nearby graveyards together consisting of more than 70 Bronze Age burials (ca. 1870 to 1400 BCE) with a mortuary pattern and artifact inventory consistent with a widespread Middle-Late Bronze Age (MLBA) archaeological horizon of pastoralists stretching from the Caspian Sea in the west to as far east as the Altai-Sayan region (*23*). Largely subsumed within the Andronovo family of cultures, the burial grounds are well documented archaeologically, but little is known about their internal organization with respect to familial or kinship relationships, or how earlier hunter-gatherer legacies (genetic and/or cultural) may be reflected in MLBA mortuary contexts. For earlier periods, such as the Neolithic, the ability to trace histories of settlement and hunter-gatherer interactions in riverine environments of Inner Asia is hampered by a lack of stratified Neolithic sites and human burials, especially in Kazakhstan. Recently, the remains of two individuals (fig. S1) were discovered by chance at Koken, beneath the architectural remains of a Bronze Age settlement (*24*). Directly dated to the sixth millennium BCE (table S1) during the Early Neolithic (EN), the EN individuals reported here from Koken are the two oldest human remains yet discovered in Kazakhstan, and they present a rare opportunity to explore the region’s hunter-gatherer genomic past in combination with cultural expressions of kinship and mortuary practices.

Overall, we find that Koken hunter-gatherers have a genetic profile that is distinct from other hunter-gatherer populations in the Tobol-Ishim River region to the west and Upper Ob River region to the east, hinting at a high degree of local population structure among Inner Asian hunter-gatherer groups. Notably, the two Koken hunter-gatherers themselves showed distinct ancestry profiles but were also second-degree relatives, suggesting connectivity and interaction between local hunter-gatherer populations. Subsequent MLBA individuals buried at Koken (fig. S2) showed both cultural (figs. S3 to S6) and genetic affinity to regional pastoralist groups, but genetic outliers among these individuals hint toward at least two different trajectories of admixture with prior local populations in the formation of Bronze Age pastoralist communities in eastern Kazakhstan.

## RESULTS

### The genetic profile of ancient Koken individuals

**Fig. 2.**
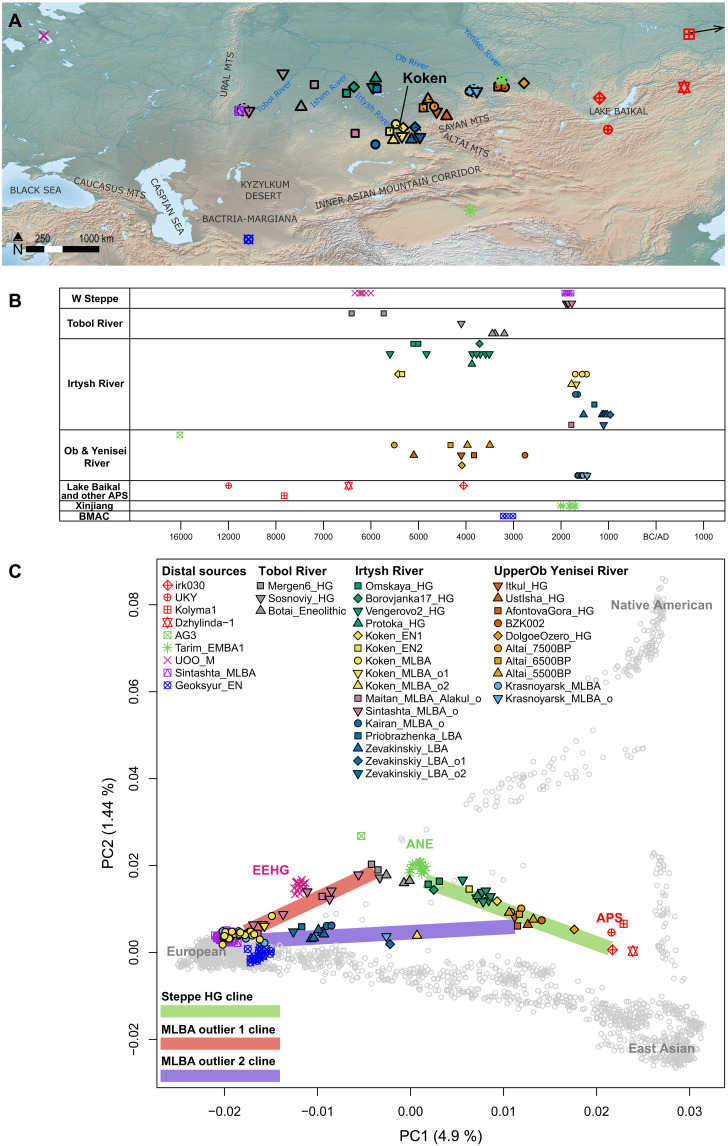
Archaeological information and genetic profiles of Siberian forest-steppe hunter-gatherers analyzed in this study. (**A**) Map showing locations of key ancient individuals whose genomes were analyzed in this study. The individual Kolyma_M lies beyond the geographic scope of the map; its approximate location is indicated by an arrow. The basemap used in (A) is in the public domain and accessible through the Natural Earth website (https://naturalearthdata.com/downloads/10m-raster-data/; last accessed 3 March 2022). (**B**) Radiocarbon dates of Koken and other ancient individuals analyzed in this study. BMAC, Bactria-Margiana Archaeological Complex. (**C**) Top two PCs were calculated from 2270 present-day Eurasian and American individuals. Gray circles mark present-day individuals used for calculating PCs. Ancient individuals, marked by color-filled symbols, were projected onto the calculated PCs. The green line marks the genetic admixture cline of prepastoralist steppe hunter-gatherers, stretching between the ANE and APS populations. The red and purple lines mark the distribution of MLBA genetic outliers, each suggesting a mixture with a distinct local population who resemble Tobol_HG (e.g., Mergen6_HG) and Irtysh_HG_2 (e.g., BZK002), respectively. Color-filled symbols used in (A) and (B) are listed in the legend in (C).

We generated genome-wide data for 21 ancient individuals from the Koken archaeological site (Fig. 2 and data S1), consisting of 2 EN individuals (5477 to 5222 cal. BCE) and 19 MLBA individuals (1875 to 1407 cal. BCE) (Fig. 1B and table S1). The ancient Koken individuals cover 33,767 to 1,231,988 (median 328,206) single-nucleotide polymorphisms (SNPs) of 1,233,013 ancestry informative SNPs in an in-solution enrichment panel (“1240K” panel) (*17*, *25*) and show negligible levels of human DNA contamination: ≤4% mitochondrial contamination for all 21 individuals and ≤4% X chromosome–based nuclear contamination for 8 males. For one particularly well-preserved EN individual (KKN001; 75.6% endogenous DNA), we additionally produced a high-coverage (29.8×) whole genome and made diploid genotype calls across the genome.

To compare the genetic profiles of Koken with other ancient individuals, we conducted principal components analysis (PCA) (*26*) of 2270 present-day Eurasian and American individuals and projected Koken and previously published ancient individuals onto the top principal components (PCs) (Fig. 2 and data S2 and S3). PC1 (*x* axis) distinguishes eastern and western Eurasians, while PC2 (*y* axis) separates Native Americans from Eurasians. As expected, we observe that the EN and MLBA Koken individuals have clearly distinguishable genetic profiles. The two EN individuals fall on a genetic cline of pre-Bronze Age Siberian hunter-gatherers connecting Ancient North Eurasians (ANE), represented by the 4000-year-old Bronze Age Tarim Basin individuals from the Xiaohe and Gumugou archaeological sites (Tarim_EMBA1) (*12*), and Ancient Paleo-Siberians (APS), including the following four published ancient individuals: the 14,000-year-old individual from the Ust-Kyakhta-3 archaeological site near the Lake Baikal (UKY) (*11*), 9800-year-old individual from the Duvanny Yar site in northern Siberia (“Kolyma_M”) (*27*), 8500-year-old individual from the East Baikal (“Dzhylinda-1”), and the 6100-year-old Late Neolithic West Baikal individual (“irk030”) (*28*). In contrast, most MLBA Koken individuals form a cluster overlapping with Central Asian MLBA individuals recovered from archaeological contexts linked to the Fedorovka (Andronovo) Culture Horizon (e.g., Krasnoyarsk_MLBA) (*29*). Compared to the EN individuals, they show overall much higher genetic affinity with ancient and present-day western Eurasian populations. Notably, two Koken_MLBA outliers were observed on the PCA plot. One was slightly displaced from the main cluster toward ANE populations (Koken_MLBA_o1; KKN094), while the other shifted to the right along PC1 from the main Koken_MLBA cluster (Koken_MLBA_o2; KKN099). In combination with their relatively early dates among the Koken_MLBA individuals (table S1), these outlier individuals potentially reflect a history of admixture between local hunter-gatherers and incoming Western Eurasian herders.

### Genetic ancestry of the EN Koken individuals

We find that the two EN Koken individuals were second-degree relatives, likely either an uncle-nephew or half-brother relationship (but unlikely a grandfather-grandson pair or a double cousin), based on their pairwise genotype mismatch rate (PMR), sharing of the Y haplogroup N, different mitochondrial haplogroups, and their sharing pattern of identity-by-descent (IBD) segments (fig. S7 and data S1 and S4) (*30*, *31*). We excluded a grandfather-grandson pair due to a mismatch in the IBD segment distribution and a double first cousin relationship due to lack of IBD2 segments and different mitochondrial haplogroups. They also showed no evidence of consanguineous pairing in their recent ancestors (fig. S8). Despite their close familial relationship, the two EN Koken individuals fall in distinct positions in PC space (Fig. 2), and thus, we first analyzed them individually (referred to as Koken_EN1 and Koken_EN2 in this study).

Outgroup f3 statistics (*10*, *32*) revealed close genetic affinities between the EN Koken individuals and the previously reported ancient populations along the ANE-APS cline, such as Tarim_EMBA1 and Altai hunter-gatherers (Altai_HGs) (fig. S9 and data S5) (*13*). As expected from their distinct location in PC space, we confirm that Koken_EN1 and Koken_EN2 are differently related to other ancient populations along the ANE-APS cline, as measured by the f4 symmetry test (*32*) of the form f4(Mbuti, world-wide; Koken_EN1, Koken_EN2) (fig. S10 and data S6). Overall, Koken_EN1 shows excess genetic affinity with East Asians [e.g., f4(Mbuti, DevilsCave_N; Koken_EN1, Koken_EN2) = −3.08 SEM], while Koken_EN2 is closer to those with the ANE ancestry [e.g., f4(Mbuti, Tarim_EMBA1; Koken_EN1, Koken_EN2) = 3.19 SEM]. Likewise, Koken_EN1 and Koken_EN2 fail a cladality test by qpWave (*33*) (*P* = 8.29 × 10^−4^; data S7). Considering their second-degree relatedness, it implies a familial relationship between individuals of distinct genetic profiles.

Reflecting their close kinship but distinct genetic profiles, we find that Koken_EN1 and Koken_EN2 provide a suitable proxy for the major source of each other’s admixture modeling using qpAdm (*3*). Koken_EN1 is adequately represented by an admixture between Koken_EN2 and an APS individual irk030 (*P* = 0.847, 75.7% contribution from Koken_EN2). Reciprocally, Koken_EN2 is similarly represented by a mixture of Koken_EN1 and Sosnoviy_HG, a 6000-year-old hunter-gatherer individual from the Sosnoviy island site near Novosibirsk (*P* = 0.938, 78.0% contribution from Koken_EN1) (data S7).

### Population dynamics of Siberian forest-steppe hunter-gatherers

To understand the genetic history of the EN Koken individuals in a broader context, we collected published genomes of pre-Bronze Age individuals from the Siberian forest-steppe zone and categorized them into distinct analysis groups based on their geographic location, time period, and positions in PC space (Fig. 2 and data S2) (*8*, *9*, *11*, *13*, *29*). Most of these individuals fall along a genetic cline connecting ANE and APS ancestries, which also reflects their relative geographic locations. We observe that they seem to form at least four distinct genetic groups on the ANE-APS cline rather than forming a continuous distribution, a pattern that seems to mirror their association with different river systems. The first group, hereafter “Tobol_HG,” includes individuals from the Tobol and Ishim river regions in western Siberia: Mergen6_HG (*n* = 2; ca. 6500 to 5500 BCE; from the Mergen 6 site in the lower Ishim River region), Sosnoviy_HG (*n* = 1; ca. 4000 BCE), and Botai_Eneolithic (*n* = 3; ca. 3500 to 3000 BCE; from the Eneolithic Botai site in northern Kazakhstan). This group, which is the geographically westernmost of the studied individuals, also shows a genetic shift toward the Eastern European Hunter-Gatherers (EEHG) in PC space. In this study, we used ancient individuals from the Mesolithic Yuzhniy Oleniy Ostrov site from Karelia (hereafter “UOO_M”; *n* = 15) as a representative of EEHG (*34*).

The second group, hereafter “Irtysh_HG_1,” comprises individuals from the Irtysh River region to the east of the Tobol/Ishim rivers: Omskaya_HG (*n* = 2; ca. 5200 to 4700 BCE; from the EN Omskaya Stoyanka II site) and Borovjanka17_HG (*n* = 1; ca. 3600 BCE; from the Eneolithic Borovjanka XVII site). The third group, hereafter “Irtysh_HG_2,” includes the remaining individuals from the Irtysh River region who show a slightly different genetic profile from that of Irtysh_HG_1: Protoka_HG (*n* = 1; ca. 3800 BCE; from the Protoka site), Vengerovo2_HG (*n* = 7; ca. 5700 to 3300 BCE; from the Vengerovo-2 site), and Koken_EN (*n* = 2). Mirroring geography, both Irtysh groups also occupy intermediate positions on the ANE-APS cline between Tarim_EMBA and Koken_EN1 in PCA.

The last group, hereafter “UpperOb_HG,” consists of individuals from the Upper Ob River region of the Altai-Sayan mountains: Altai hunter-gatherers (*n* = 5; ca. 5500 to 3400 BCE; Altai_7500BP/6500BP/5500BP), Itkul_HG (*n* = 1; ca. 4200 to 3900 BCE; from the Itkul site), UstIsha_HG (*n* = 1; ca. 5200 to 4800 BCE; from the Ust’-Isha site), and AfontovaGora_HG (*n* = 1; ca. 3900 to 3600 BCE; from the Afontova Gora site). Again mirroring their more eastern geographic location, they fall in PC space further to the right along PC1. An EBA individual from the Upper Yenisei River region, BZK002 (ca. 2800 to 2600 BCE; from the Bazaikha site), also falls on this cluster. Last, to the east of the sites of Altai hunter-gatherers, DolgoeOzero_HG (*n* = 1; ca. 4200 to 3900 BCE; from the Dolgoe Ozero site in the Upper Yenisei River region) falls in PC space between UpperOb_HG and the APS individuals.

**Fig. 3.**
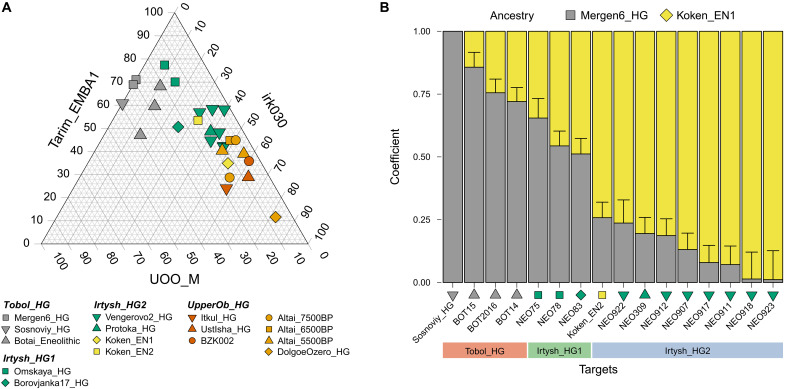
QpAdm modeling of the genetic profiles of ancient Siberian forest-steppe hunter-gatherers. (**A**) Distal three-way qpAdm admixture models of ancient Siberian forest-steppe hunter-gatherers from Tobol, Ishim, Irtysh, and Yenisei River regions (data S7D). Ancestry proportions attributed to ANE (Tarim_EMBA1), APS (irk030), and EEHG (UOO_M) were presented in the triangle plot. Three Tobol_HG individuals produced negative APS coefficients and were therefore plotted using reduced two-way models, ANE + EEHG. (**B**) Proximal two-way qpAdm models for Tobol and Irtysh hunter-gatherers (data S7F). The Sosnoviy_HG individual, which yielded a negative ancestry coefficient for Koken_EN1, was plotted using a reduced one-way qpAdm model with Mergen6_HG as the sole source. Vertical lines represent 5 cM block jackknifing standard error measures (SEM) associated with the estimated ancestry proportion. To improve visual clarity, SEM bars are displayed only on the Koken_EN1 side.

To formally characterize the genetic profiles of the Siberian forest-steppe hunter-gatherers, we performed distal admixture modeling using qpAdm, modeling each individual as a mixture of ANE, EEHG, and APS ancestries (Fig. 3 and data S7). To choose best working proxies for each ancestral component, we first conducted group-based qpAdm analyses, evaluating multiple candidate populations as potential sources. For the ANE component, we selected Tarim_EMBA1 (*n* = 12), which is the only population with a relatively large sample size among ANE-related populations. For the EEHG ancestry, we compared UOO_M and eight other Neolithic hunter-gatherer groups from Don and Volga River regions and forming the “European hunter-gatherer” and “Volga” clines according to the original study (fig. S11) (*35*). Among these eight groups, Ukrainian Neolithic hunter-gatherers (“Ukraine_N”; *n* = 35) (*35*–*37*), individuals from Sakhtysh in the Upper Volga (“UpperVolga”; *n* = 24) (*35*, *38*), and individuals from the Murzikha-2 site near the Volga-Kama confluence (“Murzikha”; *n* = 11) (*38*) form the “European hunter-gatherer cline,” running between UOO_M and Ukraine_N. The remaining five groups form the “Volga cline” running away from UOO_M toward Iranian/Caucasus populations in the following order: individuals from Ekaterinovskiy-Mys (“Ekaterinovska”; *n* = 25) (*35*), individuals from Khvalynsk with low (“Klo”; *n* = 10) (*25*, *35*), medium (“Kmed”; *n* = 9) (*25*, *35*), and high (“Khi”; *n* = 9) (*25*, *35*) affinity with those from the Berezhnovka-2 and Progress-2 sites (“BPgroup”; *n* = 5) (*35*, *39*). UOO_M emerged as the best proxy based on consistently higher qpAdm *P* values compared to other EEHG-related candidates (data S7B). For the APS ancestry, all four available APS proxies (UKY, Kolyma_M, Dzhylinda-1, and irk030) yielded similar model fits. To resolve this, we applied a rotating outgroup approach, sequentially placing each APS candidate in the outgroup set (*40*). This analysis identified irk030 as the most suitable proxy: When irk030 was placed in the outgroup, models using other APS candidates produced substantially lower qpAdm *P* values, suggesting irk030 captured the APS ancestry most effectively (data S7C).

Using these selected proxies—Tarim_EMBA1 (ANE), UOO_M (EEHG), and irk030 (APS)—we then applied three-way admixture modeling at the individual level. This approach successfully explained the ancestry of 26 of 27 individuals (qpAdm *P* > 0.05; data S7D), indicating robust model fit. Notably, the estimated ancestry proportions followed a geographic cline: EEHG contributions (UOO_M) gradually decreased from Tobol_HG (21.3 to 39.3%; 29.8 ± 2.6% as a group) to Irtysh_HG_1 (14.8 to 23.4%; 18.8 ± 3.2% as a group) to Irtysh_HG_2 (2.3 to 14.5%; 11.3 ± 2.6% as a group) to UpperOb_HG (4.2 to 18.6%; 6.5 ± 3.2% as a group), while APS ancestry (irk030) showed a corresponding increase: Tobol_HG (−9.1 to 13.6%; 8.0 ± 2.6% as a group), Irtysh_HG_1 (7.6 to 26.0%; 20.3 ± 3.3% as a group), Irtysh_HG_2 (30.9 to 52.5%; 43.0 ± 2.9% as a group), and UpperOb_HG (47.7 to 63.2%; 54.4 ± 3.3% as a group). These results clearly suggest a gradient of increasing eastern Eurasian affinity toward the east. To test whether all three sources were necessary to explain individual ancestries, we implemented nested two-way models, moving the excluded third source into the outgroup set. Consistent with the ancestry proportion estimates from the three-way model, all three components were necessary for 17 of 27 individuals (qpAdm *P* < 0.05 for both ANE + EEHG and ANE + APS models; data S7D). Those who fit the two-way models showed a clear geographic pattern: i.e., five individuals fitting without irk030 (APS) were from the west (four of six Tobol_HG and one of three Irtysh_HG_1 individuals), while the other five fitting without UOO_M (EEHG) were from the east (3/10 Irtysh_HG_2 and 2/8 UpperOb_HG individuals). When using the nested *P* value that compares the model fit between the nesting three-way and nested two-way models as a criterion, 25 of 27 individuals required the three-way model (nested *P* < 0.05 for both two-way models), while the remaining two UpperOb_HG individuals did not require UOO_M (data S7D). Overall, these results highlight the three ancestry components shaping the genetic profiles of the Siberian forest-steppe hunter-gatherers and their clinal population structure, decreasing EEHG and increasing APS ancestry proportions from west to east.

Since Koken_EN1 is the oldest genome without EEHG ancestry among Irtysh_HG_2 and Mergen6_HG is the oldest genome among Tobol_HG, we tested if we could model the later Tobol_HG and Irtysh_HG individuals using Koken_EN1 and Mergen6_HG as ancestral proxies (Fig. 3B and data S7, E to G). This proximal admixture model provides adequate results for all Tobol/Irtysh individuals even when the distal sources UOO_M and Tarim_EMBA1 are included in the outgroup set (qpAdm *P* > 0.05 for 14 of 16; data S7F). The proximal admixture model also provides adequate results when the target individuals were grouped and analyzed in the population level (data S7G).

### Two distinct admixture trajectories during the Middle/Late Bronze Age dispersal of herders

Previous archaeogenetic studies reported the population movement of MLBA herders (“steppe_MLBA”) into the western and central Eurasian Steppe (*29*), a process that is corroborated by material assemblages from the Sintashta and subsequent archaeological cultures of the Andronovo cultural community (*41*). Although their genetic similarity has led to their classification under the shared label steppe_MLBA, both subtle genetic differentiation and clearly distinct archaeological identities have contributed to the recognition of two subclusters: “western Steppe_MLBA” and “central Steppe_MLBA.” Building on this framework, we examined the genetic affinities of the Koken_MLBA individuals to assess their relationship to these subgroups (*29*). In the PC space calculated from present-day Western Eurasian individuals, we confirm that Koken_MLBA individuals cluster together with the steppe_MLBA individuals, particularly those along the central steppe cline (e.g., Krasnoyarsk_MLBA) (fig. S12). Likewise, outgroup f3 analysis shows a strong affinity between Koken_MLBA and other steppe_MLBA populations (fig. S9 and data S5). Analysis using qpWave shows that Koken_MLBA forms a clade with central steppe_MLBA populations but is distinguishable from earlier period western steppe_MLBA populations, recapitulating a previously reported pattern that reflects a subtle genetic differentiation between western and central steppe_MLBA groups (fig. S13 and data S8) (*29*). Specifically, we analyzed a set of previously published populations with at least three individuals, including central steppe_MLBA groups—Kazakh_Mys_MLBA (*n* = 4), Krasnoyarsk_MLBA (*n* = 16), Oy_Dzhaylau_MLBA (*n* = 6), and Shoendykol_MLBA_Fedorovo (*n* = 3)—and western steppe_MLBA groups—Aktogai_MLBA (*n* = 5), Corded_Ware_Czech_EN (*n* = 5), Corded_Ware_Germany (*n* = 11), Kairan_MLBA (*n* = 6), Maitan_MLBA_Alakul (*n* = 7), Petrovka (*n* = 3), Sintashta_MLBA (*n* = 37), and Srubnaya (*n* = 11) (fig. S14) (*29*). We find that Koken_MLBA is consistently cladal with central steppe_MLBA populations (qpWave *P* > 0.05 for three of four populations and qpWave *P* > 0.009 for all) but genetically distinct from western steppe_MLBA populations (2.72 × 10^−16^ < qpWave *P* < 0.022 for all eight populations). Further supporting this pattern, f4 statistics of the form f4(Mbuti, world-wide; Sintashta_MLBA, Krasnoyarsk_MLBA/Koken_MLBA) show that central steppe_MLBA groups have extra affinity with various Siberian and East Asian populations compared to western steppe_MLBA groups, implying admixture between incoming western steppe_MLBA and local hunter-gatherer groups (fig. S10 and data S6).

Using qpAdm, central steppe_MLBA groups can be adequately modeled by Sintashta_MLBA + local hunter-gatherers with around 5% contribution from the latter (data S8B). Among the tested sources, Irtysh_HG groups such as Omskaya_HG (qpAdm *P* > 0.05 for four of five populations and > 0.004 for all) and Koken_EN2 (qpAdm *P* > 0.05 for three of five populations and > 0.019 for all) provided well-fitting models (data S8B). Notably, Koken_EN1 provides a worse fit than Koken_EN2, which we attribute to its lower genetic affinity to EEHG ancestry and higher genetic affinity to APS ancestry. To test this, we introduced a third source, UOO_M, into qpAdm models (Sintashta_MLBA + Koken_EN1 + UOO_M), which yielded a better fit (qpAdm *P* > 0.05 for two of five populations) and inferred a significant UOO_M contribution of 0 to 6% (nested *P* < 0.05 for all five populations). A similar improvement was observed when using irk030 as the third source in the model (Sintashta_MLBA + Koken_EN1 + irk030), with qpAdm *P* values increasing for four of five populations. In this case, irk030 received a negative coefficient (0 to −4%), likely compensating for the excess affinity between Koken_EN1 and irk030 (nested *P* < 0.05 for all; data S8C). Although these three-way models using Koken_EN1 do not adequately portrait the ancestry of the target populations, the consistent improvements suggest that the true source, which lived in the Bronze Age and descended from the Neolithic Irtysh_HG metapopulation, carried a genetic profile more closely aligned with Koken_EN2 or Omskaya_HG but distinct from Koken_EN1.

The two genetic outliers among the MLBA Koken individuals show that steppe_MLBA herders mixed with local populations along their dispersal routes to a small degree. The f4 symmetry test between the main cluster and the outliers, in the form of f4(Mbuti, world-wide; Koken_MLBA, Koken_MLBA_o1/o2), confirms that Koken_MLBA_o1 harbors a strong extra genetic connection with ANE ancestry [e.g., f4(Mbuti, Tarim_EMBA1; Koken_MLBA, Koken_MLBA_o1) = 7.6 SEM], whereas Koken_MLBA_o2 harbors an extra affinity with the East Asian ancestries [e.g., f4(Mbuti, Ulaanzuukh_SlabGrave; Koken_MLBA, Koken_MLBA_o2) = 21.5 SEM] (fig. S10 and data S6). This suggests potential admixture events between the incoming main Koken_MLBA cluster and different groups of local hunter-gatherers. For Koken_MLBA_o1, we find that local hunter-gatherers exhibiting higher EEHG ancestry, such as Mergen6_HG, are one such possible admixture partner (qpAdm *P* = 0.266; 78.7 and 21.3% contributions from Koken_MLBA and Mergen6_HG, respectively). In contrast, hunter-gatherers with more APS ancestry, such as BZK002, are a more probable admixture partner for Koken_MLBA_o2 (qpAdm *P* = 0.801; 38.9 and 61.1% contributions from Koken_MLBA and BZK002, respectively) (data S8).

**Fig. 4.**
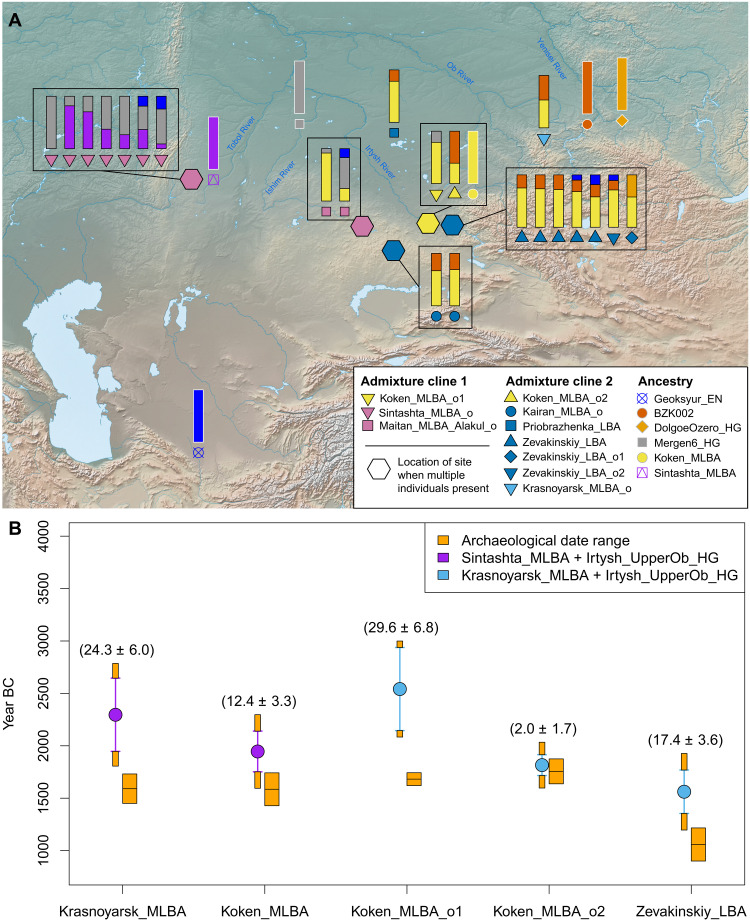
Admixture patterns observed among MLBA genetic outliers. (**A**) QpAdm admixture models for MLBA outliers, using subsets of six source populations marked by color-filled symbols with gray outlines. The geographic locations of the modeled MLBA outliers were marked by the color-filled symbols or the color-filled hexagons (for sites with multiple individuals; for these, site location is indicated by a hexagon, and the corresponding individuals are bounded by a box). In most cases, the extra genetic ancestry in these outliers was modeled as a mixture of either Tobol_HG (e.g., Mergen6_HG) or UpperOb_HG (e.g., BZK002). The basemap used in (A) is in the public domain and accessible through the Natural Earth website (https://naturalearthdata.com/downloads/10m-raster-data/; last accessed 3 March 2022). (**B**) Admixture dates of MLBA groups estimated by DATES. The orange-colored rectangles represent the archaeological date range, set as the union of 95.4% confidence intervals of calibrated radiocarbon dates of individuals in each group. The color-filled circles and the associated vertical lines represent the admixture date estimates and ±2 SEM estimated by the leave-one-chromosome-out approach. The admixture date was marked from the center of the archaeological date range. Orange blocks attached to the top and bottom of the ±2 SEM intervals of the admixture dates correspond to the half of the archaeological date range to portrait the total uncertainty of the admixture date estimates.

To understand these two outliers in a broader context of interaction between pastoralists and hunter-gatherers across the Eurasian Steppe, we revisited 20 previously published steppe_MLBA outliers (*29*) showing similar patterns on PCA. Projecting the outliers on the PCA, we identified two distinct admixture clines linking steppe_MLBA to distinct Siberian hunter-gatherer groups: one with high ANE affinity such as Tobol_HG and the other with high APS affinity such as UpperOb_HG (Fig. 2). The former cline includes outliers from the MBA Kamennyi Ambar 5 site (Sintashta_MLBA_o; *n* = 7), two individuals from the Maitan burial ground in Central Kazakhstan (Maitan_MLBA_Alakul_o; *n* = 2), and Koken_MLBA_o1. As expected from the PCA, outliers from the first admixture cline were adequately modeled by admixture between steppe_MLBA and Tobol_HG or necessitating Central Asian ancestry, represented by Geoksyur_EN, as the third component (qpAdm *P* > 0.05 for 8 of 10 individuals and > 0.035 for all 10 individuals) (Fig. 4A and data S8).

Outliers from the second admixture cline include the following groups: two outliers discovered at Kairan 1, the burial ground located in Karaganda region of Kazakhstan (Kairan_MLBA_o, *n* = 2), one individual from the Orak Burial place in the Krasnoyarsk region (Krasnoyarsk_MLBA_o; *n* = 1), one individual from the Priobrazhenka 3 burial ground in Chanovsky district of Novosibirsk region (Priobrazhenka_LBA; *n* = 1), individuals from the burial ground of Zevakinskiy in eastern Kazakhstan (Zevakinskiy_LBA; *n* = 7), and Koken_MLBA_o2. Because of the limited availability of Siberian hunter-gatherer genomes, the original study did not suggest feasible proximal models for them (*29*). In a sharp contrast to the outliers in the first cline, they cannot be modeled by a mixture of Koken_MLBA and Mergen6_HG (qpAdm *P* < 0.007 for all 12 individuals; data S8D). Therefore, we tested Koken_EN1 and BZK002 as an alternative source to represent the Irtysh_HG_2 and UpperOb_HG groups, respectively. We observe that Koken_MLBA + Koken_EN1 and Koken_MLBA + BZK002 adequately model 5 of 12 and 8 of 12 outliers, respectively (qpAdm *P* > 0.05). Adding Geoksyur_EN as the third source, both sources explain 11 of 12 outliers (qpAdm *P* > 0.05 for both Koken_EN1 and BZK002 models). The only remaining individual, I3772 from the Zevakinskiy site, could be explained only by a mixture with hunter-gatherers further to the east, such as DolgoeOzero_HG and irk030, a Late Neolithic individual from west Baikal (*28*) (qpAdm *P* > 0.05 for both models and 42 and 39% contributions from DolgoeOzero_HG and irk030, respectively; data S8). These results might be due to independent multiple admixture events with diverse eastern sources, although the existence of a population with an intermediate genetic profile cannot be ruled out.

Last, we estimated the admixture dates for central steppe_MLBA groups and the outliers from both admixture clines. To increase the resolution of admixture dating, we grouped Irtysh_HG_2 and UpperOb_HG as one group (Irtysh_UpperOb_HG). Using Sintashta_MLBA and Irtysh_UpperOb_HG as proxies for the western and eastern Eurasian sources, we obtain admixture dates around 2000 to 1500 BCE for the central Steppe_MLBA groups, as well as most outliers, which is consistent with the radiocarbon chronologies for steppe pastoralist sites across this territory (Fig. 4B and fig. S15). The admixture dates in the main central Steppe_MLBA groups seem to be slightly older than the outliers in the cline with Koken_MLBA_o2, suggesting that the hunter-gatherer admixture within this cline was unlikely a single-pulse event but rather continued for some time as more groups came into contact. In contrast, the admixture date of the Koken_MLBA_o1 cline is estimated to be around 2500 BCE, implying that admixture in this cline occurred only during the initial stage of the MBA in the steppe zone.

### Nonprimary burial contexts for some EN and MLBA crania

During excavation, two crania at Koken were found in nonprimary burial contexts, one dating to the EN (KKN001) and the other to MLBA (KKN091). The EN human remains at Koken were recovered from a single burial containing a cranium (KKN001) and postcranial remains (KKN002) that otherwise lacked grave goods or subsurface or surface architecture (*24*). Although the remains initially appeared to make up a single individual, differences in age-at-death profiles determined through epiphyseal fusion and dental development patterns indicated that the cranial and postcranial remains likely originated from two different individuals. The cranium of KKN001 was that of an adolescent, while the postcranial remains of KKN002 belonged to an older adolescent or young adult. The KKN001 cranium was placed next to the ribcage of KKN002 and oriented facing northwest, while KKN002 was laid on the right side in an extremely flexed position, suggesting binding or wrapping, and oriented to the northeast. Most of the skeletal elements from KKN002’s left side were missing, but the remaining elements were recovered in anatomical position with only minimal displacement and no evidence of subsequent intrusive activities. Genetic analysis confirmed that the cranium and postcranial remains originated from two distinct males who were paternal second-degree relatives, possibly an uncle/nephew or half-brother pair (fig. S7 and data S4). Together, it appears that the remains of the male relatives were collected and intentionally buried together, although the cultural context for the joint burial and the incomplete nature of their skeletons is unclear.

The second cranium found in a nonprimary context appears to be the consequence of ancient looting. MLBA burials at Koken consist primarily of inhumations, with a single instance of cremation (figs. S3 to S6). The human remains were typically placed within stone slab cists and accompanied by grave goods such as ceramic vessels, metal jewelry, and animal offerings. Burial pits vary in depth (30 to 150 cm) and are marked by a stone ring or rectangular stone fence on the ground surface. Because of their high visibility on the landscape, such burials both at Koken and elsewhere are frequently recovered in a looted state, and crania appear to have been specifically targeted by looters (see Supplementary Text) (*42*, *43*). At Koken, 20 of the 22 examined MLBA burials were looted. Of the two intact burials, looters had attempted to open one of them (KKBR13) but stopped before reaching the cist at the base of the 150-cm pit that contained adult female KKN090. A cranium (KKN091) was found in the fill above the cist, which appears to be associated with the attempted looting. An adjacent burial (KKBR12) had been looted to completion and was found to contain a complete postcranial skeleton (KKN089) but no cranium. KKN089 and KKN091 were found to have identical genetic profiles (data S4), allowing us to identify KKN091 as the cranium removed from KKBR12. The reconstruction of the events leading to the relocation of crania offers some insight into looting practices that have challenged archaeological investigations in the region.

## DISCUSSION

In this study, we provide a genome-wide ancestry analysis that places EN and MLBA individuals at Koken in the broader context of hunter-gatherer and pastoralist genetic diversity across the Eurasian steppe. While dynamic changes in hunter-gatherer genetic profiles have been intensively studied in the west Baikal region (*9*–*11*, *17*, *28*), the diversity of hunter-gatherer genetic profiles and their distribution in space and time have been only sporadically explored in the vast region of the central Eurasian Steppe and Western Siberia. By reconstructing EN genomes from the far Upper Irtysh River region and coanalyzing them with recently published prepastoralist genomes across the forest-steppe zone of Western Siberia (*8*), we characterize the structure of hunter-gatherer populations in this region and illuminate their stratification by river regions. Specifically, we show the genetic influence from EEHG and APS ancestries follows a clinal pattern, highest in the western and eastern regions, respectively. For example, the western-most Tobol_HG group was largely modeled as ANE + EEHG without APS ancestry, while a majority of individuals from the eastern groups, Irtysh_HG_2 and UpperOb_HG, were modeled as ANE + APS without EEHG ancestry (Fig. 3 and data S7). We also showed that a mixture between two hunter-gatherer groups, represented by the early members of Tobol_HG and Irtysh_HG_2 (Mergen6_HG and Koken_EN1, respectively), led to the formation of later Tobol_HG and Irtysh_HG_1 groups, including the famous Eneolithic Botai population from northern Kazakhstan (Fig. 3B). Our observation of hunter-gatherer genetic stratification by river regions within Inner Asia is also consistent with current archaeological understanding of hunter-gatherer mobility and lifestyle, which favored seasonal habitation within the riverine basins and tributary river systems of the steppe that contained key freshwater and terrestrial resources (*44*–*46*). It was not until the Bronze Age that newly formed populations intensified their use of these landscapes. With the rise of pastoralism and new means of mobility, a new settlement pattern appeared throughout the steppe and taiga (*47*–*51*).

The EN burial at Koken has no contemporaries to date within Kazakhstan, and thus, the archaeological context of the burial remains enigmatic. Radiocarbon dates place both EN individuals during the mid-sixth millennium BCE while also revealing a plausible generational difference in their time of death (Fig. 1B); however, we cannot exclude that they were contemporaries. The two individuals were second degree relatives who both died at relatively young ages: KKN001 (Koken_EN1) died during adolescence and KKN002 (Koken_EN2) died during late adolescence or early adulthood. Their different radiocarbon dates and only partially complete burials may be an indication that the burials were reopened and added to or had elements removed and relocated over time. The knowledge that they were relatives further indicates that shared kinship was a factor in how and on whom such mortuary activities were performed. The burial could have taken place in stages several years apart by people who knew both the deceased relatives. The close genealogical ties identified between the two EN individuals from Koken may be an indication that kinship played a role in how people were buried and with whom.

In the Baikal region of Siberia and in northern Mongolia, there are EN burial grounds with comparable traits to EN Koken (*18*, *52*, *53*). The tradition of burial in a strongly flexed position and the presence of single and joint burials involving two or more individuals are two notable similarities. An additional analogy with the Baikal region is the practice of burying skeletons without a skull, the secondary removal of skulls from graves, or the burial of skulls separately, as well as the secondary and later burial of other people’s human bones or individual body parts (*52*). In particular, the absence of a skull is one of the main features that characterize the EN burials of Lake Baikal, along with the lack of disturbance to the postcranial skeleton. Overall, these deliberate postmortem manipulations of the body before and after burial align with the mortuary features observed in the EN burial at Koken. The Koken individuals likely died at different times, and each is represented by an incomplete skeleton—one missing a head and the other missing a body, and so appear to represent two burial episodes in which graves were reopened and new elements were added or removed.

The spread and appearance of pastoralism in the central Eurasian Steppe during the MLBA yielded visible demographic and cultural changes, reflecting a complex process that involved both substantial population movements and localized interactions, as evidenced by west-to-east gene flow in the second millennium BCE (*2*, *12*, *29*, *54*). Our analysis of the MLBA Koken individuals supports this view: All but two of the MLBA Koken individuals form a genetically homogeneous cluster that is cladal with previously reported MLBA central Eurasian Steppe individuals (data S8). We found no close familial relationships (up to the third degree) among the MLBA individuals at Koken, even among individuals buried together in double, triple, and cluster graves (data S4C). There also does not appear to be any spatial pattern with respect to sex. While females outnumber males 5:2, both sexes are present in all studied areas of the cemetery area, and individuals of all ages are represented, from approximately age 1 to >60 years. This varied arrangement of individuals within a single cemetery alludes to the dynamic social structure at MLBA Koken. Although most graves had been looted, several graves were unlooted or sufficiently undisturbed (see Burial 13 and Burial 15 in the Supplementary Materials) so that excavations at Koken were able to document typical mortuary practices of the period, such as the use of subterranean stone cists inside an above ground stone fence, the arrangement of individuals on their side in a flexed position with head to the west, and placement of grave goods consisting usually of jewelry and ceramic vessels.

Despite a growing body of genetic data for MLBA steppe populations, however, one question that has remained unclear is which Early and Middle Bronze Age (EMBA) pastoralists and hunter-gatherers occupied the central Eurasian Steppe before the influx of new populations, and what demographic and cultural dynamics emerged from their encounters. Genetic outliers found at MLBA sites may provide clues to resolve these questions because they are presumably at least partial descendants of the EMBA populations in the region. Among our MLBA Koken individuals, there are two genetic outliers, both adult females: KKN094 (Koken_MLBA_o1) and KKN099 (Koken_MLBA_o2). They were buried in a manner consistent with pastoralist burials of the central and eastern steppe, alongside other individuals of the main Koken_MLBA cluster, mirroring previously reported MLBA genetic outliers (*29*), who were also buried in cemeteries following the same mortuary traditions as nonoutlier individuals. Such an observation draws attention to the existence of smaller-scale processes of demographic integration in the steppe that have received less attention than broad scale population mobility. Diverse genetic backgrounds were not a barrier to cultural integration as multiple distinct populations appear to have exchanged genes and coexisted in the area of Koken. At Koken, each outlier has an ancestry profile reflecting a distinct admixture cline diverging from the main steppe MLBA genetic profile (Fig. 4 and data S8).

After producing a fine-scale characterization of Inner Asian hunter-gatherer genetic profiles, we can now narrow down the likely sources of hunter-gatherer ancestry in these two genetic outliers. Koken_MLBA_o1 has greater ANE ancestry, pointing to a more western population, such as near the Tobol or Ishim Rivers, while Koken_MLBA_o2 has greater East Asian ancestry, such as that found near the Irtysh, Ob, and Yenisei Rivers. While admixture between incoming and local populations appears to have occurred to a limited degree (contributing a small amount of DNA to the descendant population), it continued to take place over a long period of time, a trend that is reflected in the more westward and eastward genetic affiliation of Koken_MLBA_o1 and Koken_MLBA_o2, respectively. The drawn-out nature of these interactions is also reflected in the radiocarbon chronologies: Outlier individuals come from archaeological contexts ranging from the early quarter of the second millennium BCE (e.g., Sintashta_MLBA and Maitan_MLBA), through the mid-second millennium BCE (Kairan_MLBA, Koken_MLBA_o1, and Koken_MLBA_o2), to the first century of the first millennium BCE (e.g., Zevakinskiy burial ground and Zevakinskiy_LBA). While the precise identity of the specific EMBA source populations still remains obscure due to limited sampling of ancient genomes from this time period, our study provides valuable information linking them to their prepastoralist ancestors.

The multiperiod site of Koken in eastern Kazakhstan has yielded valuable information clarifying the genetic structure of hunter-gatherer populations in the central Eurasian Steppe, as well as their genetic legacy in the millennia-later MLBA pastoralist populations in the region. Our study of Koken illuminates the potential of a growing inventory of ancient genomes in revealing the rich history of human mobility and mixing intertwined with biogeography and major cultural transformations in Inner Asia. Future studies in the region—particularly those focusing on the EMBA period—will help clarify small-scale processes of demographic integration among diverse populations across the interconnected Eurasian steppe and shed light on one of its most consequential transformations: the introduction of pastoralism.

## MATERIALS AND METHODS

### Ethics and inclusion statement

Archaeological research, project design, and sampling were carried out in collaboration with local specialists and through partnerships with local laboratories, museums, and research institutions. Local researchers contributed to the study as coauthors. Sampling for export and analysis was approved as part of the permissions processes described below. The skeletal remains from Koken in Kazakhstan’s Abay region were excavated between 2019 and 2022 by P.D.D., A.Z., Z.T., E.R., and G.K. under permits issued by the authority Ministry of Culture and Sport of the Republic of Kazakhstan to Nazarbayev University on 10.6.2019 (permit no. 19012480) and to the Margulan Institute of Archaeology on 17.8.2021 (permit no. 21024060). Sampling of bone and teeth for archaeogenetic analysis was granted under a preexisting Memorandum of Cooperation Directum-27926-3298740 established on 22 July 2022 between The Autonomous Organization of Education “Nazarbayev University” and the Max Planck Institute for Evolutionary Anthropology. All remains reported in this study are housed in the Anthropology Laboratory (Principal Investigator: P.D.D.) at Nazarbayev University in Astana, Kazakhstan. Specimen identifiers for this project are as follows: KKOP6_ctx29H.1 (KKN002; KKN014); KKOP6_ctx29H.2 (KKN001); KKBR3 (KKN083); KKBR4 (KKN084); KKBR5 (KKN085); KKBR8 (KKN086); KKBR9 (KKN087); KKBR10 (KKN088); KKBR12 (KKN089); KKBR13.1 (KKN090); KKBR13.2 (KKN091); KKBR14.1 (KKN092); KKBR14.2 (KKN093); KKBR15.1 (KKN094); KKBR15.2 (KKN095); KKBR16 (KKN096); KKBR17 (KKN097); KKBR18 (KKN098); KKBR19 (KKN099); KKBR20 (KKN100); KKBR21 (KKN101); and KKBR22 (KKN102). P.D.D., Associate Professor at Nazarbayev University, is the contact person and curator of the Anthropology Laboratory where the items are stored (paula.dupuy@nu.edu.kz).

### Sample provenance

Koken is located along the northern edge of the Kokentau foothills situated 80 km southwest of the city of Semey, in the Abai region of eastern Kazakhstan. Located in the semiarid steppe zone, the Kokentau mountain range forms a natural outcrop of granitoids stretching from northwest to southeast for 15 km, reaching a height of 800 m above sea level. Several small streams flow through and adjacent to the foothills and connect to the Chagan River that feeds into the larger Irtysh. The immediate environment surrounding the foothills consists of a biodiverse ecosystem of steppe grasslands, seasonal streams and springs, birch and juniper groves, marshland, and mineral outcrops. The Koken archaeological site is located on the northern face of the hills and contains an archaeological palimpsest of human activities spanning the Epipaleolithic through to the present day. Until around 30 years ago, a small village was located on and around the prehistoric settlement and burial grounds, but today aside from a few remaining homesteads, the area is used as grazing and watering grounds for sheep and cattle. The prehistoric settlement is located alongside a natural springhead and network of seasonal streams and marshland. Excavations were carried out at the Koken settlement and neighboring graveyard (Koken-3) from 2019 to 2022. The graveyard is located ~200 m to the southwest of the settlement along a stretch of marsh and on the first terrace of a dry streambed. On the site of settlement excavations, the most recent building occupations are from within the past 200 years under which are earlier stone buildings and installations spanning the second to third millennium BCE (late to EBA) as reflected in the radiocarbon dates (table S1) and material culture obtained. Beneath the Bronze Age architectural layers are stratified cultural sediments containing remains of stone industries from consecutive Stone Age periods (Eneolithic to Epipaleolithic). The EN burials were buried and sealed within the site’s Mesolithic layer. The MLBA graveyard contains grave structures consistent with styles attributed regionally to the Fedorovka (Andronovo family) Culture. The bioarchaeological assessment of human remains from the Koken site was performed by E. Bullion and L. Lacher, the team’s bioarchaeologists.

### Radiocarbon dating

We provide 10 radiocarbon dates produced from human bone collagen for individuals included in the archaeogenetic study (Fig. 1B and table S1). Four of the dates were published previously (EN = 1; MLBA = 3) (*23*, *24*). Six are new for this study (EN = 2; MLBA = 4). Bone sampled for radiocarbon AMS dating was processed at the labs: Beta Analytic, International Chemical Analysis Florida, Center for Applied Isotope Studies at the University of Georgia, and the Curt-Engelhorn-Zentrum Archäometrie (CEZA). Results were calibrated in OxCal online using the IntCal13 atmospheric curve (*55*). Dates are presented as uncalibrated BP and calibrated BCE (1σ and 2σ) in table S1.

### DNA laboratory procedures

We extracted genomic DNA and prepared 24 single-stranded DNA sequencing libraries from 21 individuals using the protocol “Bravo workstation: automated single-stranded DNA library preparation (ssDNA2.0) V.1” (dx.doi.org/10.17504/protocols.io.kqdg32bdpv25/v1). During the library preparation, we double indexed the libraries by adding unique eight-mer index sequences at both P5 and P7 Illumina adapters (*56*). These DNA extraction and library preparation were conducted in a dedicated clean room facility at the Max Planck Institute for Evolutionary Anthropology following the previously published protocols (*57*). Then, we performed shallow shotgun sequencing of these libraries to screen for DNA preservation. All 21 individuals yielded sufficient human DNA for further analysis (>1%) when mapped on hs37d5, the human reference genome GRCh37 with decoy sequences. We enriched all libraries for 1,233,013 nuclear SNPs (1240K) by applying in-solution DNA capture techniques with oligonucleotide probes targeting the 1240K sites (*25*). The captured libraries were sequenced on multiple platforms: on an Illumina HiSeq 4000 at the Max Planck Institute for Evolutionary Anthropology to generate 76-bp single-end (SE76) reads and on an Illumina NovaSeq X Plus at the same institute to produce 100-bp paired-end (PE100) reads. We deeper sequenced one shotgun library (KKN001.C0101) at the Bauer Core Facility of Harvard University on an Illumina NovaSeq 6000 S4 flowcell to generate 101-bp paired-end (PE101) sequences. The output sequences were demultiplexed allowing at most one mismatch in each index. We report the details of the sequencing in data S1.

### DNA sequencing data processing

Raw sequencing reads were first processed to remove Illumina adapter sequences using AdapterRemoval v2.3.1. For paired-end (PE) sequences, read pairs overlapping 11 bp or longer were merged into single reads. Merged and/or trimmed reads of 35 bp or longer were then aligned to the human reference genome with a decoy sequence (hs37d5) using the bwa-aln and bwa-samse modules of the Burrows-Wheeler Aligner (*58*). Default parameters were modified by disabling seeding (“-l 9999”) and allowing extra mismatches (“-n 0.01”). Before genotyping, we used DeDup v0.12.8 (*59*) to remove duplicate reads from polymerase chain reactions and filtered out low-quality reads with a Phred-scaled mapping quality score below 30 using SAMtools v1.19.2 (*60*). From these BAM files, random pseudo-haploid genotype data for the 1240K panel were generated by selecting one high-quality base (Phred-scaled base quality score of 30 or higher) per site using pileupCaller v1.5.2 with the “singleStrandMode” option (https://github.com/stschiff/sequenceTools; v1.5.2 last accessed at 19 April 2023). This approach helps minimize the effect of chemical damage by excluding forward strand reads for C/T SNPs and reverse strand reads for G/A SNPs. The 1240K genotype data of Koken individuals were merged with the published 1240K data of ancient individuals (data S3) (*2*–*5*, *7*–*13*, *17*, *25*, *27*–*29*, *34*–*39*, *61*–*84*).

We then intersected the 1240K genotype data of ancient individuals with two modern global datasets: (i) the 1240K genotype data from the Simons Genome Diversity Project (*85*) and (ii) a broader dataset from the Affymetrix Axiome Genome-wide Human Origins 1 array (“HumanOrigins”) (*1*, *3*, *32*, *61*). For allele frequency-based analyses, we primarily used the 1240K dataset, while the HumanOrigins dataset was used for PCA. For the Koken_EN1 and Koken_EN2 individuals, genome imputation and phasing were performed using GLIMPSE v2.0.0 (*86*). Initially, BAM files were split into forward and reverse reads using SAMtools v1.19.2. Genotype likelihoods at biallelic sites from the 1000 Genomes Project were then computed using bcftools mpileup v1.19 with the “-I -E -a ‘FORMAT/DP’” option, followed by variant calling with “bcftools call -Aim -C alleles” (*87*). To further minimize the effect of chemical damage, forward strand reads for C/T SNPs and reverse strand reads for G/A SNPs were excluded. Chromosomes were divided into 2-Mb segments with a 200-kb buffer region on each side using GLIMPSE2_chunk, and each segment was imputed with GLIMPSE2_phase using default settings. The imputed segments were then merged using GLIMPSE2_ligate. For the high-coverage library (KKN001.C0101.SG1), diploid genotypes were called from the imputed VCF file, considering only genotypes with a genotype probability value of ≧0.99.

### Ancient DNA authentication

We assessed the pattern of postmortem chemical damage by calculating the proportion of C-to-T misincorporations at both the 5′ and 3′ ends of the sequencing reads using the mapDamage program v2.2.1 (*88*). Our analysis revealed that all samples showed significant postmortem damage, consistent with the expected patterns from the non-UDG (no treatment with uracil-DNA-glycosylase) single-stranded library preparation method. To estimate mitochondrial DNA contamination in all individuals, we used Schmutzi v1.5.7 (*89*), running the contDeam and schmutzi modules against a global allele frequency database comprising 197 individuals. Last, we estimated the rate of nuclear contamination in male individuals using the contamination module in ANGSD v.0.941 (*90*). This estimation is based on the principle that males have only one X chromosome, so any contamination would result in additional mismatches at SNP sites, but not in the surrounding monomorphic regions.

### Genetic relatedness analysis

We used PMR (*30*) and ancIBD (*31*) to assess genetic relatedness between ancient individuals. The PMR was calculated for all pairs of ancient individuals in this study using autosomal SNPs from the 1240K panel. To further investigate familial relationships, particularly for the second-degree relatives Koken_EN1 and Koken_EN2, we used ancIBD v0.5 to estimate the distribution of shared IBD segments. The imputed VCF file from GLIMPSE2 was first converted into an hdf file using the “ancIBD.IO.prepare_h5.vcf_to_1240K_hdf” function. We then ran the “ancIBD.run.hapBLOC_chroms” function. To distinguish between different second-degree relationships, we produced 100 simulations for each of the four different second-degree relationships (grandparent-grandson, avuncular, half-siblings, and double first cousins) where we retrieve the IBD segment lengths and counts. For simulation, we used Ped-sim (*91*) and incorporated a sex-specific recombination map (*92*) with a crossover interference model (*93*). Ped-sim returns information for each pair of relationships, such as the physical and genetic range of each IBD block, as well as whether the two individuals share only one (IBD1) or both chromosomes (IBD2) in the given range. To count alternating IBD1 and IBD2 segments as one consecutive block, we used a custom R script to merge adjacent IBD1 and IBD2 segments in the Ped-sim results. In addition, to match the filtering thresholds of ancIBD to reduce false IBD calls, IBD segments shorter than 12 centimorgan (cM) or containing fewer than 220 SNPs/cM (based on the 1240K SNP panel) were filtered out. For assessing consanguineous pairing in the recent ancestors of the Koken individuals, we identified runs of homozygosity (ROH) blocks, the genomic regions devoid of heterozygotes, using hapROH program (*94*). Long ROH blocks suggest that an individual’s parents were closely related, whereas short ROH blocks are indicative of limited population size.

### Sex determination and uniparental haplogroup assignment

To determine the genetic sex of each individual, we calculated the ratio of sequence coverage between the sex chromosomes and autosomes. Typically, the X-to-autosomal coverage ratio is approximately 0.5 for males and around 1 for females, while the Y-to-autosomal coverage ratio is about 0.5 for males and close to 0 for females. Individuals with a Y-to-autosomal coverage ratio greater than 0.3 were classified as male, while those with a ratio below 0.1 were classified as female (data S1). We also identified the uniparental haplogroups for each individual. Mitochondrial consensus sequences with a quality score of 10 or higher were generated using the log2fasta program in the Schmutzi package (*89*), and haplogroups were assigned using HaploGrep v2.1.20 (data S1) (*95*). For the eight males in the study, we called 13,508 Y chromosome SNPs from the International Society of Genetic Genealogy database using the “majorityCall” option in pileupCaller v1.5.2 and assigned Y-chromosomal haplogroups using a modified version of the yHaplo program (*96*) (https://github.com/alexhbnr/yhaplo; version 2016.01.08, last accessed 28 April 2022).

### Principal components analysis

We performed PCA on present-day individuals genotyped with the HumanOrigins array using the smartpca v18140 software from EIGENSOFT v8.0.0 (*26*). Two population sets were analyzed: the first included present-day Eurasian and American individuals (*n* = 2270) and the second included Western Eurasians (*n* = 1238). Ancient individuals, not included in the initial PC calculation, were projected onto the existing PCA using the “lsqrproject: YES” option. The individuals and populations included in the PCA analysis are listed in data S3.

### *f*-Statistics

We computed *f*-statistics using the qp3pop and qpdstat functions from the R library ADMIXTOOLS2 v2.0.0 (https://github.com/uqrmaie1/admixtools) (*97*), with the following options: poly_only = F, blgsize = 0.05, verbose = T. Outgroup-f_3_ statistics were calculated using the central African population Mbuti as an outgroup to assess shared genetic drift between the target populations. Similarly, Mbuti served as the outgroup for calculating f_4_ statistics in the form of f_4_(Mbuti, X; target1 and target2), which were used to test for symmetry between targets or to identify additional sources of admixture. The populations included in the *f*-statistics analysis are listed in data S3, with the results summarized in data S5 and S6.

### Genetic admixture modeling with qpWave and qpAdm

We performed admixture modeling analysis using the qpwave and qpadm functions from the R library ADMIXTOOLS2 v2.0.0. For both qpWave and qpAdm analyses, we used the following populations as the base outgroup set: present-day Central African hunter-gatherers (Mbuti, *n* = 5), Taiwanese Aborigines (Ami, *n* = 2), Native Americans (Mixe, *n* = 5), Indigenous Andamanese islanders (Onge, *n* = 2), EN Iranians from the Ganj Dareh site (Iran_N, *n* = 8) (*3*, *29*), Epipaleolithic European (Villabruna, *n* = 5) (*17*, *34*), and EN farmers from western Anatolia (Anatolia_N, *n* = 23) (*25*). In addition, when multiple admixture models were possible, we applied the qpAdm rotating approach, which systematically shifts candidates between source and outgroup, to identify the best-fitting proximal source (*40*).

### Admixture dating with DATES

We used DATES v.753 to estimate the timing of admixture events in ancient populations, based on pseudo-haploid genotype data (*98*). This analysis was conducted under the simplified assumption that gene flow occurred as a single event, with a generation time of 29 years. DATES estimates the admixture time by measuring the decay of ancestry covariance and provides jackknife standard errors. In the parameter file for DATES, we consistently used the following options: binsize: 0.001, maxdis: 0.5, runmode: 1, qbin: 10, and lovalfit: 0.45. For each target population, we selected a pair of reference populations identified as suitable sources in the qpAdm analysis. In cases where the qpAdm source population had limited sample size or SNP coverage, we grouped multiple populations with similar genetic profiles to increase the statistical power of the DATES analysis. For Koken_MLBA and Krasnoyarsk_MLBA, we used Sintashta_MLBA (*n* = 36) (*29*) and Irtysh_HG_2 + UpperOb_HG (*n* = 18) (*8*, *11*, *13*) as references. For Koken_MLBA_o1, we used Krasnoyarsk_MLBA (*n* = 16) (*29*) and Tobol_HG (*n* = 6) (*9*, *29*) as references. For Koken_MLBA_o2 and Zevakinskiy_LBA, we used Krasnoyarsk_MLBA (*n* = 16) and Irtysh_HG_2 + UpperOb_HG (*n* = 18) as references.

## Supplementary Materials

### The PDF file includes:

Supplementary Text Figs. S1 to S15 Table S1 Legends for data S1 to S8 References
