# Supplementary material for: Ancient genomes from eastern Kazakhstan reveal dynamic genetic legacy of Inner Eurasian hunter-gatherers
Source: Sci Adv. 2025 Oct 15;11(42):eadw8219. doi: 10.1126/sciadv.adw8219 (PMC12526762; doi:10.1126/sciadv.adw8219)
Supplement: Supplementary file 1 — Supplementary Text Figs. S1 to S15 Table S1 Legends for data S1 to S8 References [file sciadv.adw8219_sm.pdf]

Science Advances
AAAS

Supplementary Materials for

Ancient genomes from eastern Kazakhstan reveal dynamic genetic legacy of Inner Eurasian hunter-gatherers

Haechan Gill *et al.*

Corresponding author: Choongwon Jeong, cwjeong@snu.ac.kr; Christina Warinner, warinner@fas.harvard.edu; Paula Doumani Dupuy, paula.dupuy@nu.edu.kz; Maxat Zhabagin, mzhabagin@gmail.com

Sci. Adv. **11**, eadw8219 (2025)
DOI: 10.1126/sciadv.adw8219

The PDF file includes:

Supplementary Text
Figs. S1 to S15
Table S1
Legends for data S1 to S8
References

Other Supplementary Material for this manuscript includes the following:

Data S1 to S8

Supplementary Text

Provenance

Initial research for this project was carried out in Kazakhstan's Abay region at the Koken settlement and neighboring graveyard (Koken-3) from 2019-2022. The skeletal remains from Koken were excavated between 2019-2022 by Paula Dupuy, Aidyn Zhuniskhanov, Zhuldyz Tashmanbetova, Erbolat Rakhmankulov, Galymzhan Kiyasbek, Elissa Bullion and Laura Lacher under permits issued by the authority Ministry of Culture and Sport of the Republic of Kazakhstan to Nazarbayev University on 10.6.2019 (Permit No. 19012480) and to the Margulan Institute of Archaeology on 17.8.2021 (Permit No. 21024060). The human remains were validated for age authenticity through 10 radiocarbon dates obtained from human bone collagen of individuals included in the archaeogenetic study (Fig. 1B; table S1). Bone sampled for radiocarbon AMS dating was processed at the labs: Beta Analytic, International Chemical Analysis Florida, Center for Applied Isotope Studies at the University of Georgia, the Curt-Engelhorn-Zentrum Archäometrie (CEZA). Results were calibrated in OxCal online using the IntCal13 atmospheric curve (*55*). Paula Dupuy, Associate Professor at Nazarbayev University in Astana, Kazakhstan, is the contact person and curator of the Anthropology Laboratory where the human remains are stored (paula.dupuy@nu.edu.kz). Specimen identifiers for this project are: KKOP6_ctx29H.1 (KKN002; KKN014); KKOP6_ctx29H.2 (KKN001); KKBR3 (KKN083); KKBR4 (KKN084); KKBR5 (KKN085); KKBR8 (KKN086); KKBR9 (KKN087); KKBR10 (KKN088); KKBR12 (KKN089); KKBR13.1 (KKN090); KKBR13.2 (KKN091); KKBR14.1 (KKN092); KKBR14.2 (KKN093); KKBR15.1 (KKN094); KKBR15.2 (KKN095); KKBR16 (KKN096); KKBR17 (KKN097); KKBR18 (KKN098); KKBR19 (KKN099); KKBR20 (KKN100); KKBR21 (KKN101); KKBR22 (KKN102).

Archaeological Site and Sample Information

Site: Koken
Country: Kazakhstan
Region: Abay Oblast
Coordinates: 49°51'51.5"N 79°36'00.4"E
Excavation details: Excavation 'TEECA', license №19012480 and №21024060 (P.N. Doumani Dupuy). Bioarchaeological information provided below is based on site reports outlining osteological features of all human remains from Koken (*100, 101*).

1.1 Early Neolithic (EN) Individuals

The Koken settlement is multilayered with stone industries indicative of the Epipaleolithic to Early Bronze Age. The stone tool assemblage consists of a high proportion of partially worked stones compared to finished items that together indicate stone working activity. The materials share broad parallels to materials from the Irtysh Region, east Kazakhstan and southern Russia. Yet among the Koken sequence, only a negligible number of materials are associated with the Neolithic. They do not reveal details of the stone industry tradition and cannot be connected with the burial with certainty. The archaeological context of the EN burial therefore remains open to future research.

1.1.1 Neolithic burial

KKOP6_ctx29H.2 (KKN001) and KKOP6_ctx29H.1 (KKN002 and KKN014)
Remains of two EN individuals were recovered by chance in the Koken settlement in a burial deposited in a cultural layer attributed to the Mesolithic based on radiocarbon AMS dates of bone from these layers and from the recovered stone tool inventory (fig. S1). The area where the EN individuals are buried is outside the vicinity of the larger MLBA cemetery discussed in the following section. The EN burial lacks grave architecture or grave goods. Two partial male skeletons were buried together that are radiocarbon dated to the Early Neolithic (EN) period (mid-6th millennium BC) (table S1). Individual KKN001 consists of a cranium and individual KKN002/KKN014 (hereafter KKN002) consists of postcranial remains. The KKN001 cranium was placed next to the ribcage of KKN002 and oriented facing northwest, while KKN002 was laid on the right side in an extremely flexed position and oriented to the northeast. Most of the skeletal elements from KKN002's left side were missing, but the remaining elements were recovered in anatomical order with only minimal displacement of skeletal elements and no evidence of subsequent intrusive activities. A detailed discussion with archaeological interpretations of EN Koken in local and regional context is provided in a separate publication (*24*). As of 2025, excavations at the Koken settlement cover an area of ~200 square meters and have yielded cultural layers spanning the Ethnographic to Epipaleolithic period. No additional Neolithic burials have been encountered within the settlement area despite intensive exploration in the zones adjacent to the discovery. A geomagnetic survey was also conducted and while informative for exploring the sprawling Bronze Age architecture, it did not produce significant results for the Neolithic aspect of the research. Investigations at Koken are ongoing, but presently its EN burials represent the only two of their kind in Kazakhstan. For this study, two bone samples from KKN002 were analyzed (KKN002, tibia; KKN014, phalanx) and confirmed to belong to the same individual. Taken together, it appears that the cranial remains of KKN001 and the postcranial remains of KKN002 were collected and intentionally buried together, although the cultural context for the joint burial and the incomplete nature of their skeletons is unclear. Archaeogenetic analysis determined that the individuals are 2nd degree male relatives. KKN001 as an adolescent. KKN002 was an older adolescent or young adult.

- Pathologies: None detected.
- Osteological sex: KKN001, undetermined. KKN002, possible male.
- Genetic sex: Both male.
- Radiocarbon date: Two AMS dates for KKN001 give the range 5479-5369 cal BC and one AMS date for KKN002 potentially places it slightly younger, at 5471-5222 cal BC (table S1).

1.2 Middle-Late Bronze Age (MLBA) individuals
Three MLBA graveyards were identified at Koken, and the MLBA individuals analyzed in this study were excavated from the third graveyard, Koken-3 (fig. S2). The Koken settlement and the cemetery are located ~250 meters away from each other. Graves contain architectural and burial elements that reflect the variability present among the burial traditions documented for the Andronovo Cultural Horizon, specifically the Fedorovka variant of this culture. Unless otherwise stated in the summary below, the grave architecture consists of an above ground fence built from large stones enclosing a subterranean cist constructed from stone slabs placed into the ground on their vertical end and capped with a stone lid. Some burials are arranged as a fence enclosing a single grave containing one individual (fig. S3), while other burials consist of a fence enclosing a

double grave, with two individuals each buried within their own cist (fig. S4). Lastly, the graveyard also includes clusters of burials where the fences intersect or were placed very close together (figs. S5-S6).

MLBA stone fences are variously oval, round, or rectangular. Burial cists orient West-East, and bodies were placed on their left side in a flexed position, with heads oriented west. Grave goods are described individually below. In addition to grave goods, Stone Age lithics were commonly encountered within the burial sediments of the Koken graveyard, but these are likely incidental given that lithic materials are a ubiquitous feature of the surface deposits at Kokentau. Consequently, stone objects recovered from the graveyard were assumed to be incidental and not related to the MLBA grave assemblage.

1.2.1 Singular Graves

Grave No. 3: KKBR3 (KKN083)

Grave No. 3 contained an inhumation of a child aged 4-5 years (KKN083) along with a cervical vertebrae and middle rib fragment of an adult (not included in study). Individual KKN083 is represented by a limited number of cranial remains and mostly complete postcranial skeleton. The tomb was disturbed in ancient times and the bones displaced from their original context. However, metacarpals and hand phalanges were recovered in situ, so it is possible that the disturbance occurred after skeletonization was advanced or completed. The stone cap on the burial cist was broken in two, presumably during the ancient looting episode. The above ground fence is rectangular and encloses an area of 4 sq. meters. No grave goods were encountered.

- Pathologies: None detected
- Osteological sex: Not determined
- Genetic sex: Male.
- Radiocarbon date: None

Grave No. 4: KKBR4 (KKN084)

Grave No. 4 contained an inhumation of an adult aged 50+ at time of death (KKN084). A nearly complete cranial and postcranial skeleton was recovered on its left side in a flexed position, head oriented west. The individual displayed a healed injury to the left arm. The above ground fence is circular and covers an area of 4 sq. meters. The tomb was disturbed in ancient times, but minimal visible damage was caused aside from the stone lid being broken into several pieces. At the time of excavation, groundwater had risen and entered the burial chamber resulting in some skeletal elements being displaced. A medium ceramic pot (банка) sat behind the cranium in the northwest corner of the burial cist. The pot measures 12.5 cm high and has a rim diameter of 14 cm and base diameter of 9 cm. A non-uniform combination of incised cross-hatch and herringbone patterns appear around the neck.

- Pathologies: Left ulna displays a fully healed, non-union fracture. Eburnation on the distal left humerus indicates that this fracture damaged the elbow joint, but also reflects continued use of the joint.
- Osteological sex: Probable female
- Genetic sex: Female
- Radiocarbon date: 1507-1407 cal BC (table S1)

Grave No. 5: KKBR5 (KKN085)
Grave No. 5 consists of postcranial remains along with a partial mandible of an adult who was 40+ years at the time of death (KKN085 this study). The entire cranium is missing (although several teeth were present), and several postcranial elements show signs of damage and breakage from post-mortem activity. The grave was looted and along with the cranium missing, the capstone was broken. Looting presumably took place after advanced skeletonization of the body. Skeletal elements were recovered in a jumbled state at the extreme western end of the cist. The above ground stone fence is semi-rectangular and encloses an area of 5 sq. meters. No grave goods were recovered.

- Pathologies: None observed
- Osteological sex: Male
- Genetic sex: Male
- Radiocarbon date: None

Grave No. 8: KKBR8 (KKN086)
Grave No. 8 contains a nearly complete postcranial skeleton and mandible (KKN086 this study). KKN086 is estimated to be a middle-aged adult aged between 30-45 at time of death. The individual had a large amount of dental calculus on the mandibular teeth. In addition, the joint surfaces on bones showed extensive micro- and macroporosity. The burial was disturbed and the neurocranium missing. Partial remains of a semi-rectangular fence enclosing an area of 3 sq. meters were recovered in situ, and some stones were also recovered that had been dumped into the cist itself. The intrusion of groundwater into the cist disturbed the layout of internal contents. The base of the grave contained several fragments from the rim of a medium ceramic jar (горшок) (rim diameter 11.5 cm) with incised decoration along the neck. The design consists of a band of upright oblique triangles followed by a row of inverted oblique triangles with internal hatching. Three small bronze disk-beads, 4 mm in diameter, were also recovered.

- Pathologies: This individual had a large amount of dental calculus on the mandibular teeth. In addition, the joint surfaces on bones are covered in extensive micro- and macroporosity.
- Osteological sex: Not determined. Both os coxa of this individual displayed mixed male and female typical traits.
- Genetic sex: Female
- Radiocarbon date: 1767-1623 cal BC (table S1)

Grave No. 9: KKBR9 (KKN087)
Grave No. 9 contained nearly complete cranial and post-cranial remains of a sub-adult aged 14-18 at the time of death (KKN087). The cranium, mandible, and all upper and lower limb bones were present and complete. However, the burial was disturbed and the grave contents were recovered in a jumbled state mostly at the western end of the cist. The stone architecture was also recovered largely out of context, but the shape of the fence retained its original square form. A medium ceramic jar (горшок) was recovered from the NW corner of the cist in its original position. The jar measures 15 cm high and has a rim diameter of 15 cm and base diameter of 9.5 cm. An incised pattern depicting four consecutive rows of zig-zagging adorn the neck. Along

with the jar, the burial fill contained three fiance beads (approx. 3 mm diameter 4-6 mm length) and two gold tubular beads (approx. 3 mm diameter 12mm length).

- Pathologies: The thoracic vertebrae show extensive superior-inferior compression and also exhibit micro- and macroporosity on their anterior aspect.
- Osteological sex: Not determined
- Genetic sex: Female
- Radiocarbon date: 1616-1454 cal BC (table S1)

Grave No. 13: KKBR13 (KKN090 and KKN091)
Grave No. 13 contained a complete skeleton of a middle-aged adult (KKN090) that was recovered undisturbed despite an attempted looting even in the past (fig. S3). Grave No. 13 also held the cranium of a second individual (KKN091, later determined to belong to KKN089 in KKBR12) that was secondarily deposited into the upper sediments of the cist during the unsuccessful looting, along with a long stone pillar that was rammed into the base of the grave and left protruding from the top (fig. S3B). The primary individual buried in grave No. 13 lay in a flexed position on the left side with head oriented west. At the head of the burial were two large pottery jars (горшоки) with intricate stamped and incised patterns from the rim to the base. One vessel measures 22 cm high and has a rim diameter of 22 cm, and base diameter of 11 cm. The second vessel measures 18.5 cm high with a rim diameter of 20cm and base diameter of 10 cm. The deceased wore bronze hoop earrings on each side of the head.

- Pathologies: None identified
- Osteological sex: Possible male
- Genetic sex: Female
- Radiocarbon date: 1616-1454 cal BC (table S1)

Grave No. 16: KKBR16 (KKN096)
Grave No. 16 contained a very incomplete skeleton of an adult aged 20-40 years (KKN096). The grave was disturbed, and a few long bones, vertebrae, pelvis and a partial mandible were recovered in a jumbled state both inside the cist and at its opening in the area between the cist and outer fence. The outer stone fence was largely dismantled, removing any sign of its original form. Some grave goods were left, including a faience bead and a medium size ceramic jar (горшок-банка) measuring 16.5 cm high, with a diameter of 16 cm at the rim, and 10 cm at the base. It was decorated with three concentric raised coils around the neck zone.

- Pathologies: Left facies lunata widened to ventral. Strong muscle attachments on left tuberositas deltoidea.
- Osteological sex: Male
- Genetic sex: Male
- Radiocarbon date: None

Grave No. 22: KKBR22 (KKN102)
Grave No. 22 contained few skeletal elements, consisting of a fragmentary cranium and postcranial skeleton of an adult aged between 30-40 (KKN102). No grave goods were recovered.

- Pathology: Pacchionian granulation; cranium thickened; lambdoid ossicles.
- Osteological sex: Possible male
- Genetic sex: Male
- Radiocarbon date: None

1.2.2 Double Graves

Grave No. 14.1 and 14.2: KKBR14 (KKN092 and KKN093)

Grave No. 14 consists of one large oval stone fence enclosing an area of 17.5 sq. meters and two cists placed adjacent containing children. The children were granted a burial no less elaborate than the adults found in the graveyard. Burial-1 contained a three-year-old child (KKN092), who was placed on the left in a flexed position, head oriented west. Behind the head at the western end of the cist was a medium ceramic jar (горшок) with rounded shoulders and a comb-stamped vertical herringbone design on the zone of the neck. The jar measures 16 cm high, and has a rim diameter of 17 cm and base diameter of 10 cm. A comparable vessel was found at the Talsinsky Burial Site-1 in central Kazakhstan (*102*). In addition, a highly corroded bronze object was recovered, as well as animal bones from a medium-sized mammal. Burial-2 held a child of a similar age (KKN093), but this burial had been disturbed and the bones displaced from their original position. A small bone pendant with a drilled hole was recovered, as well as small fragments from two different comb-stamped ceramic containers.

- Pathologies: KKN092 - none identified. KKN093 - lambdoid ossicles.
- Osteological sex: Not determined
- Genetic sex: KKN092 - female. KKN093 - male
- Radiocarbon date: None

Grave No. 15.1 and 15.2: KKBR15 (KKN094 and KKN095)

Grave No. 15 consists of one large oval stone fence enclosing an area of 17.5 sq. meters and two burial cists oriented approximately W-E (fig. S4). Burial-1 is a cist containing an adult aged 20-30 years (KKN094 this study). KKN094 is a genetic outlier in this study, and because this burial was found in an undisturbed state it is possible to make a complete report of the grave and its contents. The individual is genetically female, and was laid on the left side in a flexed position, with the head oriented to the west. The individual wore a bronze beaded bracelet around each ankle. Other articles included a bronze hoop with a carnelian bead threaded onto it that was positioned in front of the face (possibly a nose ring), two bronze hoops (one lying beneath the chin region and the other close to the hip bone), and a faience bead. A small black ceramic pot (банка) measuring 11 cm high, with a rim diameter of 11 cm and base diameter of 8 cm lay in the NW corner of the cist behind the individual’s head. An incised ornament consisting of three concentric lines adorns its neck. The pot was capped with a stone ax that served as a lid. While Burial-1 was unlooted, the neighboring Burial-2 was disturbed. Burial No. 1 may have gone unnoticed because the cist was set considerably deeper into the ground than that of the neighboring Burial-2, which hid it from easy view. Burial-2 held a nearly complete postcranial skeleton of a 30-45 year-old adult (KKN095) who had been laid on their left side in a flexed position, oriented west. Some skeletal elements were missing, including the cranium, mandible, and some arm long bones. However, bones of the hands remained in situ so the robbery may have taken place after advanced skeletonization of the body. Individual KKN095 was buried along with a small ceramic pinch pot (height 7 cm; rim diameter 8 cm; base diameter 4.5 cm)

that was fired at a low temperature resulting in a brittle fabric. The pinch pot (банка) sat at the western end of the cist.

- Pathologies: KKN094 - Lambdoid ossicles. Wisdom teeth (M3s) missing congenitally. Dental calculus accompanied by periodontitis in both the maxillary and mandibular dentition, especially along the premolars and molars. Anterior teeth show advanced wear, possibly reflecting usage as a “3rd hand”. Degeneration of spine or hip. Slight osteophytes on proximal and distal surfaces of lumbar vertebra. KKN095 - Osteophytes on left facies lunata, facies widened ventral. Femora slightly bent at tuberositas glutea, possibly due to overuse of gluteal muscles. Osteophytes on pars basilaris sacralis and surfaces of lumbar vertebrae. Linear enamel hypoplasias. Anterior teeth are highly worn, possibly due to an underbite. Medial and distal phalanges of 2nd finger fused from healed trauma.
- Osteological sex: KKN094 - female. KKN095 - possibly male.
- Genetic sex: KKN094 - female. KKN095 - female.
- Radiocarbon date: KKN094, 1743-1620 cal BC (table S1)

1.2.3 Triple Grave Cluster

Graves No. 10, No. 11, and No. 12 belong to a discrete group of large burials at the northern end of the graveyard (fig. S5). The three burials are joined by intersecting circular stone fences enclosing an area of 20 sq. meters each. A different style of grave is present whereby the subterranean cists are fashioned from smaller tile-shaped stones stacked like brickwork and sealed with a stone cap. This style of burial is documented among Fedorovka Culture burial grounds in eastern Kazakhstan, such as at Ust-Bykon and Sarykol-2 (*49*), and among Fedorovka Culture (Nurinsky type) graves in central Kazakhstan at sites such as Darinski (*42*) and Korzhar (*103*). The cists lack consistent orientation. KKBR10 and KKBR12 are inhumations, but KKBR11 contained a cremation so it was omitted from this study. The group of burials were all opened and disturbed during antiquity.

Grave No. 10: KKBR10 (KKN088)

Grave No. 10 contained nearly complete cranial and postcranial remains of an adult (KKN088). The grave was disturbed and the skeletal elements recovered in a jumbled state in the north west corner of the burial cist. A miniature shaft-hole axe bronze pendant measuring 3.5 cm in length was recovered from the floor of the cist. Such items are found in other Fedorovka Culture burial grounds of northern Eurasia and represent replica versions of Andronovo Culture shaft hole axes present throughout the Altai region (*104*). The shaft hole was sufficient in width to allow a cord to be threaded through it.

- Pathologies: None identified
- Osteological sex: Not determined. The cranial and os coxa morphology show mixed male and female or indeterminate traits.
- Genetic sex: Female
- Radiocarbon date: 1616-1454 cal BC (table S1)

Grave No. 12: KKBR12 (KKN089)

Grave No. 12 contained most elements of the postcranial skeleton of a middle-aged adult (KKN089) recovered in a jumbled state. The distribution of the skeletal elements suggests that the tomb was looted while some connective tissues were still partially intact. Neither the mandible nor the cranium were recovered in Grave No. 12. However, during the course of this study, the cranium (KKN091) was identified in the adjacent Grave No. 13, where it presumably ended up during the course of the looting event. No grave goods were found.

- Pathologies: None identified
- Osteological sex: Not determined
- Genetic sex: Female
- Radiocarbon date: None

1.2.4 Ring-Burial Group
A group of five burials form a discrete group at the southern end of the graveyard (Grave No. 17, Grave No. 18, Grave No. 19, Grave No. 20, and Grave No. 21) (fig. S6). The five graves are each ringed by their own circular stone fence enclosing an area of just 4 sq. meters each, and their upper surface architecture either overlaps or are within close proximity of each other. The central stone cists are oriented NE-SW and are comparatively shallow compared to the other graves in the graveyard. All of the ring burials, except for one, were recovered in a severely disturbed state.

Grave No. 17: KKBR17 (KKN097)
Grave No. 17 contained a few skeletal elements of an adult aged 60+ years (KKN097). The bones were jumbled in the cist, and it was not possible to reconstruct the original body position.

- Pathologies: Visible sutura metopica. Pacchioni granulation on the inside of the cranium.
- Osteological sex: Male
- Genetic sex: Female
- Radiocarbon date: None

Grave No. 18: KKBR18 (KKN098)
Grave No. 18 contained a few skeletal elements of a young adult (KKN098). It was not possible to reconstruct the original body position due to the small amount of skeletal material recovered. Sediments within the cist yielded a single gold tubular bead identical in appearance to the gold tubular bead (d. 3 mm x l. 12 mm) buried with individual KKN098 in Grave No. 9.

- Pathologies: Degeneration of spine or hip. Surfaces of thoracic vertebrae slightly widened to right, very slight beginning of osteophytes.
- Osteological sex: Possible male
- Genetic sex: Female
- Radiocarbon date: None

Grave No. 19: KKBR19 (KKN099)
Grave No. 19 contained the partial cranial and postcranial remains of an adult aged 50-70 years (KKN099). KKN099 was identified as a genetic outlier in this study. It was not possible to

reconstruct the original body position as the bones were recovered in a jumbled state. No grave goods were recovered.

- Pathologies: Lambdoid ossicles. Intravital tooth loss, dental caries and periodontitis in the maxillary and mandibular dentition. Degeneration of spine or hip. Acetabulum, left widened to ventral. Slight osteophytes on femoral heads. Osteophytes on lumbar vertebrae. One compressed vertebral disc plate. Lambdoid ossicles along the temporal bone. Femora slightly bent outwards on tuberositas deltoidea, possibly due to muscle pulling. Pronounced enthesis on right humerus.
- Osteological sex: Male
- Genetic sex: Female
- Radiocarbon date: 1875-1637 cal. BC (table S1)

Grave No. 20: KKBR20 (KKN100)
Grave No. 20 contained skeletal elements of a child aged 1-5 years (KKN100). The grave lacked a stone cist. Instead, the child was buried in a small earthen pit lined at the opening by a ring of stones. The original body position was unclear due to looting of the grave. No grave goods were recovered.

- Pathologies: None detected
- Osteological sex: Not determined
- Genetic sex: Male
- Radiocarbon date: None

Grave No. 21: KKBR21 (KKN101)
Grave No. 21 contained the remains of two individuals. Individual 1 (KKN010) was placed on the left side in a flexed position, oriented southwest. Part of the postcranial skeleton and teeth were recovered. The cranium was absent. The individual was an adult aged 30+ years. Individual 2 was represented by only a hip bone fragment and was omitted from this study. A ceramic pot (банка) measuring 13 cm high with a rim diameter of 13 cm and base diameter of 8 cm was recovered at the foot of the cist. The neck of the pot was decorated with a comb-stamped band of four concentric zigzags.

- Pathologies: Os acromiale on right scapula. Degeneration of spine or hip. Possible pseudo joint is found in the condyles lateralis of the left tibia. Vertebral rib endings worn down, signs of inflammation. Osteophytes on thoracic vertebrae. Osteophytes on femoral condyles. Osteophytes on condylus lateralis tibiae. Condylus lateralis with polished area on dorsal part, probable pseudo joint. Lip building on the right collum humeri, joint widened to dorsal, signs of inflammation. Lumbar vertebrae 4 and 5 fused, strong osteophytes and inflammation discreta. Inflammation on sacrum. Granulation on second lumbar vertebra.
- Osteological sex: Possible male
- Genetic sex: Female
- Radiocarbon date: None

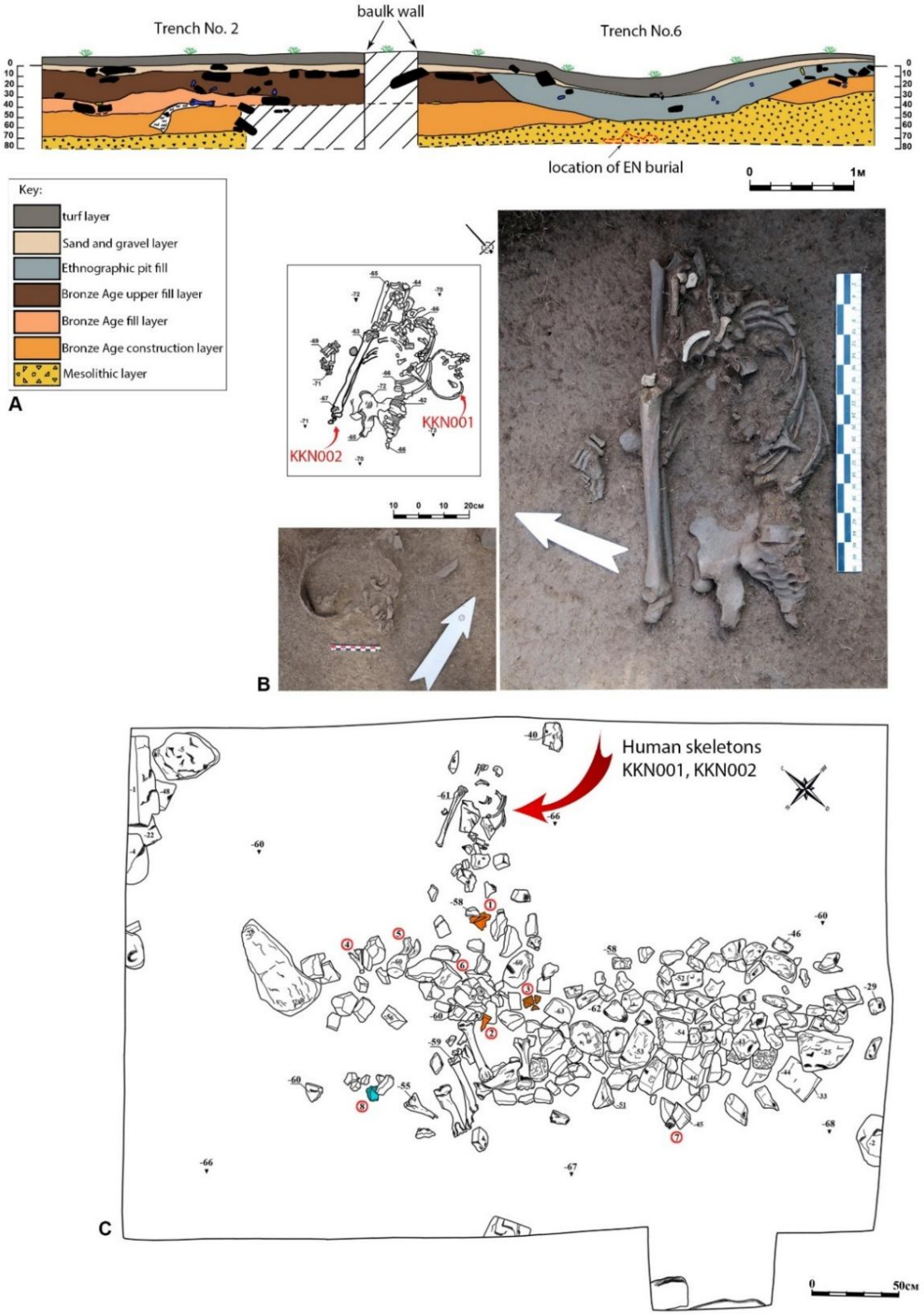

Fig. S1. Early Neolithic burial at Koken. (**A**) Stratigraphic profile showing location and depth of the EN burial. (**B**) Excavation photographs showing KKN001 (cranium) and KKN002 (postcranial remains); arrows point north. Illustration shows the relative in situ position of the skeletal elements of the two individuals, including depths in cm. (**C**) Overhead illustration of the EN burial in trench No. 6, showing associated faunal remains and other features (Photo Credit: Zhuldyz Tashmanbetova, Washington University in St. Louis; Artist Credit: Galymzhan Kiyasbek, Margulan Institute of Archaeology).

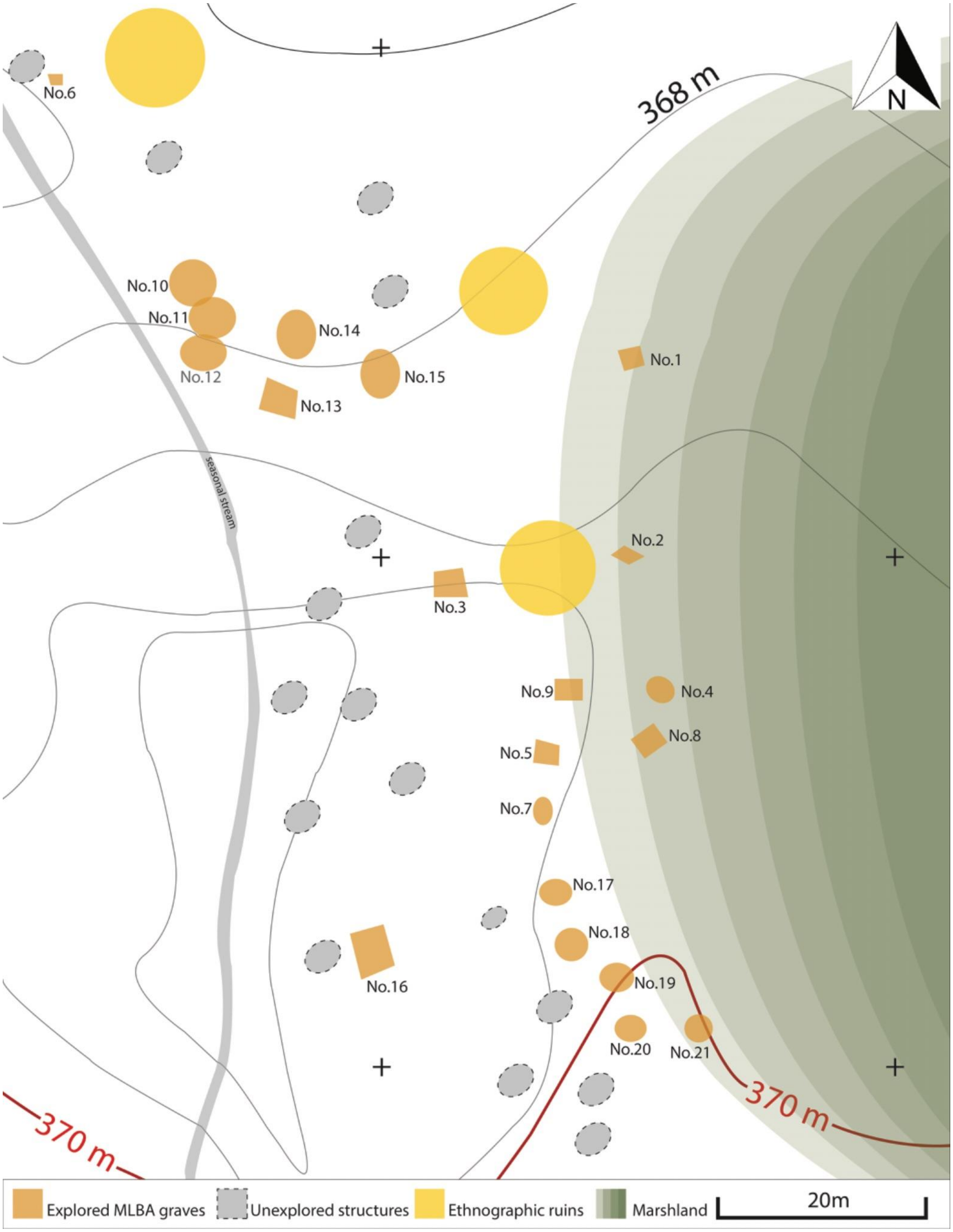

Fig. S2. Middle-Late Bronze Age Koken-3 Graveyard. A total of 22 graves were excavated (orange rectangles and ovals), while 16 additional structures were recorded but not excavated (gray). Of the excavated graves, 17 were included in this study (data S1).

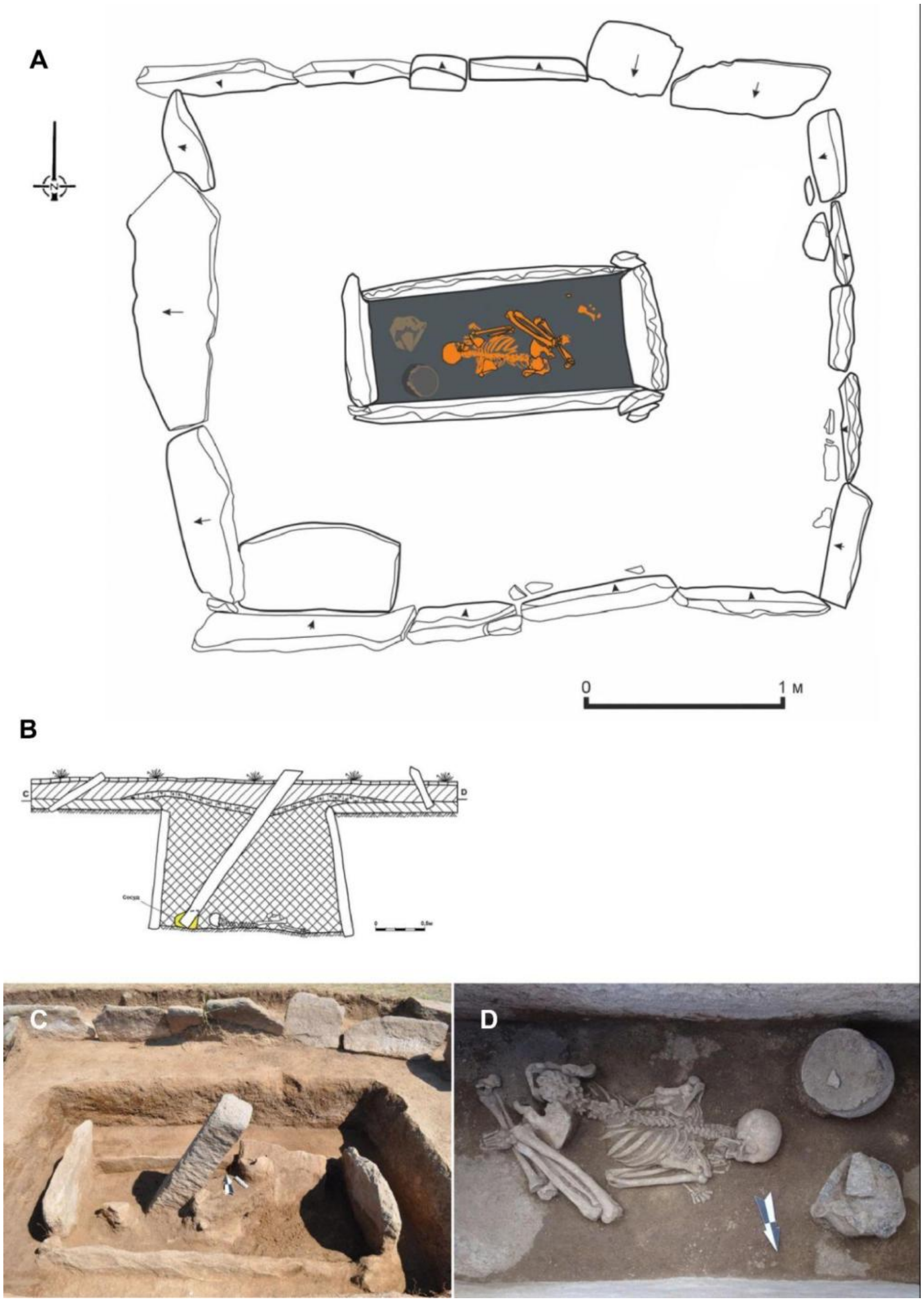

Fig. S3. Koken MLBA Graveyard, example of a single grave: KKBR13. (**A**) Overhead illustration of KKBR13 showing an undisturbed inhumation (KKN090) with grave goods in a central rectangular stone cist surrounded by a rectangular stone fence (arrows signify the tilt of the stones). (**B**) Profile view of KKBR13. Note the presence of a large stone pillar that was rammed in the grave during looting (**C**) Overhead excavation photograph of KKBR13. Note the presence of the intrusive cranium (KKN091) located alongside the stone pillar at the grave opening, (**D**) Excavation photo showing KKN090, buried in a flexed supine position, with grave goods; arrow points north (Photo Credit: Paula Doumani Dupuy, Nazarbayev University; Artist Credit: Erbolat Rakhmankulov, State Historical and Cultural Museum-Reserve "Bozok").

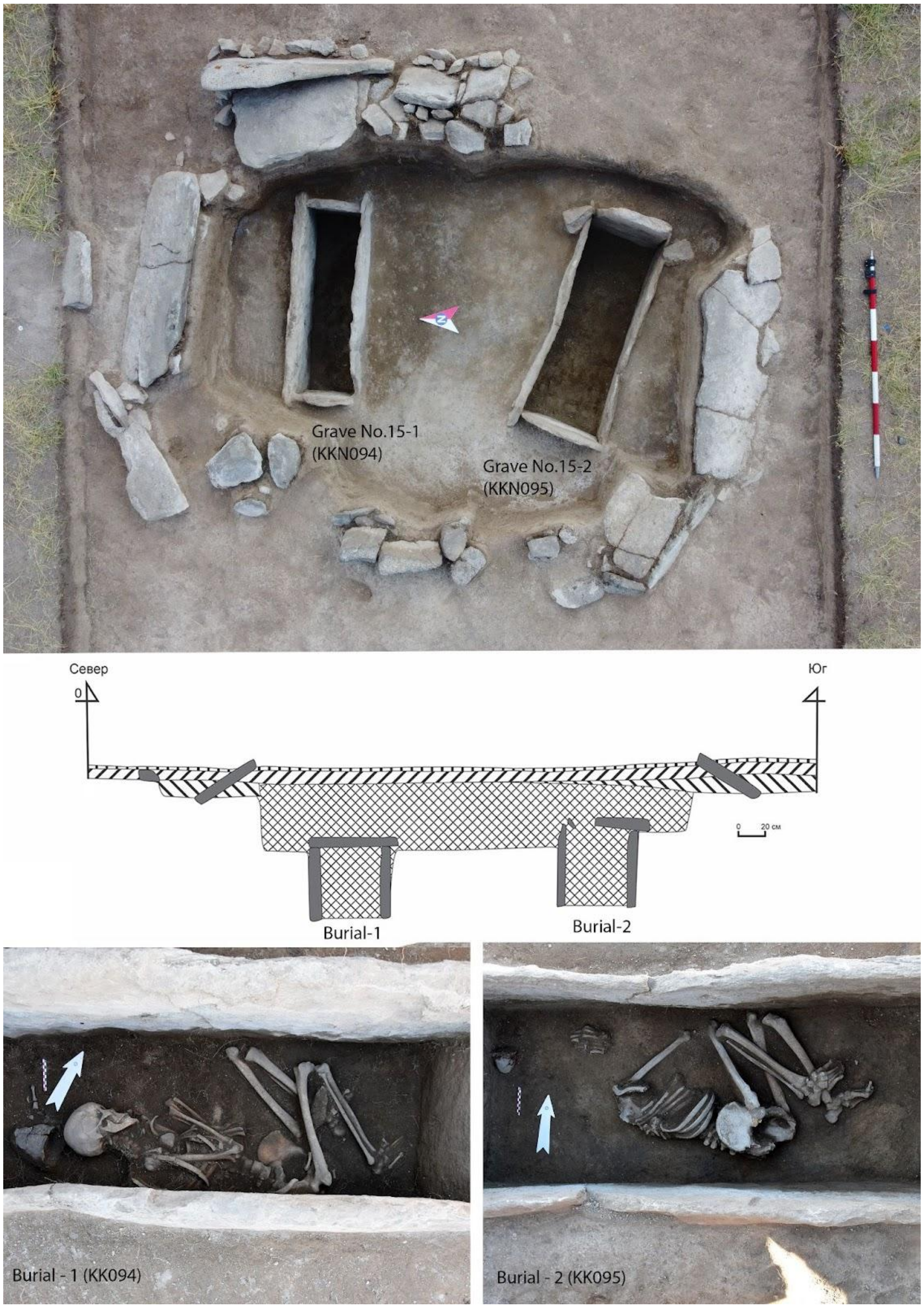

Fig. S4. Koken MLBA Graveyard, example of a double grave: KKBR15. (**A**) Overhead photograph of the double grave KKBR15 showing two rectangular stone cists surrounded by an oval stone fence. (**B**) Profile illustration of the two graves. Note the deeper placement of Burial-1 compared to Burial-2. (**C**) Excavation photograph of KKBR15 burial 1 showing the undisturbed remains of KKN094. (**D**) Excavation photograph of KKBR15 burial 2 showing the disturbed remains of KKN095. Arrows point north (Photo Credit: Erbolat Rakhmankulov, State Historical and Cultural Museum-Reserve “Bozok”; Artist Credit: Erbolat Rakhmankulov, State Historical and Cultural Museum-Reserve “Bozok”).

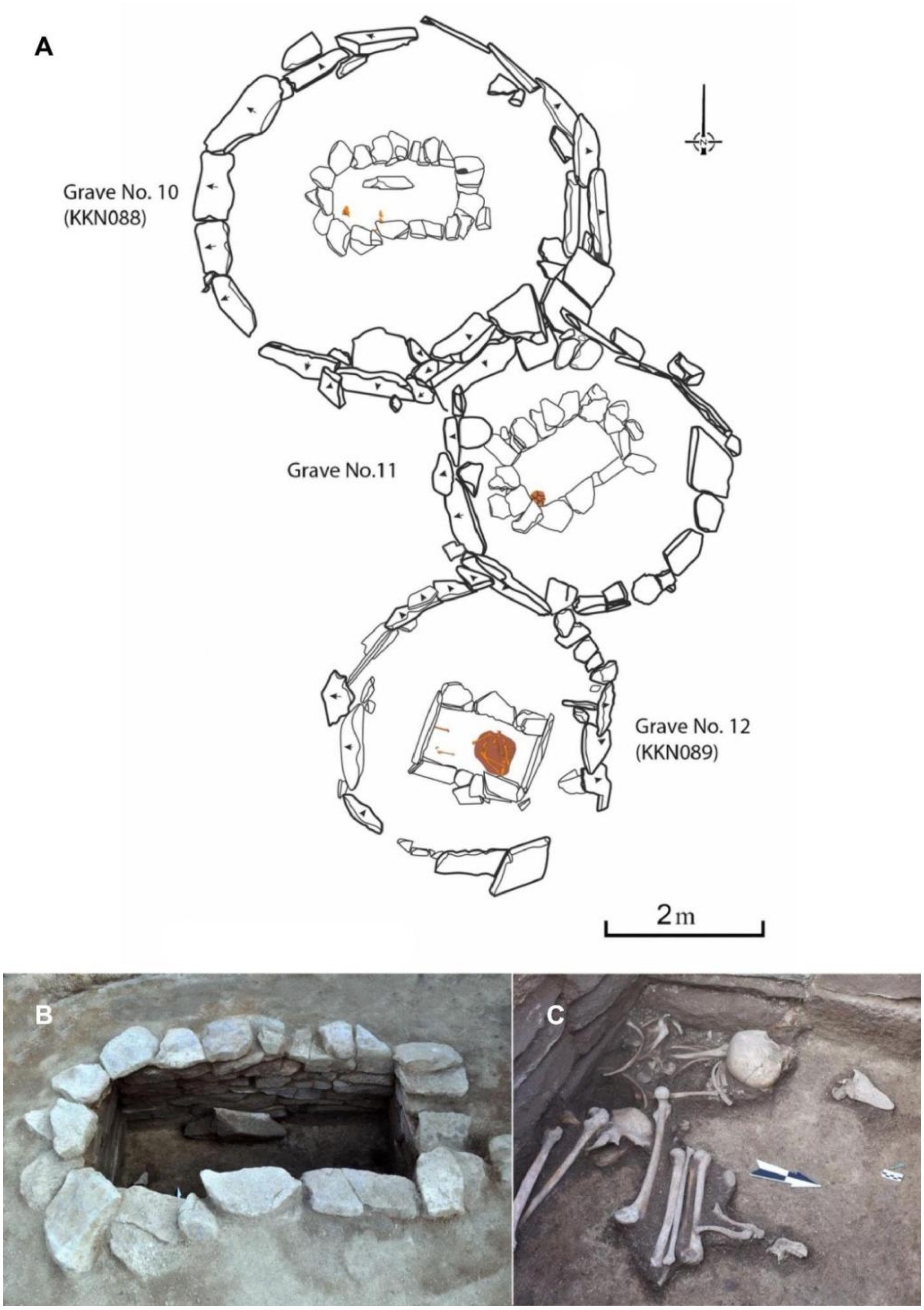

Fig. S5. Koken MLBA Graveyard, example of a triple grave cluster: KKBR10-12. (**A**) Overhead illustration showing the graves KKBR10 (KKN088), KKBR11 (not included in study), and KKBR12 (KKN089), each consisting of a central rectangular stone cist surrounded by a circular stone fence (arrows signify tilt direction of stones). (**B**) Excavation photo of grave KKBR10 showing subterranean cist fashioned from stones stacked like brickwork. (**C**) Excavation photo of grave KKBR10 containing the disturbed remains of KKN088. Arrow points north (Photo Credit: Aidyn Zhuniskhanov, Nazarbayev University; Artist Credit: Erbolat Rakhmankulov, State Historical and Cultural Museum-Reserve "Bozok").

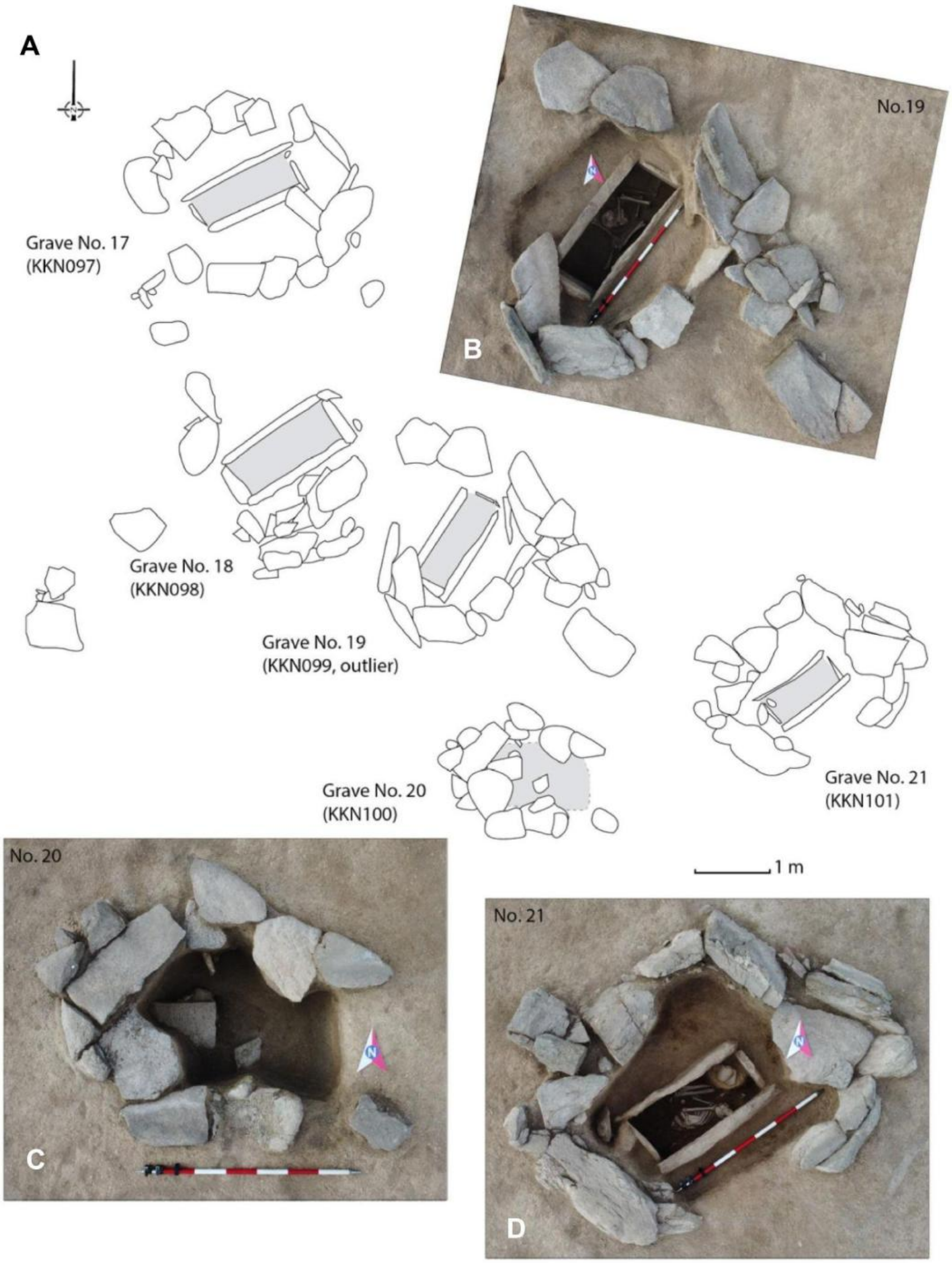

Fig. S6. Koken MLBA Graveyard, example of a 5-grave ring burial group: KKBR17-21. (**A**) Overhead illustration showing the graves KKBR17 (KKN097), KKBR18 (KKN088), KKBR19 (KKN099), KKBR20 (KKN100), and KKBR21 (KKN101) arranged into a ring burial group. Each grave (except KKRB20) consists of a central rectangular stone cist surrounded by stone fence. (**B**) Overhead excavation photograph of KBR019 showing the disturbed remains of KKN099. (**C**) Overhead excavation photograph of KKBR20. (**D**) Overhead excavation photograph of KKBR21 showing the disturbed remains of KKN101 (Photo Credit: Erbolat Rakhmankulov, State Historical and Cultural Museum-Reserve “Bozok”; Artist Credit: Paula Doumani Dupuy, Nazarbayev University).

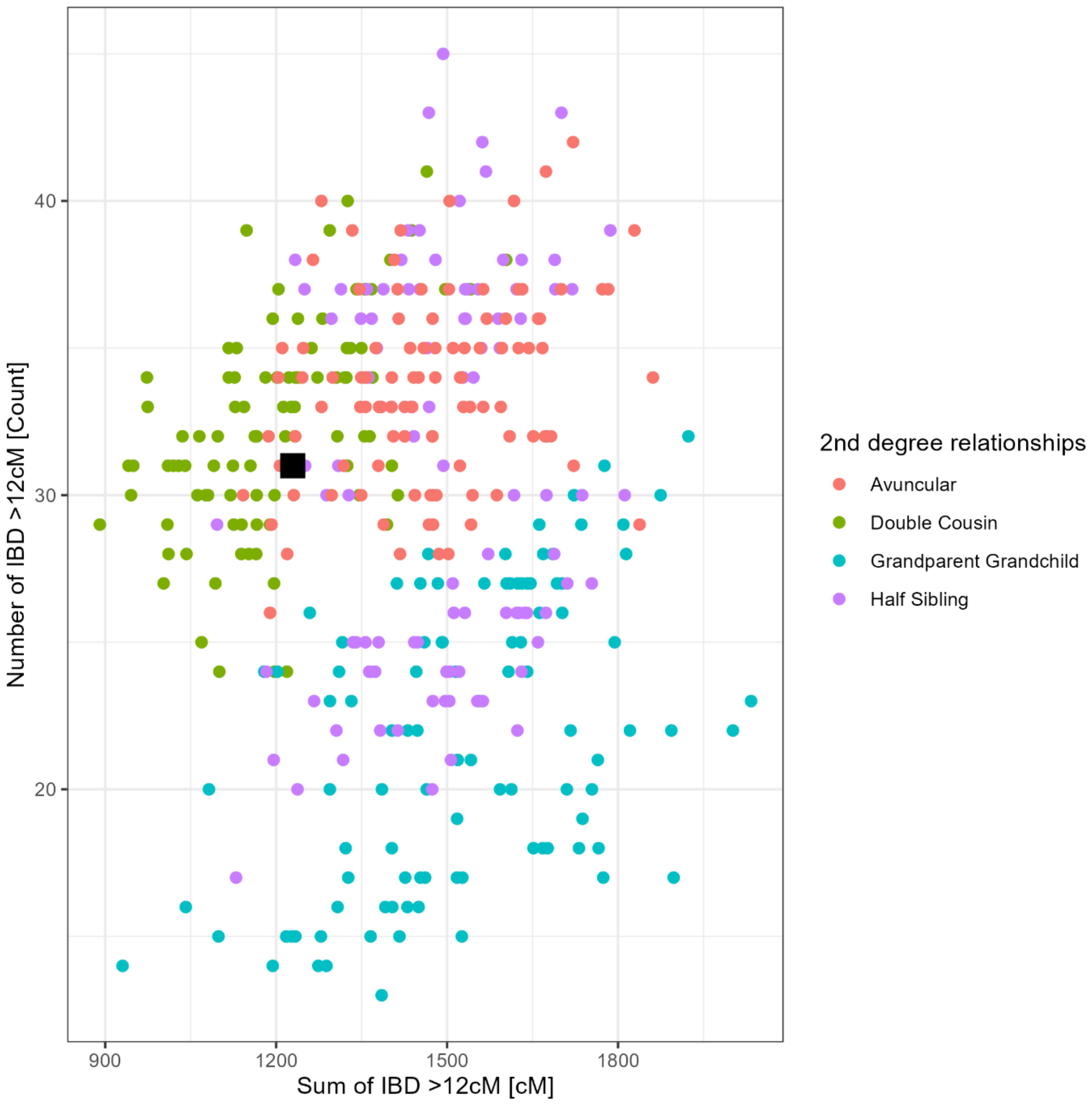

Fig. S7. Comparison of IBD segment sharing between EN Koken individuals with different second-degree relationships. We plot the total length of IBD segments with >12 cM length (x-axis) against the number of such segments (y-axis) for the EN Koken pair (KKN001 and KKN002, black rectangle). We compared this value with values from simulated data with different second-degree pairs: avuncular (pale red), double first cousin (dark green), grandfather-grandson (pale blue), and half siblings (purple). 100 simulated data were produced for each type of the second degree relationship. Koken data do not overlap with the simulated range for grandfather-grandson, while overlap with the other three relationships.

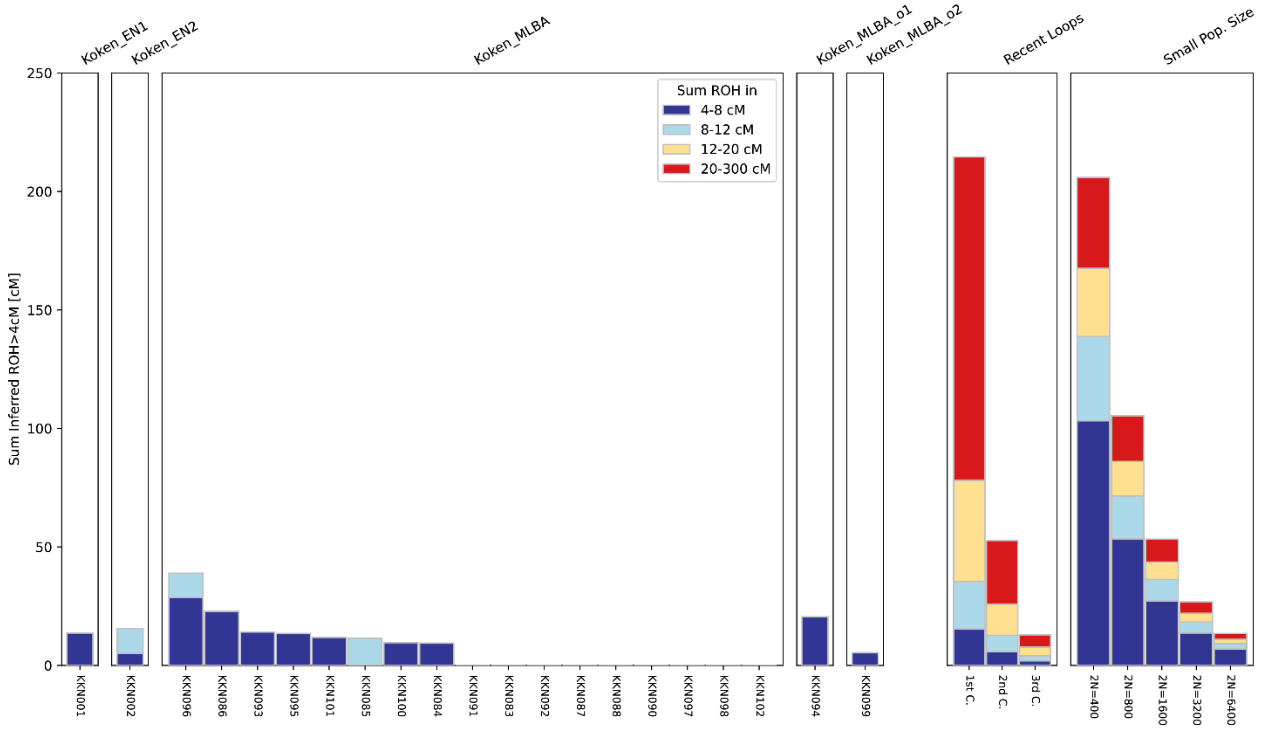

Fig. S8. Runs of Homozygosity blocks in the ancient Koken individuals. The total length of ROH blocks present in the genomes of EN and MLBA Koken individuals were counted by each length category: 4-8, 8-12, 12-20, and 20-300 cM. Lack of long (≧ 12 cM) ROH blocks in both EN and MLBA Koken individuals suggest no evidence for recent consanguineous pairing in their ancestors.

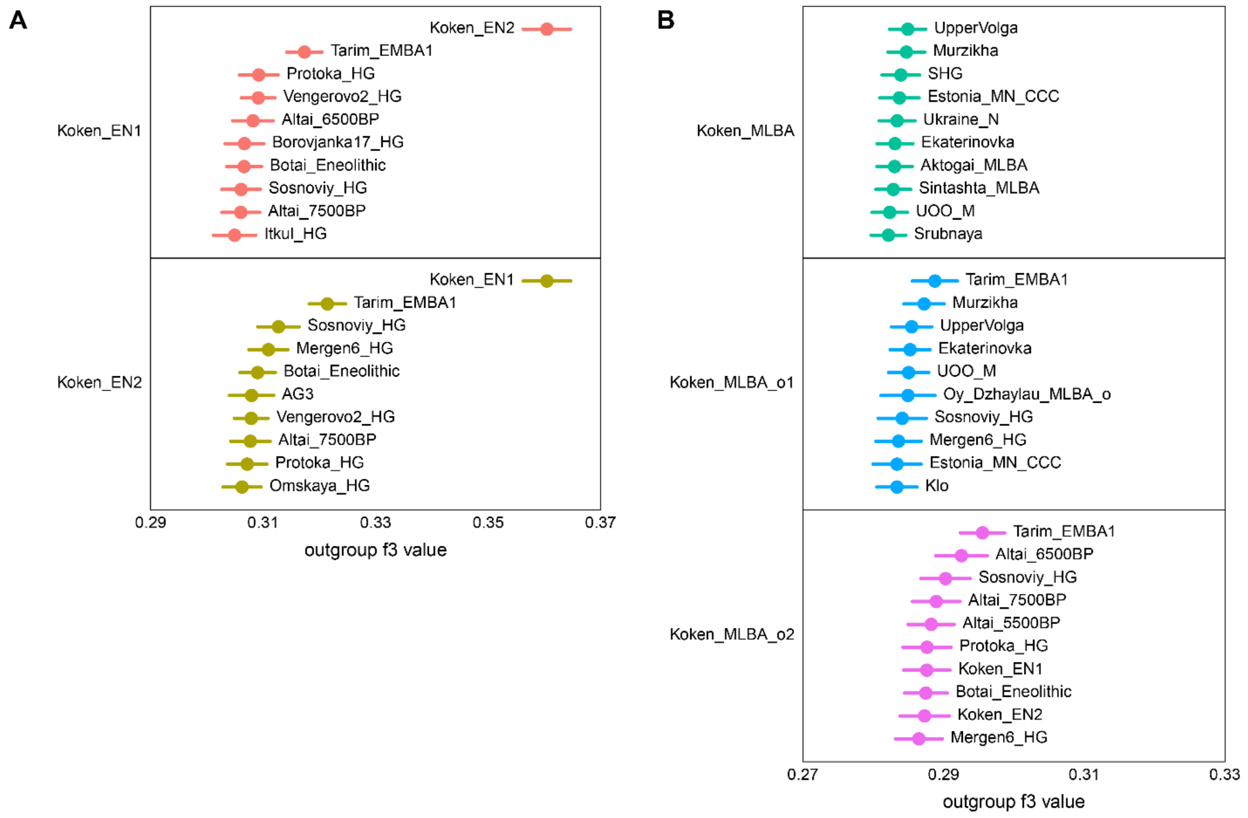

Fig. S9. Outgroup-f3 values for the Early Neolithic (EN) and Middle-Late Bronze Age (MLBA) Koken individuals. Outgroup-f3 statistics are calculated in the form of f3(Mbuti; X, Target) for world-wide ancient and present-day populations "X" (data S3), and the top 10 populations for each target are shown: (A) Koken EN groups and (B) Koken MLBA groups. The color-filled circles and their associated horizontal bars represent the point estimate and ±1 s.e.m. calculated by 5cM block jackknifing. Outgroup-f3 values for all X's are reported in data S5.

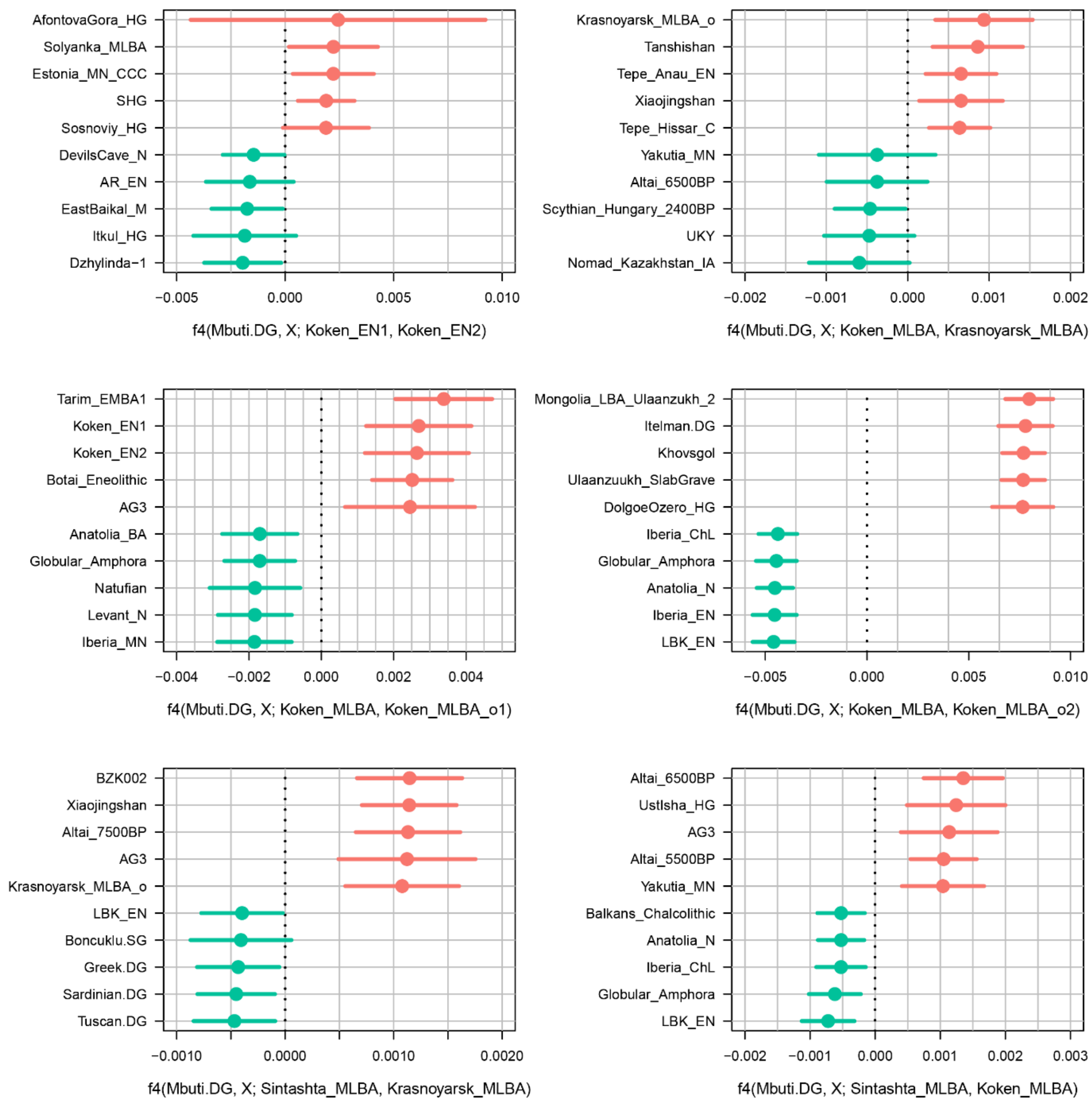

Fig. S10. Genetic symmetry test for pairs of target populations using f_4 statistic. For each of the six pairs of target populations, we calculated f4(Mbuti, X; target 1, target 2) for a set of world-wide ancient and present-day populations "X" (data S3). We present the 5 most positive and 5 most negative f_4 statistics are shown in red and blue, respectively. Significant positive values represent that X is closer to target 2 compared to target 1, and the negative values represent the opposite situation. The color-filled circles and their associated horizontal bars represent the point estimate and ±3 s.e.m. calculated by 5cM block jackknifing. F_4 values for all X's are reported in data S6.

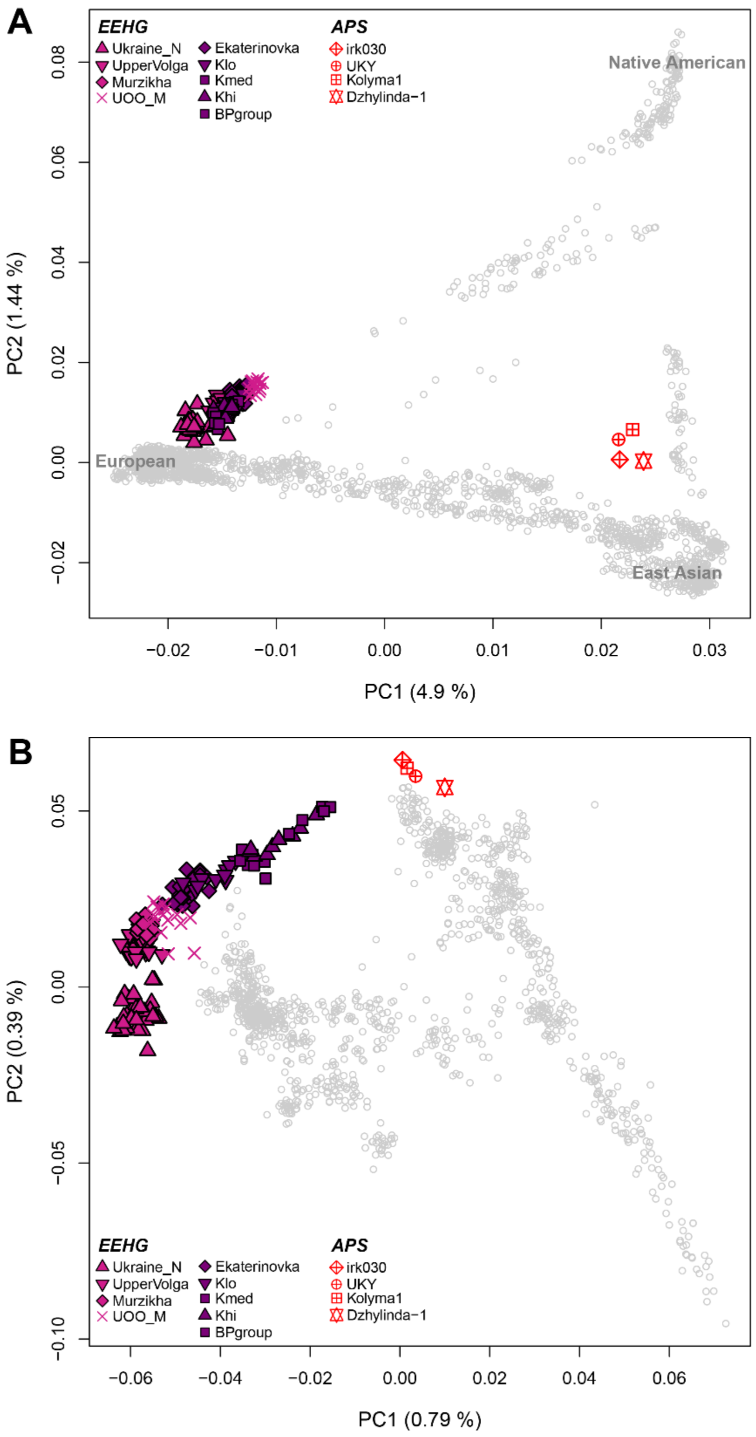

Fig. S11. Principal component analysis of EEHG and APS source populations used in modeling Siberian forest-steppe hunter-gatherers. (**A**) Top two principal components (PCs) calculated from 2,270 present-day Eurasian and American individuals. (**B**) Top two PCs calculated from 1,238 present-day western Eurasian individuals. Both in (**A, B**), grey circles mark present-day individuals used for calculating PCs. Ancient individuals, marked by color-filled symbols, were projected onto the calculated PCs. Candidate populations for EEHG ancestry are shown in orange-related colors, while those for APS ancestry are shown in purple.

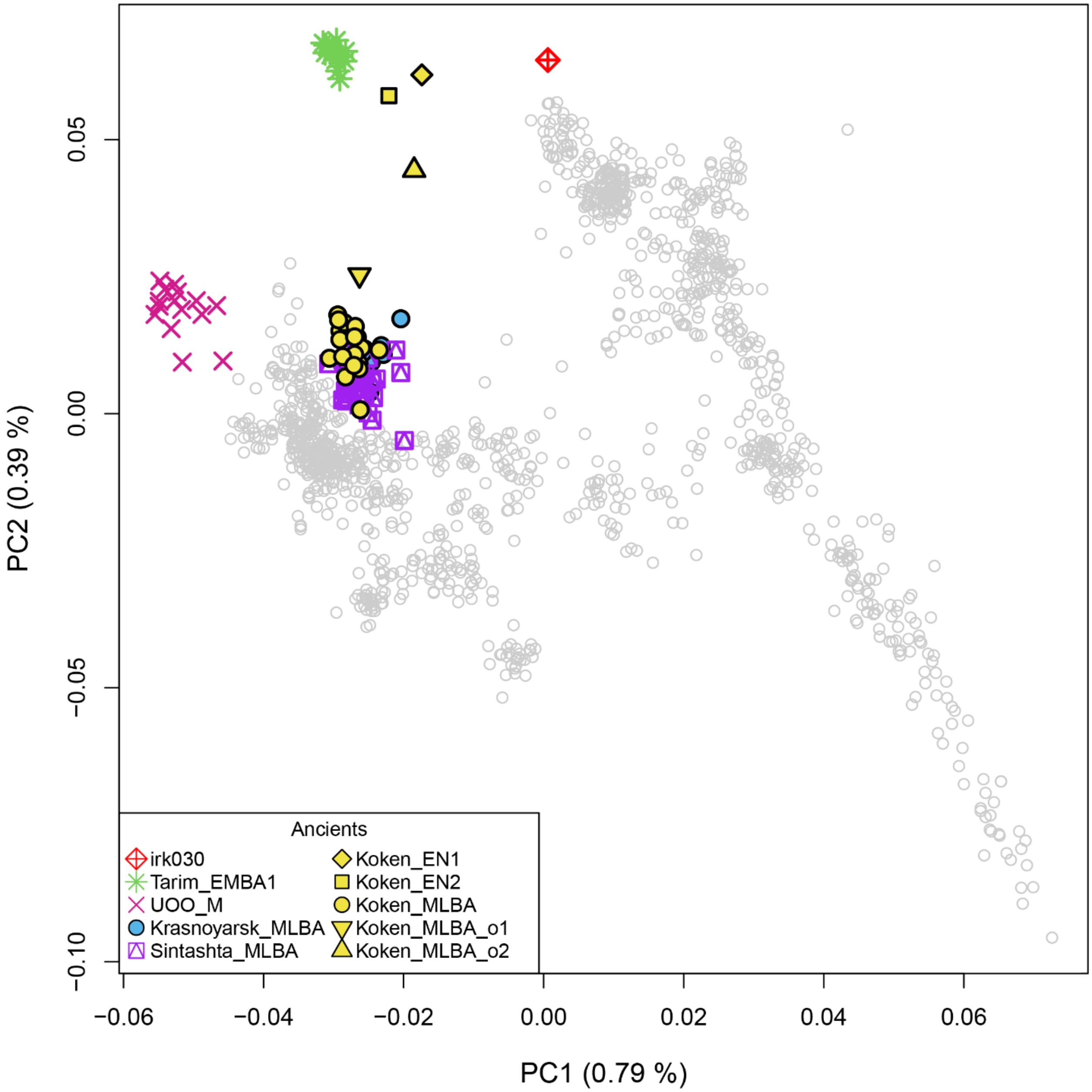

Fig. S12. Principal component analysis performed with present-day western Eurasian individuals. This plot shows the top two PCs calculated from 1,238 present-day western Eurasian individuals. Grey circles mark present-day individuals used for calculating PCs. Ancient individuals, marked by color-filled symbols, were projected onto the calculated PCs.

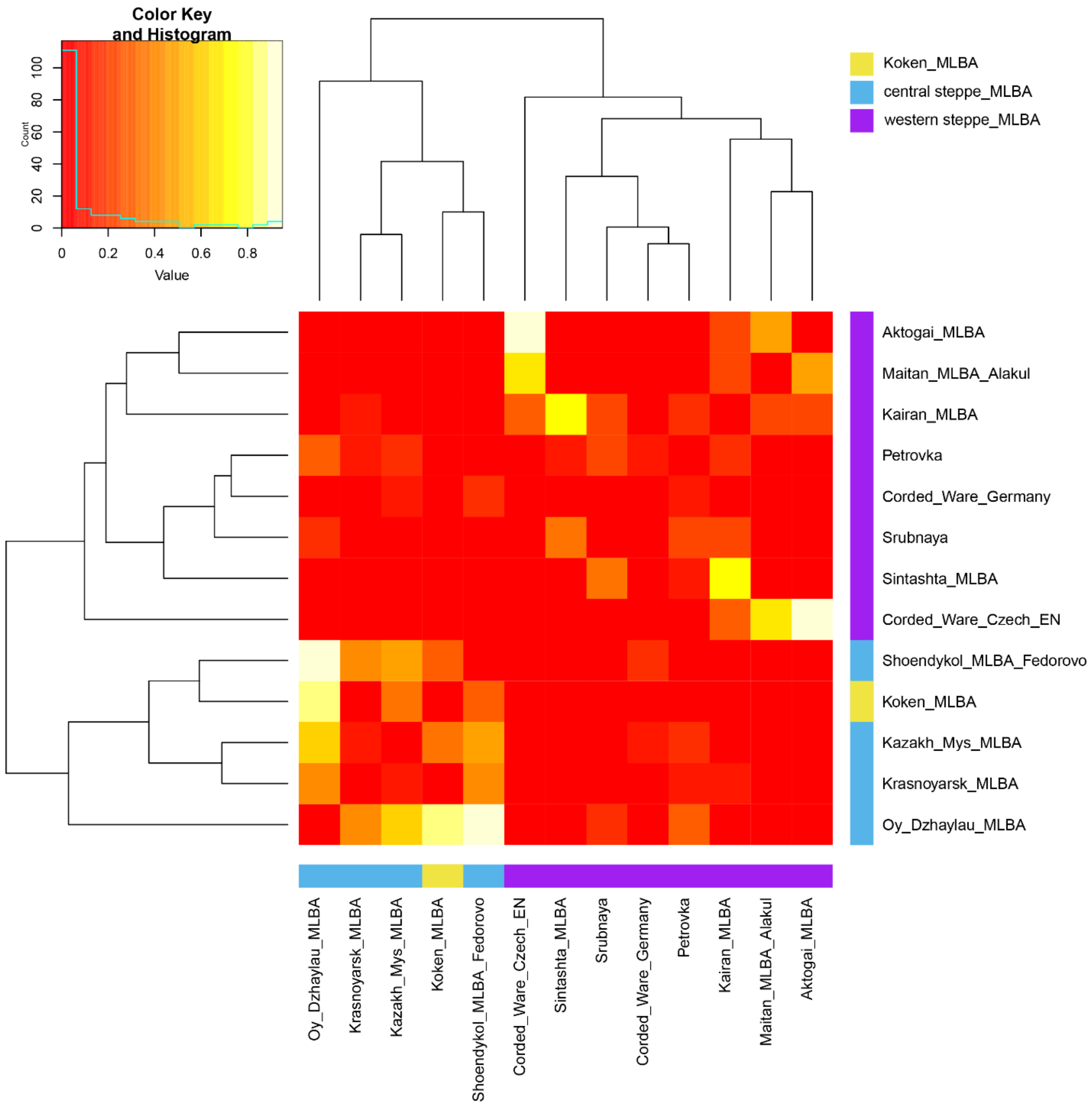

Fig. S13. Heatmap of qpWave p-values comparing Koken_MLBA, central steppe_MLBA, and western steppe_MLBA populations. We estimated qpWave p-values for all pairwise comparisons among populations with at least three individuals from Koken_MLBA, central steppe_MLBA (n=4), and western steppe_MLBA (n=8). Koken_MLBA consistently exhibits higher p-values when compared with central steppe_MLBA than with western steppe_MLBA, resulting in its clustering alongside central steppe_MLBA populations. Populations are color-coded as follows: Koken_MLBA in yellow, central steppe_MLBA in blue, and western steppe_MLBA in green.

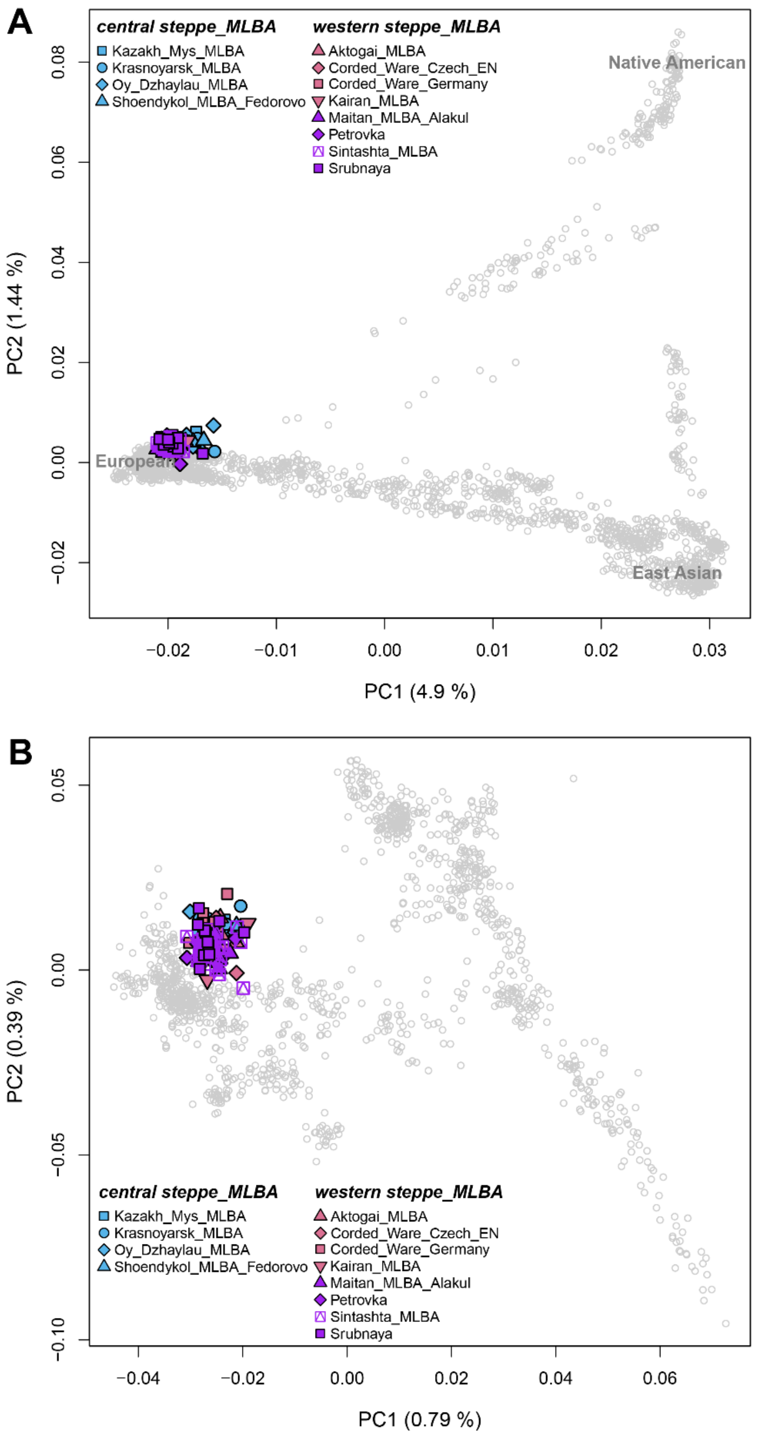

Fig. S14. Principal component analysis of central steppe_MLBA and western steppe_MLBA populations. (**A**) Top two principal components (PCs) calculated from 2,270 present-day Eurasian and American individuals. (**B**) Top two PCs calculated from 1,238 present-day western Eurasian individuals. Both in (**A, B**), grey circles mark present-day individuals used for calculating PCs. Ancient individuals, marked by color-filled symbols, were projected onto the calculated PCs. Central steppe_MLBA populations (n=4) are shown in blue tones, while western steppe_MLBA populations (n=8) are shown in green tones.

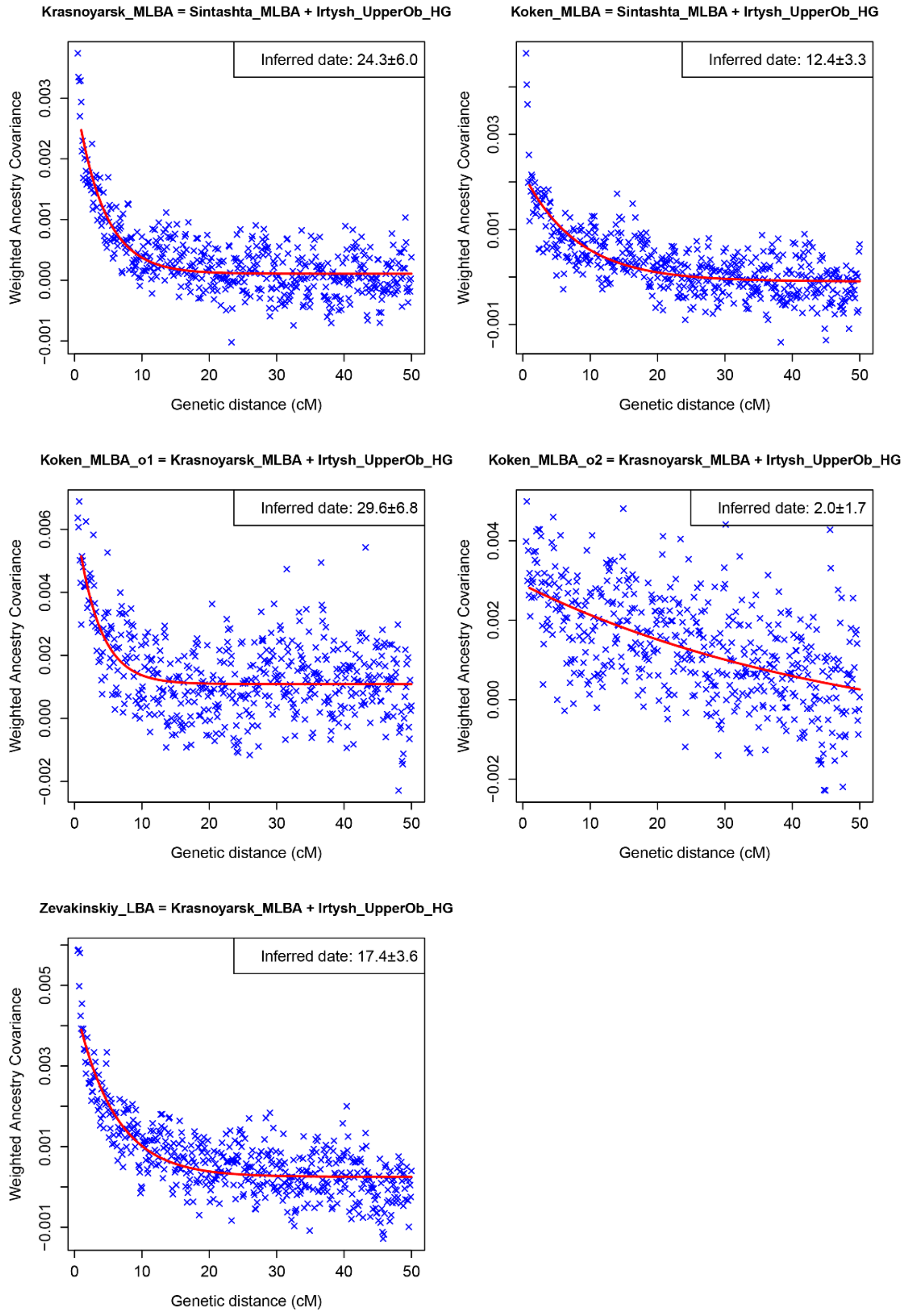

Fig. S15. Admixture dating in the target MLBA groups. We show the weighted ancestry covariance (y-axis) calculated from DATES which is expected to decay exponentially along genetic distance (x-axis) with a decay rate indicating the time since admixture, and fitted exponential curves (shown in red line). We start the fit at genetic distance at 0.5 centiMorgans, and estimate s.e.m. by a leave-one-chromosome-out jackknifing approach as implemented in the DATES program. For Zevakinskiy_LBA, we removed one individual (I3763) who was dated to ca. 1,500 BCE, much earlier than the other individuals.

Table S1. Radiocarbon dates obtained from EN and MLBA human bone collagen at Koken. Radiocarbon dates for two EN and four MLBA specimens are added in this study, totaling three EN and seven MLBA date measures from Koken. For each date, we report the lab code, uncalibrated date BP (with its associated s.e.m.), calibrated date ranges in BC (both 1σ and 2σ).

**Lab Code**	**Sample**	**Period**	**Uncal. date BP**	**Cal. date BC, 68.2% (1σ)**	**Cal. date BC, 95.4% (2σ)**	**Reference**
MAMS-57840	KKN001 (KKOP6_ ctx29H.2)	EN	6452 ± 26	5473-5381	5477-5369	This study
UGAM-67986	KKN001 (KKOP6_ ctx29H.2)	EN	6460 ± 30	5473-5383	5479-5369	This study
Beta-570765	KKN002 (KKOP6_ ctx29H.1)	EN	6370 ± 30	5374-5311	5471-5222	Dupuy et al. (24)
ICA19B/10123	KKN084 (KKBR4)	MLBA	3180 ± 30	1496-1428	1507-1407	Dupuy et al. (23)
ICA19B/10116	KKN086 (KKBR8)	MLBA	3400 ± 30	1742-1660	1767-1623	Dupuy et al. (23)
ICA19B/10117	KKN087 (KKBR9)	MLBA	3260 ± 30	1607-1500	1616-1454	Dupuy et al. (23)
Beta-570764	KKN088 (KKBR10)	MLBA	3260 ± 30	1607-1500	1616-1454	This study
Beta-570766	KKN090 (KKBR13)	MLBA	3260 ± 30	1607-1500	1616-1454	This study
MAMS-73115	KKN094 (KKBR15.1)	MLBA	3391 ± 23	1733-1631	1743-1620	This study
MAMS-73116	KKN099 (KKBR19)	MLBA	3436 ± 23	1867-1690	1875-1637	This study

Data S1 to S8. (separate file)

Data S1. Summary details of samples presented in this study. For each Koken individual, we provide their bioarchaeological metadata and sequencing summary for each library. For the sequencing summary part, nr.all, nr.trimmed, nr.mapped, nr.rmdup, nr.rmdup.q30 refer to the total number of reads sequenced, the number of reads after trimming Illumina adapter sequences, the number of reads mapped to the human reference genome, the number of unique mapped reads, and the number of unique mapped reads with mapping quality score 30 or higher, respectively. Len.trimmed and %.human refer to the average length of reads after adapter trimming and the proportion of trimmed reads mapped to the human reference genome, respectively. DMG51 and DMG52 refer to the proportion of C>T misincorporations at the first and second position at the 5' end, respectively. DMG31 and DMG32 refer to the proportion of G>A misincorporations at the last and the second-to-the-last position at the 3' end, respectively. Cov.auto/X/Y/MT refer to the average depth of coverage of autosomal, X, Y, and mitochondrial sites among the 1240K panel SNPs (for MT whole mitochondria was used). X.cont and X.cont.SE refer to the nuclear contamination estimates and their associated standard errors, respectively, calculated from the contamination module in ANGSD, using the Methods of Moment (MoM) estimator of Method 1. MT.cont refers to the mitochondrial contamination estimates from Schmutzi. n1240K, p1240K, nHO, pHO refer to the number and proportion of SNPs covered by each library among the 1240K and HumanOrigins panels, respectively. MThap and Yhap represent the mitochondrial and Y haplogroup assignments.

Data S2. Meta information of main published samples used in this study. We present the meta information for the primary samples utilized in this study, sourced from the respective publications. For calibrated dates, we provide 2-sigma (95%) calibrated dates in BCE. Those marked with asterisks (*) are archaeological dating results. Those marked with hash tags (#) are the midpoints of the calibrated dates as provided in the original publication.

Data S3. The whole list of published populations used in this study. For each analysis group, we provide the group label, the number of individuals included in the group (N), whether the group is ancient or present-day ("Ancient"), whether the group is included in the 1240K and HO datasets, and whether the group is used for PCA, f-statistics, qpWave/qpAdm, and DATES analyses. The "raw data type" column indicates whether previously reported 1240K pull-down haploid genotype was used (1240K) or whether the new pulldown genotype data was produced from the published BAM file (BAM). For ancient groups, we provide the full list of individual IDs included in each group for enhancing replicability of the study.

Data S4. Relatedness inference results for EN and MLBA Koken individuals. (**A**) For the two EN Koken individuals, we provide the full list of inferred IBD segments between them from ancIBD. For each segment, we provide the starting and ending index number of SNPs (Stant and End), the number of SNPs included in the segment (Length), the starting and ending positions in genetic distance (Start (M) and End (M)), the length of the segment in genetic distance (Length (M)), the starting and ending positions in physical distance (Start (bp) and End (bp)), and the segment type (IBD1 or IBD2). (**B**) For each pair of the Koken EN and (**C**) MLBA individuals, we present the number of SNPs genotyped in both individual (nSNPs), the number of such SNPs with different genotypes (nmismatch), pairwise genotype mismatch rate (PMR), and their putative relationship. No record in the relationship column suggests no discernible close kinship

in the pair. Pairs with nSNPs<2,000 were marked in grey color due to their low credibility of the PMR estimates.

Data S5. The genetic affinity between target populations and worldwide populations. We calculate f3(Mbuti; Worldwide, target) to measure shared genetic affinity between the target population and worldwide populations. The results are listed in the order of Koken_EN1, Koken_EN2, Koken_MLBA, Koken_MLBA_o1, and Koken_MLBA_o2. Standard errors (se) are calculated by 5 cM block jackknifing as implemented in the qp3pop function from admixtools2.

Data S6. F4 symmetry test results between target populations. We calculate f4(Mbuti, Worldwide; target1, target2) to test whether ancient or present-day worldwide groups are genetically closer to one than the other. Standard errors (se) are calculated by 5 cM block jackknifing as implemented in the f4 function from admixtools2. Results with |Z| > 3 are highlighted in yellow.

Data S7. QpAdm and qpWave modeling of EN Koken and Siberian forest-steppe ancient individuals. We provide (**A**) proximal qpWave/qpAdm admixture modeling results for Koken EN individuals, (**B-C**) distal qpAdm modeling results for the Siberian forest-steppe hunter-gatherer populations to compare candidate proxies for APS and EEHG ancestry, (**D**) distal qpAdm modeling results for the Siberian forest-steppe hunter-gatherer individuals, (**E-F**) proximal qpWave and qpAdm modeling results for Tobol_HG and Irtysh_HG individuals, and (**G**) proximal qpAdm modeling results on Tobol_HG and Irtysh_HG populations. For each model, we present qpWave/qpAdm p-value ("p-value"), admixture coefficients ("coeff"), and the associated 5 cM block jackknifing s.e.m. ("SE").

Data S8. QpAdm and qpWave modeling of MLBA Koken and Siberian forest-steppe ancient individuals. We provide (**A**) qpWave results for Koken_MLBA, central steppe_MLBA and western steppe_MLBA populations, (**B**) proximal qpAdm modeling results for central steppe_MLBA populations, (**C**) evaluation of Koken_EN1 as an ancestral proxy in proximal qpAdm models of Koken_MLBA and Krasnoyarsk_MLBA, and (**D**) proximal qpAdm modeling results for the steppe MLBA outlier individuals. For each model, we present qpWave/qpAdm p-value ("p-value"), admixture coefficients ("coeff"), and the associated 5 cM block jackknifing s.e.m. ("SE").

REFERENCES AND NOTES

1. C. Jeong, O. Balanovsky, E. Lukianova, N. Kahbatkyzy, P. Flegontov, V. Zaporozhchenko, A. Immel, C.-C. Wang, O. Ixan, E. Khussainova, B. Bekmanov, V. Zaibert, M. Lavryashina, E. Pocheshkhova, Y. Yusupov, A. Agdzhoyan, S. Koshel, A. Bukin, P. Nymadawa, S. Turdikulova, D. Dalimova, M. Churnosov, R. Skhalyakho, D. Daragan, Y. Bogunov, A. Bogunova, A. Shtrunov, N. Dubova, M. Zhabagin, L. Yepiskoposyan, V. Churakov, N. Pislegin, L. Damba, L. Saroyants, K. Dibirova, L. Atramentova, O. Utevska, E. Idrisov, E. Kamenshchikova, I. Evseeva, M. Metspalu, A. K. Outram, M. Robbeets, L. Djansugurova, E. Balanovska, S. Schiffels, W. Haak, D. Reich, J. Krause, The genetic history of admixture across inner Eurasia. *Nat. Eco. Evo.* **3**, 966–976 (2019).

2. M. E. Allentoft, M. Sikora, K.-G. Sjögren, S. Rasmussen, M. Rasmussen, J. Stenderup, P. B. Damgaard, H. Schroeder, T. Ahlström, L. Vinner, A.-S. Malaspinas, A. Margaryan, T. Higham, D. Chivall, N. Lynnerup, L. Harvig, J. Baron, P. D. Casa, P. Dąbrowski, P. R. Duffy, A. V. Ebel, A. Epimakhov, K. Frei, M. Furmanek, T. Gralak, A. Gromov, S. Gronkiewicz, G. Grupe, T. Hajdu, R. Jarysz, V. Khartanovich, A. Khokhlov, V. Kiss, J. Kolář, A. Kriiska, I. Lasak, C. Longhi, G. M. Glynn, A. Merkevicius, I. Merkyte, M. Metspalu, R. Mkrtchyan, V. Moiseyev, L. Paja, G. Pálfi, D. Pokutta, Ł. Pospieszny, T. D. Price, L. Saag, M. Sablin, N. Shishlina, V. Smrčka, V. I. Soenov, V. Szeverényi, G. Tóth, S. V. Trifanova, L. Varul, M. Vicze, L. Yepiskoposyan, V. Zhitenev, L. Orlando, T. Sicheritz-Pontén, S. Brunak, R. Nielsen, K. Kristiansen, E. Willerslev, Population genomics of Bronze Age Eurasia. *Nature* **522**, 167–172 (2015).

3. I. Lazaridis, D. Nadel, G. Rollefson, D. C. Merrett, N. Rohland, S. Mallick, D. Fernandes, M. Novak, B. Gamarra, K. Sirak, S. Connell, K. Stewardson, E. Harney, Q. Fu, G. Gonzalez-Fortes, E. R. Jones, S. A. Roodenberg, G. Lengyel, F. Bocquentin, B. Gasparian, J. M. Monge, M. Gregg, V. Eshed, A.-S. Mizrahi, C. Meiklejohn, F. Gerritsen, L. Bejenaru, M. Blüher, A. Campbell, G. Cavalleri, D. Comas, P. Froguel, E. Gilbert, S. M. Kerr, P. Kovacs, J. Krause, D. McGettigan, M. Merrigan, D. A. Merriwether, S. O'Reilly, M. B. Richards, O. Semino, M. Shamoon-Pour, G. Stefanescu, M. Stumvoll, A. Tönjes, A. Torroni, J. F. Wilson, L. Yengo, N. A. Hovhannisyan, N. Patterson, R. Pinhasi, D. Reich, Genomic insights into the origin of farming in the ancient Near East. *Nature* **536**, 419–424 (2016).

4. C. Jeong, K. Wang, S. Wilkin, W. T. T. Taylor, B. K. Miller, J. H. Bemmann, R. Stahl, C. Chiovelli, F. Knolle, S. Ulziibayar, D. Khatanbaatar, D. Erdenebaatar, U. Erdenebat, A. Ochir, G. Ankhsanaa, C. Vanchigdash, B. Ochir, C. Munkhbayar, D. Tumen, A. Kovalev, N. Kradin, B. A. Bazarov, D. A. Miyagashev, P. B. Konovalov, E. Zhambaltarova, A. V. Miller, W. Haak, S. Schiffels, J. Krause, N. Boivin, M. Erdene, J. Hendy, C. Warinner, A dynamic 6,000-year genetic history of Eurasia's Eastern Steppe. *Cell* **183**, 890–904.e29 (2020).

5. E. Skourtanioti, Y. S. Erdal, M. Frangipane, F. Balossi Restelli, K. A. Yener, F. Pinnock, P. Matthiae, R. Özbal, U.-D. Schoop, F. Guliyev, T. Akhundov, B. Lyonnet, E. L. Hammer, S. E. Nugent, M. Burri, G. U. Neumann, S. Penske, T. Ingman, M. Akar, R. Shafiq, G. Palumbi, S. Eisenmann, M. D'Andrea, A. B. Rohrlach, C. Warinner, C. Jeong, P. W. Stockhammer, W. Haak, J. Krause, Genomic History of Neolithic to Bronze Age Anatolia, Northern Levant, and Southern Caucasus. *Cell* **181**, 1158–1175.e28 (2020).

6. G. A. Gnecchi-Ruscone, E. Khussainova, N. Kahbatkyzy, L. Musralina, M. A. Spyrou, R. A. Bianco, R. Radzeviciute, N. F. G. Martins, C. Freund, O. Iksan, A. Garshin, Z. Zhaniyazov, B. Bekmanov, E. Kitov, Z. Samashev, A. Beisenov, N. Berezina, Y. Berezin, A. Z. Bíró, S. Évinger, A. Bissembaev, G. Akhatov, A. Mamedov, A. Onggaruly, D. Voyakin, A. Chotbayev, Y. Kariyev, A. Buzhilova, L. Djansugurova, C. Jeong, J. Krause, Ancient genomic time transect from the Central Asian Steppe unravels the history of the Scythians. *Sci. Adv.* **7**, eabe4414 (2021).

7. C.-C. Wang, H.-Y. Yeh, A. N. Popov, H.-Q. Zhang, H. Matsumura, K. Sirak, O. Cheronet, A. Kovalev, N. Rohland, A. M. Kim, S. Mallick, R. Bernardos, D. Tumen, J. Zhao, Y.-C. Liu, J.-Y. Liu, M. Mah, K. Wang, Z. Zhang, N. Adamski, N. Broomandkhoshbacht, K. Callan, F. Candilio, K. S. D. Carlson, B. J. Culleton, L. Eccles, S. Freilich, D. Keating, A. M. Lawson, K. Mandl, M. Michel, J. Oppenheimer, K. T. Özdoğan, K. Stewardson, S. Wen, S. Yan, F. Zalzala, R. Chuang, C.-J. Huang, H. Looh, C.-C. Shiung, Y. G. Nikitin, A. V. Tabarev, A. A. Tishkin, S. Lin, Z.-Y. Sun, X.-M. Wu, T.-L. Yang, X. Hu, L. Chen, H. Du, J. Bayarsaikhan, E. Mijiddorj, D. Erdenebaatar, T.-O. Iderkhangai, E. Myagmar, H. Kanzawa-Kiriyama, M. Nishino, K.-i. Shinoda, O. A. Shubina, J. Guo, W. Cai, Q. Deng, L. Kang, D. Li, D. Li, R. Lin, Nini, R. Shrestha, L.-X. Wang, L. Wei, G. Xie, H. Yao, M. Zhang, G. He, X. Yang, R. Hu, M.

Robbeets, S. Schiffels, D. J. Kennett, L. Jin, H. Li, J. Krause, R. Pinhasi, D. Reich, Genomic insights into the formation of human populations in East Asia. *Nature* **591**, 413–419 (2021).

8. M. E. Allentoft, M. Sikora, A. Refoyo-Martínez, E. K. Irving-Pease, A. Fischer, W. Barrie, A. Ingason, J. Stenderup, K.-G. Sjögren, A. Pearson, B. S. da Mota, B. S. Paulsson, A. Halgren, R. Macleod, M. L. S. Jørkov, F. Demeter, L. Sørensen, P. O. Nielsen, R. A. Henriksen, T. Vimala, H. McColl, A. Margaryan, M. Ilardo, A. Vaughn, M. F. Mortensen, A. B. Nielsen, M. U. Hede, N. N. Johannsen, P. Rasmussen, L. Vinner, G. Renaud, A. Stern, T. Z. T. Jensen, G. Scorrano, H. Schroeder, P. Lysdahl, A. D. Ramsøe, A. Skorobogatov, A. J. Schork, A. Rosengren, A. Ruter, A. Outram, A. A. Timoshenko, A. Buzhilova, A. Coppa, A. Zubova, A. M. Silva, A. J. Hansen, A. Gromov, A. Logvin, A. B. Gotfredsen, B. H. Nielsen, B. González-Rabanal, C. Lalueza-Fox, C. J. McKenzie, C. Gaunitz, C. Blasco, C. Liesau, C. Martinez-Labarga, D. V. Pozdnyakov, D. Cuenca-Solana, D. O. Lordkipanidze, D. En'shin, D. C. Salazar-García, T. D. Price, D. Borić, E. Kostyleva, E. V. Veselovskaya, E. R. Usmanova, E. Cappellini, E. B. Petersen, E. Kannegaard, F. Radina, F. E. Yediay, H. Duday, I. Gutiérrez-Zugasti, I. Merts, I. Potekhina, I. Shevnina, I. Altinkaya, J. Guilaine, J. Hansen, J. E. A. Tortosa, J. Zilhão, J. Vega, K. B. Pedersen, K. Tunia, L. Zhao, L. N. Mylnikova, L. Larsson, L. Metz, L. Yepiskoposyan, L. Pedersen, L. Sarti, L. Orlando, L. Slimak, L. Klassen, M. Blank, M. González-Morales, M. Silvestrini, M. Vretemark, M. S. Nesterova, M. Rykun, M. F. Rolfo, M. Szmyt, M. Przybyła, M. Calattini, M. Sablin, M. Dobisíková, M. Meldgaard, M. Johansen, N. Berezina, N. Card, N. A. Saveliev, O. Poshekhonova, O. Rickards, O. V. Lozovskaya, O. Gábor, O. C. Uldum, P. Aurino, P. Kosintsev, P. Courtaud, P. Ríos, P. Mortensen, P. Lotz, P. Persson, P. Bangsgaard, P. de Barros Damgaard, P. V. Petersen, P. P. Martinez, P. Włodarczak, R. V. Smolyaninov, R. Maring, R. Menduiña, R. Badalyan, R. Iversen, R. Turin, S. Vasilyev, S. Wåhlin, S. Borutskaya, S. Skochina, S. A. Sørensen, S. H. Andersen, T. Jørgensen, Y. B. Serikov, V. I. Molodin, V. Smrcka, V. Merts, V. Appadurai, V. Moiseyev, Y. Magnusson, K. H. Kjær, N. Lynnerup, D. J. Lawson, P. H. Sudmant, S. Rasmussen, T. S. Korneliussen, R. Durbin, R. Nielsen, O. Delaneau, T. Werge, F. Racimo, K. Kristiansen, E. Willerslev, Population genomics of post-glacial western Eurasia. *Nature* **625**, 301–311 (2024).

9. P. de Barros Damgaard, R. Martiniano, J. Kamm, J. V. Moreno-Mayar, G. Kroonen, M. Peyrot, G. Barjamovic, S. Rasmussen, C. Zacho, N. Baimukhanov, V. Zaibert, V. Merz, A. Biddanda, I. Merz, V. Loman, V. Evdokimov, E. Usmanova, B. Hemphill, A. Seguin-Orlando, F. E.

Yediay, I. Ullah, K.-G. Sjögren, K. H. Iversen, J. Choin, C. de la Fuente, M. Ilardo, H. Schroeder, V. Moiseyev, A. Gromov, A. Polyakov, S. Omura, S. Y. Senyurt, H. Ahmad, C. McKenzie, A. Margaryan, A. Hameed, A. Samad, N. Gul, M. H. Khokhar, O. I. Goriunova, V. I. Bazaliiskii, J. Novembre, A. W. Weber, L. Orlando, M. E. Allentoft, R. Nielsen, K. Kristiansen, M. Sikora, A. K. Outram, R. Durbin, E. Willerslev, The first horse herders and the impact of early Bronze Age steppe expansions into Asia. *Science* **360**, eaar7711 (2018).

10. M. Raghavan, P. Skoglund, K. E. Graf, M. Metspalu, A. Albrechtsen, I. Moltke, S. Rasmussen, T. W. Stafford Jr., L. Orlando, E. Metspalu, M. Karmin, K. Tambets, S. Rootsi, R. Mägi, P. F. Campos, E. Balanovska, O. Balanovsky, E. Khusnutdinova, S. Litvinov, L. P. Osipova, S. A. Fedorova, M. I. Voevoda, M. DeGiorgio, T. Sicheritz-Ponten, S. Brunak, S. Demeshchenko, T. Kivisild, R. Villems, R. Nielsen, M. Jakobsson, E. Willerslev, Upper Palaeolithic Siberian genome reveals dual ancestry of Native Americans. *Nature* **505**, 87–91 (2014).

11. H. Yu, M. A. Spyrou, M. Karapetian, S. Shnaider, R. Radzevičiūtė, K. Nägele, G. U. Neumann, S. Penske, J. Zech, M. Lucas, P. LeRoux, P. Roberts, G. Pavlenok, A. Buzhilova, C. Posth, C. Jeong, J. Krause, Paleolithic to Bronze Age Siberians Reveal Connections with First Americans and across Eurasia. *Cell* **181**, 1232–1245.e20 (2020).

12. F. Zhang, C. Ning, A. Scott, Q. Fu, R. Bjørn, W. Li, D. Wei, W. Wang, L. Fan, I. Abuduresule, X. Hu, Q. Ruan, A. Niyazi, G. Dong, P. Cao, F. Liu, Q. Dai, X. Feng, R. Yang, Z. Tang, P. Ma, C. Li, S. Gao, Y. Xu, S. Wu, S. Wen, H. Zhu, H. Zhou, M. Robbeets, V. Kumar, J. Krause, C. Warinner, C. Jeong, Y. Cui, The genomic origins of the Bronze Age Tarim Basin mummies. *Nature* **599**, 256–261 (2021).

13. K. Wang, H. Yu, R. Radzevičiūtė, Y. F. Kiryushin, A. A. Tishkin, Y. V. Frolov, N. F. Stepanova, K. Y. Kiryushin, A. L. Kungurov, S. V. Shnaider, S. S. Tur, M. P. Tiunov, A. V. Zubova, M. Pevzner, T. Karimov, A. Buzhilova, V. Slon, C. Jeong, J. Krause, C. Posth, Middle Holocene Siberian genomes reveal highly connected gene pools throughout North Asia. *Curr. Biol.* **33**, 423–433.e5 (2023).

14. J. Bemmann, D. Batsükh, J.-O. Gantulga, C. Yeruul-Erdene, U. Brosseder, "Searching for patterns through the ages in ritual landscapes of Bronze Age Mongolia" in *Man sieht nur, was*

man weiß. Man weiß nur, was man sieht. Globalhistorische Perspektiven auf interkulturelle Phänomene der Mobilität. Prähistorische Archäologie in Südosteuropa 33, J. Schneeweiss, M. Nawroth, H. Piezonka, H. Schwarzberg, Eds. (VML Vlg Marie Leidorf, 2024).

15. W. Honeychurch, *Inner Asia and the Spatial Politics of Empire: Archaeology, Mobility, and Culture Contact* (Springer, 2015).

16. S. Grigoriev, Andronovo problem: Studies of cultural genesis in the Eurasian Bronze Age. *Open Archaeol.* **7**, 3–36 (2021).

17. Q. Fu, C. Posth, M. Hajdinjak, M. Petr, S. Mallick, D. Fernandes, A. Furtwängler, W. Haak, M. Meyer, A. Mittnik, B. Nickel, A. Peltzer, N. Rohland, V. Slon, S. Talamo, I. Lazaridis, M. Lipson, I. Mathieson, S. Schiffels, P. Skoglund, A. P. Derevianko, N. Drozdov, V. Slavinsky, A. Tsybankov, R. G. Cremonesi, F. Mallegni, B. Gély, E. Vacca, M. R. G. Morales, L. G. Straus, C. Neugebauer-Maresch, M. Teschler-Nicola, S. Constantin, O. T. Moldovan, S. Benazzi, M. Peresani, D. Coppola, M. Lari, S. Ricci, A. Ronchitelli, F. Valentin, C. Thevenet, K. Wehrberger, D. Grigorescu, H. Rougier, I. Crevecoeur, D. Flas, P. Semal, M. A. Mannino, C. Cupillard, H. Bocherens, N. J. Conard, K. Harvati, V. Moiseyev, D. G. Drucker, J. Svoboda, M. P. Richards, D. Caramelli, R. Pinhasi, J. Kelso, N. Patterson, J. Krause, S. Pääbo, D. Reich, The genetic history of Ice Age Europe. *Nature* **534**, 200–205 (2016).

18. S. Reichert, N.-O. Erdene-Ochir, J. Bemmann, A unique burial of the fourth millennium B.C.E. and the earliest burial traditions in Mongolia. *Asian Perspect.* **61**, 220–252 (2022).

19. S. Wilkin, A. Ventresca Miller, R. Fernandes, R. Spengler, W. T. T. Taylor, D. R. Brown, D. Reich, D. J. Kennett, B. J. Culleton, L. Kunz, C. Fortes, A. Kitova, P. Kuznetsov, A. Epimakhov, V. F. Zaibert, A. K. Outram, E. Kitov, A. Khokhlov, D. Anthony, N. Boivin, Dairying enabled Early Bronze Age Yamnaya steppe expansions. *Nature* **598**, 629–633 (2021).

20. T. R. Hermes, M. D. Frachetti, D. Voyakin, A. S. Yerlomaeva, A. Z. Beisenov, P. N. Doumani Dupuy, D. V. Papin, G. Motuzaite Matuzeviciute, J. Bayarsaikhan, J.-L. Houle, A. A. Tishkin, A. Nebel, B. Krause-Kyora, C. A. Makarewicz, High mitochondrial diversity of domesticated

goats persisted among Bronze and Iron Age pastoralists in the Inner Asian Mountain Corridor. *PLOS ONE* **15**, e0233333 (2020).

21. M. D. Frachetti, Multiregional emergence of mobile pastoralism and nonuniform institutional complexity across Eurasia. *Curr. Anthropol.* **53**, 2–38 (2012).

22. D. W. Anthony, *The Horse, the Wheel, and Language: How Bronze-Age Riders from the Eurasian Steppes Shaped the Modern World* (Princeton Univ. Press, 2008).

23. P. N. Doumani Dupuy, A. S. Zhuniskhanov, E. A. Bullion, G. K. Kiyasbek, Z. K. Tashmanbetova, E. Z. Rakhmankulov, A. I. Isin, The newly discovered bronze age site of Koken: Merging micro-regions with major study zones in the high steppes of Kazakhstan. *Archaeol. Res. Asia* **27**, 100292 (2021).

24. P. N. Doumani Dupuy, Z. Tashmanbetova, G. Kiyasbek, V. Merts, R. Coil, A. Zhuniskhanov, Z. Samashev, The earliest human burial in Kazakhstan: According to the Neolithic finds of the Koken settlement (Самое древнее захоронение человека в Казахстане: по неолитическим находкам поселения Кокен). *Archaeol. KZ* **21**, 164–186 (2023).

25. I. Mathieson, I. Lazaridis, N. Rohland, S. Mallick, N. Patterson, S. A. Roodenberg, E. Harney, K. Stewardson, D. Fernandes, M. Novak, K. Sirak, C. Gamba, E. R. Jones, B. Llamas, S. Dryomov, J. Pickrell, J. L. Arsuaga, J. M. B. de Castro, E. Carbonell, F. Gerritsen, A. Khokhlov, P. Kuznetsov, M. Lozano, H. Meller, O. Mochalov, V. Moiseyev, M. A. R. Guerra, J. Roodenberg, J. M. Vergès, J. Krause, A. Cooper, K. W. Alt, D. Brown, D. Anthony, C. Lalueza-Fox, W. Haak, R. Pinhasi, D. Reich, Genome-wide patterns of selection in 230 ancient Eurasians. *Nature* **528**, 499–503 (2015).

26. N. Patterson, A. L. Price, D. Reich, Population structure and eigenanalysis. *PLOS Genet.* **2**, e190 (2006).

27. M. Sikora, V. V. Pitulko, V. C. Sousa, M. E. Allentoft, L. Vinner, S. Rasmussen, A. Margaryan, P. d. B. Damgaard, C. de la Fuente, G. Renaud, M. A. Yang, Q. Fu, I. Dupanloup, K. Giampoudakis, D. Nogués-Bravo, C. Rahbek, G. Kroonen, M. Peyrot, H. McColl, S. V. Vasilyev, E. Veselovskaya, M. Gerasimova, E. Y. Pavlova, V. G. Chasnyk, P. A. Nikolskiy, A.

V. Gromov, V. I. Khartanovich, V. Moiseyev, P. S. Grebenyuk, A. Y. Fedorchenko, A. I. Lebedintsev, S. B. Slobodin, B. A. Malyarchuk, R. Martiniano, M. Meldgaard, L. Arppe, J. U. Palo, T. Sundell, K. Mannermaa, M. Putkonen, V. Alexandersen, C. Primeau, N. Baimukhanov, R. S. Malhi, K.-G. Sjögren, K. Kristiansen, A. Wessman, A. Sajantila, M. M. Lahr, R. Durbin, R. Nielsen, D. J. Meltzer, L. Excoffier, E. Willerslev, The population history of northeastern Siberia since the Pleistocene. *Nature* **570**, 182–188 (2019).

28. G. M. Kılınç, N. Kashuba, D. Koptekin, N. Bergfeldt, H. M. Dönertaş, R. Rodríguez-Varela, D. Shergin, G. Ivanov, D. Kichigin, K. Pestereva, D. Volkov, P. Mandryka, A. Kharinskii, A. Tishkin, E. Ineshin, E. Kovychev, A. Stepanov, L. Dalén, T. Günther, E. Kırdök, M. Jakobsson, M. Somel, M. Krzewińska, J. Storå, A. Götherström, Human population dynamics and *Yersinia pestis* in ancient northeast Asia. *Sci. Adv.* **7**, eabc4587 (2021).

29. V. M. Narasimhan, N. Patterson, P. Moorjani, N. Rohland, R. Bernardos, S. Mallick, I. Lazaridis, N. Nakatsuka, I. Olalde, M. Lipson, A. M. Kim, L. M. Olivieri, A. Coppa, M. Vidale, J. Mallory, V. Moiseyev, E. Kitov, J. Monge, N. Adamski, N. Alex, N. Broomandkhoshbacht, F. Candilio, K. Callan, O. Cheronet, B. J. Culleton, M. Ferry, D. Fernandes, S. Freilich, B. Gamarra, D. Gaudio, M. Hajdinjak, É. Harney, T. K. Harper, D. Keating, A. M. Lawson, M. Mah, K. Mandl, M. Michel, M. Novak, J. Oppenheimer, N. Rai, K. Sirak, V. Slon, K. Stewardson, F. Zalzala, Z. Zhang, G. Akhatov, A. N. Bagashev, A. Bagnera, B. Baitanayev, J. Bendezu-Sarmiento, A. A. Bissembaev, G. L. Bonora, T. T. Chargynov, T. Chikisheva, P. K. Dashkovskiy, A. Derevianko, M. Dobeš, K. Douka, N. Dubova, M. N. Duisengali, D. Enshin, A. Epimakhov, A. V. Fribus, D. Fuller, A. Goryachev, A. Gromov, S. P. Grushin, B. Hanks, M. Judd, E. Kazizov, A. Khokhlov, A. P. Krygin, E. Kupriyanova, P. Kuznetsov, D. Luiselli, F. Maksudov, A. M. Mamedov, T. B. Mamirov, C. Meiklejohn, D. C. Merrett, R. Micheli, O. Mochalov, S. Mustafokulov, A. Nayak, D. Pettener, R. Potts, D. Razhev, M. Rykun, S. Sarno, T. M. Savenkova, K. Sikhymbaeva, S. M. Slepchenko, O. A. Soltobaev, N. Stepanova, S. Svyatko, K. Tabaldiev, M. Teschler-Nicola, A. A. Tishkin, V. V. Tkachev, S. Vasilyev, P. Velemínský, D. Voyakin, A. Yermolayeva, M. Zahir, V. S. Zubkov, A. Zubova, V. S. Shinde, C. Lalueza-Fox, M. Meyer, D. Anthony, N. Boivin, K. Thangaraj, D. J. Kennett, M. Frachetti, R. Pinhasi, D. Reich, The formation of human populations in South and Central Asia. *Science* **365**, eaat7487 (2019).

30. D. J. Kennett, S. Plog, R. J. George, B. J. Culleton, A. S. Watson, P. Skoglund, N. Rohland, S. Mallick, K. Stewardson, L. Kistler, S. A. LeBlanc, P. M. Whiteley, D. Reich, G. H. Perry, Archaeogenomic evidence reveals prehistoric matrilineal dynasty. *Nat. Commun.* **8**, 14115 (2017).

31. H. Ringbauer, Y. Huang, A. Akbari, S. Mallick, I. Olalde, N. Patterson, D. Reich, Accurate detection of identity-by-descent segments in human ancient DNA. *Nat. Genet.* **56**, 143–151 (2024).

32. N. Patterson, P. Moorjani, Y. Luo, S. Mallick, N. Rohland, Y. Zhan, T. Genschoreck, T. Webster, D. Reich, Ancient admixture in human history. *Genetics* **192**, 1065–1093 (2012).

33. D. Reich, N. Patterson, D. Campbell, A. Tandon, S. Mazieres, N. Ray, M. V. Parra, W. Rojas, C. Duque, N. Mesa, L. F. García, O. Triana, S. Blair, A. Maestre, J. C. Dib, C. M. Bravi, G. Bailliet, D. Corach, T. Hünemeier, M. C. Bortolini, F. M. Salzano, M. L. Petzl-Erler, V. Acuña-Alonzo, C. Aguilar-Salinas, S. Canizales-Quinteros, T. Tusié-Luna, L. Riba, M. Rodríguez-Cruz, M. Lopez-Alarcón, R. Coral-Vazquez, T. Canto-Cetina, I. Silva-Zolezzi, J. C. Fernandez-Lopez, A. V. Contreras, G. Jimenez-Sanchez, M. J. Gómez-Vázquez, J. Molina, Á. Carracedo, A. Salas, C. Gallo, G. Poletti, D. B. Witonsky, G. Alkorta-Aranburu, R. I. Sukernik, L. Osipova, S. A. Fedorova, R. Vasquez, M. Villena, C. Moreau, R. Barrantes, D. Pauls, L. Excoffier, G. Bedoya, F. Rothhammer, J.-M. Dugoujon, G. Larrouy, W. Klitz, D. Labuda, J. Kidd, K. Kidd, A. Di Rienzo, N. B. Freimer, A. L. Price, A. Ruiz-Linares, Reconstructing native American population history. *Nature* **488**, 370–374 (2012).

34. C. Posth, H. Yu, A. Ghalichi, H. Rougier, I. Crevecoeur, Y. Huang, H. Ringbauer, A. B. Rohrlach, K. Nägele, V. Villalba-Mouco, R. Radzeviciute, T. Ferraz, A. Stoessel, R. Tukhbatova, D. G. Drucker, M. Lari, A. Modi, S. Vai, T. Saupe, C. L. Scheib, G. Catalano, L. Pagani, S. Talamo, H. Fewlass, L. Klaric, A. Morala, M. Rué, S. Madelaine, L. Crépin, J.-B. Caverne, E. Bocaege, S. Ricci, F. Boschin, P. Bayle, B. Maureille, F. Le Brun-Ricalens, J.-G. Bordes, G. Oxilia, E. Bortolini, O. Bignon-Lau, G. Debout, M. Orliac, A. Zazzo, V. Sparacello, E. Starnini, L. Sineo, J. van der Plicht, L. Pecqueur, G. Merceron, G. Garcia, J.-M. Leuvrey, C. B. Garcia, A. Gómez-Olivencia, M. Połtowicz-Bobak, D. Bobak, M. Le Luyer, P. Storm, C. Hoffmann, J. Kabaciński, T. Filimonova, S. Shnaider, N. Berezina, B.

González-Rabanal, M. R. González Morales, A. B. Marín-Arroyo, B. López, C. Alonso-Llamazares, A. Ronchitelli, C. Polet, I. Jadin, N. Cauwe, J. Soler, N. Coromina, I. Rufí, R. Cottiaux, G. Clark, L. G. Straus, M.-A. Julien, S. Renhart, D. Talaa, S. Benazzi, M. Romandini, L. Amkreutz, H. Bocherens, C. Wißing, S. Villotte, J. F.-L. de Pablo, M. Gómez-Puche, M. A. Esquembre-Bebia, P. Bodu, L. Smits, B. Souffi, R. Jankauskas, J. Kozakaitė, C. Cupillard, H. Benthien, K. Wehrberger, R. W. Schmitz, S. C. Feine, T. Schüler, C. Thevenet, D. Grigorescu, F. Lüth, A. Kotula, H. Piezonka, F. Schopper, J. Svoboda, S. Sázelová, A. Chizhevsky, A. Khokhlov, N. J. Conard, F. Valentin, K. Harvati, P. Semal, B. Jungklaus, A. Suvorov, R. Schulting, V. Moiseyev, K. Mannermaa, A. Buzhilova, T. Terberger, D. Caramelli, E. Altena, W. Haak, J. Krause, Palaeogenomics of upper palaeolithic to neolithic european hunter-gatherers. *Nature* **615**, 117–126 (2023).

35. I. Lazaridis, N. Patterson, D. Anthony, L. Vyazov, R. Fournier, H. Ringbauer, I. Olalde, A. A. Khokhlov, E. P. Kitov, N. I. Shishlina, S. C. Ailincăi, D. S. Agapov, S. A. Agapov, E. Batieva, B. Bauyrzhan, Z. Bereczki, A. Buzhilova, P. Changmai, A. A. Chizhevsky, I. Ciobanu, M. Constantinescu, M. Csányi, J. Dani, P. K. Dashkovskiy, S. Évinger, A. Faifert, P. Flegontov, A. Frînculeasa, M. N. Frînculeasa, T. Hajdu, T. Higham, P. Jarosz, P. Jelínek, V. I. Khartanovich, E. N. Kirginekov, V. Kiss, A. Kitova, A. V. Kiyashko, J. Koledin, A. Korolev, P. Kosintsev, G. Kulcsár, P. Kuznetsov, R. Magomedov, A. M. Mamedov, E. Melis, V. Moiseyev, E. Molnár, J. Monge, O. Negrea, N. A. Nikolaeva, M. Novak, M. Ochir-Goryaeva, G. Pálfi, S. Popovici, M. P. Rykun, T. M. Savenkova, V. P. Semibratov, N. N. Seregin, A. Šefčáková, R. S. Mussayeva, I. Shingiray, V. N. Shirokov, A. Simalcsik, K. Sirak, K. N. Solodovnikov, J. Tárnoki, A. A. Tishkin, V. Trifonov, S. Vasilyev, A. Akbari, E. S. Brielle, K. Callan, F. Candilio, O. Cheronet, E. Curtis, O. Flegontova, L. Iliev, A. Kearns, D. Keating, A. M. Lawson, M. Mah, A. Micco, M. Michel, J. Oppenheimer, L. Qiu, J. N. Workman, F. Zalzala, A. Szécsényi-Nagy, P. F. Palamara, S. Mallick, N. Rohland, R. Pinhasi, D. Reich, The genetic origin of the Indo-Europeans. *Nature* **639**, 132–142 (2025).

36. I. Mathieson, S. Alpaslan-Roodenberg, C. Posth, A. Szécsényi-Nagy, N. Rohland, S. Mallick, I. Olalde, N. Broomandkhoshbacht, F. Candilio, O. Cheronet, D. Fernandes, M. Ferry, B. Gamarra, G. G. Fortes, W. Haak, E. Harney, E. Jones, D. Keating, B. Krause-Kyora, I. Kucukkalipci, M. Michel, A. Mittnik, K. Nägele, M. Novak, J. Oppenheimer, N. Patterson, S. Pfrengle, K. Sirak, K. Stewardson, S. Vai, S. Alexandrov, K. W. Alt, R. Andreescu, D.

Antonović, A. Ash, N. Atanassova, K. Bacvarov, M. B. Gusztáv, H. Bocherens, M. Bolus, A. Boroneanţ, Y. Boyadzhiev, A. Budnik, J. Burmaz, S. Chohadzhiev, N. J. Conard, R. Cottiaux, M. Čuka, C. Cupillard, D. G. Drucker, N. Elenski, M. Francken, B. Galabova, G. Ganetsovski, B. Gély, T. Hajdu, V. Handzhyiska, K. Harvati, T. Higham, S. Iliev, I. Janković, I. Karavanić, D. J. Kennett, D. Komšo, A. Kozak, D. Labuda, M. Lari, C. Lazar, M. Leppek, K. Leshtakov, D. L. Vetro, D. Los, I. Lozanov, M. Malina, F. Martini, K. McSweeney, H. Meller, M. Menđušić, P. Mirea, V. Moiseyev, V. Petrova, T. D. Price, A. Simalcsik, L. Sineo, M. Šlaus, V. Slavchev, P. Stanev, A. Starović, T. Szeniczey, S. Talamo, M. Teschler-Nicola, C. Thevenet, I. Valchev, F. Valentin, S. Vasilyev, F. Veljanovska, S. Venelinova, E. Veselovskaya, B. Viola, C. Virag, J. Zaninović, S. Zäuner, P. W. Stockhammer, G. Catalano, R. Krauß, D. Caramelli, G. Zariņa, B. Gaydarska, M. Lillie, A. G. Nikitin, I. Potekhina, A. Papathanasiou, D. Borić, C. Bonsall, J. Krause, R. Pinhasi, D. Reich, The genomic history of southeastern Europe. *Nature* **555**, 197–203 (2018).

37. A. G. Nikitin, I. Lazaridis, N. Patterson, S. Ivanova, M. Videiko, V. Dergachev, N. Kotova, M. Lillie, I. Potekhina, M. Krenz-Niedbała, S. Łukasik, S. Makhortykh, V. Renson, H. Shephard, G. Sirbu, S. Svyryd, T. Tkachuk, P. Włodarczak, K. Callan, E. Curtis, E. Harney, L. Iliev, A. Kearns, A. M. Lawson, M. Michel, M. Mah, A. Micco, J. Oppenheimer, L. Qiu, J. N. Workman, F. Zalzala, S. Mallick, N. Rohland, D. Reich, A genomic history of the North Pontic region from the neolithic to the bronze age. *Nature* **639**, 124–131 (2025).

38. T. C. Zeng, L. A. Vyazov, A. Kim, P. Flegontov, K. Sirak, R. Maier, I. Lazaridis, A. Akbari, M. Frachetti, A. A. Tishkin, N. E. Ryabogina, S. A. Agapov, D. S. Agapov, A. N. Alekseev, G. G. Boeskorov, A. P. Derevianko, V. M. Dyakonov, D. N. Enshin, A. V. Fribus, Y. V. Frolov, S. P. Grushin, A. A. Khokhlov, K. Y. Kiryushin, Y. F. Kiryushin, E. P. Kitov, P. Kosintsev, I. V. Kovtun, N. P. Makarov, V. V. Morozov, E. N. Nikolaev, M. P. Rykun, T. M. Savenkova, M. V. Shchelchkova, V. Shirokov, S. N. Skochina, O. S. Sherstobitova, S. M. Slepchenko, K. N. Solodovnikov, E. N. Solovyova, A. D. Stepanov, A. A. Timoshchenko, A. S. Vdovin, A. V. Vybornov, E. V. Balanovska, S. Dryomov, G. Hellenthal, K. Kidd, J. Krause, E. Starikovskaya, R. Sukernik, T. Tatarinova, M. G. Thomas, M. Zhabagin, K. Callan, O. Cheronet, D. Fernandes, D. Keating, F. Candilio, L. Iliev, A. Kearns, K. T. Özdoğan, M. Mah, A. Micco, M. Michel, I. Olalde, F. Zalzala, S. Mallick, N. Rohland, R. Pinhasi, V. M.

Narasimhan, D. Reich, Ancient DNA reveals the prehistory of the Uralic and Yeniseian peoples. *Nature* **644**, 122–132 (2025).

39. C.-C. Wang, S. Reinhold, A. Kalmykov, A. Wissgott, G. Brandt, C. Jeong, O. Cheronet, M. Ferry, E. Harney, D. Keating, S. Mallick, N. Rohland, K. Stewardson, A. R. Kantorovich, V. E. Maslov, V. G. Petrenko, V. R. Erlikh, B. C. Atabiev, R. G. Magomedov, P. L. Kohl, K. W. Alt, S. L. Pichler, C. Gerling, H. Meller, B. Vardanyan, L. Yeganyan, A. D. Rezepkin, D. Mariaschk, N. Berezina, J. Gresky, K. Fuchs, C. Knipper, S. Schiffels, E. Balanovska, O. Balanovsky, I. Mathieson, T. Higham, Y. B. Berezin, A. Buzhilova, V. Trifonov, R. Pinhasi, A. B. Belinskij, D. Reich, S. Hansen, J. Krause, W. Haak, Ancient human genome-wide data from a 3000-year interval in the Caucasus corresponds with eco-geographic regions. *Nat. Commun.* **10**, 590 (2019).

40.. Harney, N. Patterson, D. Reich, J. Wakeley, Assessing the performance of qpAdm: A statistical tool for studying population admixture. *Genetics* **217**, iyaa045 (2021).

41. A. Epimakhov, E. Zazovskaya, I. Alaeva, Migrations and cultural evolution in the light of radiocarbon dating of bronze age sites in the Southern Urals. *Radiocarbon* **66**, 1580–1594 (2024).

42. V. G. Loman, I. A. Kukushkin, "Daryinsky burial ground (Могильник Дарьинский)" in *Archaeology and History of Saryarka*, R. M. Zhumashev, Ed. (Saryarka Archaeological Institute, 2012), pp. 81–106.

43. T. R. Hermes, P. N. Doumani Dupuy, E. R. Henry, M. Meyer, A. N. Mar'yashev, M. D. Frachetti, The multi-period settlement Dali in southeastern Kazakhstan: Bronze Age institutional dynamics along the Inner Asian Mountain Corridor. *Asian Perspect.* **60**, 345–381 (2021).

44. V. K. Merts, Neolithization processes in the northeast Kazakhstan (Processy neolitizatsii v severo-vostochnom Kazakhstane). *Vestnik Omskogo Universiteta. Seriya Istoricheskie nauki* **3**, 99–109 (2018).

45. V. N. Logvin, *Stone Age of Kazakhstan's Pri-Tobol region (Mesolithic–Eneolithic) [Каменный век Казахстанского Притоболья (мезолит–энеолит)]* (Kazakhstan Government Pedagogical Univ. Press, 1991).

46. O. A. Artyukhova, T. B. Mamirov, The Toktaul camp as a unique monument of the Stone Age of Central Kazakhstan (Стоянка Токтаул как уникальный памятник каменного века Центрального Казахстана). *Eurasia Cenozoic Stratigraphy Paleoecology Cultures* **5**, 165–171 (2016).

47. V. V. Varfolomeev, V. G. Loman, V. Yevdokimov, *Kent—A Bronze Age City in the Center of the Kazakh Steppes (Кент – город бронзового века в центре Казахских степей)* (Kazakh Scientific Research Institute of Culture, 2017).

48. I. I. Molodin, *Chicha Settlement of the Transition from the Bronze to Iron Age in the Barabinsk Forest-Steppe (Чича-городище перекходного от бронзы к железу времени в. Барабинскоы лесостепи)* (The Verlag Institute, 2001).

49. S. S. Chernikov, *Eastern Kazakhstan in the Bronze Age (Босточный Казахстан в Эпоху Бронзы)* (Publishing House of the Academy of Sciences, 1960).

50. A. S. Yermolayeva, S. V. Kuzminykh, D. S. Pak, E. V. Dubyagina, Objects of the Bronze Age from the workshops of the foundries of the Taldysai settlement (Central Kazakhstan) [Предметы вооружения эпохи бронзы из мастерских литейщиков поселения Талдысай (Центральный Казахстан)]. *Stratum plus* **2**, 109–120 (2019).

51. I. V. Merts, A. E. Rogozhinsky, "Andronovo settlement of Yelike sazy in Tarbagatai (Андроновское поселение Елике сазы в Тарбагатае)" in *Proceedings of the International Scientific and Methodical Conference «XIII Orazbayev Readings» 20–21 May, 2021,* Z. K. Tuimebayev, Ed. (Kazakh University, 2021), pp. 23–27.

52. V. I. Bazaliyskiy, A. R. Livere, K. M. Haverkort, D. V. Pezhemskiy, A. A. Tyutrin, G. V. Turkin, A. V. Weber, The Early Neolithic burial complex of the Shamanka-II burial ground (based on excavation materials 1991–2003). *Proc. Lab. Ancient Technol.* **4**, 80–103 (2006).

53. L. V. Lbova, E. D. Zhambaltarova, V. P. Konev, *Burial Complexes of the Neolithic—Early Bronze Age of Transbaikalia (formation of archetypes of primitive culture) [Погребальные комплексы неолита – раннего бронзового века Забайкалья (формирование архетипов первобытной культуры)]* (Institute of Archaeology and Ethnography, 2008).

54. W. Haak, I. Lazaridis, N. Patterson, N. Rohland, S. Mallick, B. Llamas, G. Brandt, S. Nordenfelt, E. Harney, K. Stewardson, Q. Fu, A. Mittnik, E. Banffy, C. Economou, M. Francken, S. Friederich, R. G. Pena, F. Hallgren, V. Khartanovich, A. Khokhlov, M. Kunst, P. Kuznetsov, H. Meller, O. Mochalov, V. Moiseyev, N. Nicklisch, S. L. Pichler, R. Risch, M. A. Rojo Guerra, C. Roth, A. Szecsenyi-Nagy, J. Wahl, M. Meyer, J. Krause, D. Brown, D. Anthony, A. Cooper, K. W. Alt, D. Reich, Massive migration from the steppe was a source for Indo-European languages in Europe. *Nature* **522**, 207–211 (2015).

55. P. J. Reimer, W. E. Austin, E. Bard, A. Bayliss, P. G. Blackwell, C. B. Ramsey, M. Butzin, H. Cheng, R. L. Edwards, M. Friedrich, The IntCal20 Northern Hemisphere radiocarbon age calibration curve (0–55 cal kBP). *Radiocarbon* **62**, 725–757 (2020).

56. M. Kircher, "Analysis of high-throughput ancient DNA sequencing data" in *Ancient DNA: Methods and Protocols*, B. Shapiro, M. Hofreiter, Eds. (Humana Press, 2012), chap. 23, pp. 197–228.

57. M.-T. Gansauge, A. Aximu-Petri, S. Nagel, M. Meyer, Manual and automated preparation of single-stranded DNA libraries for the sequencing of DNA from ancient biological remains and other sources of highly degraded DNA. *Nat. Protoc.* **15**, 2279–2300 (2020).

58. H. Li, R. Durbin, Fast and accurate short read alignment with Burrows–Wheeler transform. *Bioinformatics* **25**, 1754–1760 (2009).

59. A. Peltzer, G. Jäger, A. Herbig, A. Seitz, C. Kniep, J. Krause, K. Nieselt, EAGER: Efficient ancient genome reconstruction. *Genome Biol.* **17**, 60 (2016).

60. H. Li, B. Handsaker, A. Wysoker, T. Fennell, J. Ruan, N. Homer, G. Marth, G. Abecasis, R. Durbin, 1000 Genome Project Data Processing Subgroup, The sequence alignment/map format and SAMtools. *Bioinformatics* **25**, 2078–2079 (2009).

61. P. Flegontov, N. E. Altınışık, P. Changmai, N. Rohland, S. Mallick, N. Adamski, D. A. Bolnick, N. Broomandkhoshbacht, F. Candilio, B. J. Culleton, O. Flegontova, T. M. Friesen, C. Jeong, T. K. Harper, D. Keating, D. J. Kennett, A. M. Kim, T. C. Lamnidis, A. M. Lawson, I. Olalde, J. Oppenheimer, B. A. Potter, J. Raff, R. A. Sattler, P. Skoglund, K. Stewardson, E. J. Vajda, S. Vasilyev, E. Veselovskaya, M. G. Hayes, D. H. O'Rourke, J. Krause, R. Pinhasi, D. Reich, S. Schiffels, Palaeo-Eskimo genetic ancestry and the peopling of Chukotka and North America. *Nature* **570**, 236–240 (2019).

62. P. de Barros Damgaard, N. Marchi, S. Rasmussen, M. Peyrot, G. Renaud, T. Korneliussen, J. V. Moreno-Mayar, M. W. Pedersen, A. Goldberg, E. Usmanova, N. Baimukhanov, V. Loman, L. Hedeager, A. G. Pedersen, K. Nielsen, G. Afanasiev, K. Akmatov, A. Aldashev, A. Alpaslan, G. Baimbetov, V. I. Bazaliiskii, A. Beisenov, B. Boldbaatar, B. Boldgiv, C. Dorzhu, S. Ellingvag, D. Erdenebaatar, R. Dajani, E. Dmitriev, V. Evdokimov, K. M. Frei, A. Gromov, A. Goryachev, H. Hakonarson, T. Hegay, Z. Khachatryan, R. Khaskhanov, E. Kitov, A. Kolbina, T. Kubatbek, A. Kukushkin, I. Kukushkin, N. Lau, A. Margaryan, I. Merkyte, I. V. Mertz, V. K. Mertz, E. Mijiddorj, V. Moiyesev, G. Mukhtarova, B. Nurmukhanbetov, Z. Orozbekova, I. Panyushkina, K. Pieta, V. Smrčka, I. Shevnina, A. Logvin, K.-G. Sjögren, T. Štolcová, A. M. Taravella, K. Tashbaeva, A. Tkachev, T. Tulegenov, D. Voyakin, L. Yepiskoposyan, S. Undrakhbold, V. Varfolomeev, A. Weber, M. A. W. Sayres, N. Kradin, M. E. Allentoft, L. Orlando, R. Nielsen, M. Sikora, E. Heyer, K. Kristiansen, E. Willerslev, 137 ancient human genomes from across the Eurasian steppes. *Nature* **557**, 369–374 (2018).

63. I. Lazaridis, A. Mittnik, N. Patterson, S. Mallick, N. Rohland, S. Pfrengle, A. Furtwängler, A. Peltzer, C. Posth, A. Vasilakis, P. J. P. McGeorge, E. Konsolaki-Yannopoulou, G. Korres, H. Martlew, M. Michalodimitrakis, M. Özsait, N. Özsait, A. Papathanasiou, M. Richards, S. A. Roodenberg, Y. Tzedakis, R. Arnott, D. M. Fernandes, J. R. Hughey, D. M. Lotakis, P. A. Navas, Y. Maniatis, J. A. Stamatoyannopoulos, K. Stewardson, P. Stockhammer, R. Pinhasi, D. Reich, J. Krause, G. Stamatoyannopoulos, Genetic origins of the Minoans and Mycenaeans. *Nature* **548**, 214–218 (2017).

64. C. Ning, T. Li, K. Wang, F. Zhang, T. Li, X. Wu, S. Gao, Q. Zhang, H. Zhang, M. J. Hudson, G. Dong, S. Wu, Y. Fang, C. Liu, C. Feng, W. Li, T. Han, R. Li, J. Wei, Y. Zhu, Y. Zhou, C.-C. Wang, S. Fan, Z. Xiong, Z. Sun, M. Ye, L. Sun, X. Wu, F. Liang, Y. Cao, X. Wei, H.

Zhu, H. Zhou, J. Krause, M. Robbeets, C. Jeong, Y. Cui, Ancient genomes from northern China suggest links between subsistence changes and human migration. *Nat. Commun.* **11**, 2700 (2020).

65. M. Lipson, O. Cheronet, S. Mallick, N. Rohland, M. Oxenham, M. Pietrusewsky, T. O. Pryce, A. Willis, H. Matsumura, H. Buckley, K. Domett, N. G. Hai, T. H. Hiep, A. A. Kyaw, T. T. Win, B. Pradier, N. Broomandkhoshbacht, F. Candilio, P. Changmai, D. Fernandes, M. Ferry, B. Gamarra, E. Harney, J. Kampuansai, W. Kutanan, M. Michel, M. Novak, J. Oppenheimer, K. Sirak, K. Stewardson, Z. Zhang, P. Flegontov, R. Pinhasi, D. Reich, Ancient genomes document multiple waves of migration in Southeast Asian prehistory. *Science* **361**, 92–95 (2018).

66. M. A. Yang, X. Fan, B. Sun, C. Chen, J. Lang, Y.-C. Ko, C. Tsang, H. Chiu, T. Wang, Q. Bao, X. Wu, M. Hajdinjak, A. M.-S. Ko, M. Ding, P. Cao, R. Yang, F. Liu, B. Nickel, Q. Dai, X. Feng, L. Zhang, C. Sun, C. Ning, W. Zeng, Y. Zhao, M. Zhang, X. Gao, Y. Cui, D. Reich, M. Stoneking, Q. Fu, Ancient DNA indicates human population shifts and admixture in northern and southern China. *Science* **369**, 282–288 (2020).

67. G. M. Kılınç, A. Omrak, F. Özer, T. Günther, A. M. Büyükkarakaya, E. Bıçakçı, D. Baird, H. M. Dönertaş, A. Ghalichi, R. Yaka, D. Koptekin, S. C. Açan, P. Parvizi, M. Krzewińska, E. A. Daskalaki, E. Yüncü, N. D. Dağtaş, A. Fairbairn, J. Pearson, G. Mustafaoğlu, Y. S. Erdal, Y. G. Çakan, İ. Togan, M. Somel, J. Storå, M. Jakobsson, A. Götherström, The demographic development of the first farmers in Anatolia. *Curr. Biol.* **26**, 2659–2666 (2016).

68. E. R. Jones, G. Gonzalez-Fortes, S. Connell, V. Siska, A. Eriksson, R. Martiniano, R. L. McLaughlin, M. Gallego Llorente, L. M. Cassidy, C. Gamba, T. Meshveliani, O. Bar-Yosef, W. Müller, A. Belfer-Cohen, Z. Matskevich, N. Jakeli, T. F. G. Higham, M. Currat, D. Lordkipanidze, M. Hofreiter, A. Manica, R. Pinhasi, D. G. Bradley, Upper Palaeolithic genomes reveal deep roots of modern Eurasians. *Nat. Commun.* **6**, 8912 (2015).

69. C. Jeong, A. T. Ozga, D. B. Witonsky, H. Malmström, H. Edlund, C. A. Hofman, R. Hagan, M. Jakobsson, C. M. Lewis, M. Aldenderfer, A. Di Rienzo, C. Warinner, Long-term genetic stability and a high-altitude East Asian origin for the peoples of the high valleys of the Himalayan arc. *Proc. Natl. Acad. Sci. U.S.A.* **113**, 7485–7490 (2016).

70. M. Rasmussen, S. L. Anzick, M. R. Waters, P. Skoglund, M. DeGiorgio, T. W. Stafford Jr., S. Rasmussen, I. Moltke, A. Albrechtsen, S. M. Doyle, G. D. Poznik, V. Gudmundsdottir, R. Yadav, A.-S. Malaspinas, S. S. White V, M. E. Allentoft, O. E. Cornejo, K. Tambets, A. Eriksson, P. D. Heintzman, M. Karmin, T. S. Korneliussen, D. J. Meltzer, T. L. Pierre, J. Stenderup, L. Saag, V. M. Warmuth, M. C. Lopes, R. S. Malhi, S. Brunak, T. Sicheritz-Ponten, I. Barnes, M. Collins, L. Orlando, F. Balloux, A. Manica, R. Gupta, M. Metspalu, C. D. Bustamante, M. Jakobsson, R. Nielsen, E. Willerslev, The genome of a Late Pleistocene human from a Clovis burial site in western Montana. *Nature* **506**, 225–229 (2014).

71. A. Mittnik, C.-C. Wang, S. Pfrengle, M. Daubaras, G. Zariņa, F. Hallgren, R. Allmäe, V. Khartanovich, V. Moiseyev, M. Tõrv, A. Furtwängler, A. Andrades Valtueña, M. Feldman, C. Economou, M. Oinonen, A. Vasks, E. Balanovska, D. Reich, R. Jankauskas, W. Haak, S. Schiffels, J. Krause, The genetic prehistory of the Baltic Sea region. *Nat. Commun.* **9**, 442 (2018).

72. L. Saag, L. Varul, C. L. Scheib, J. Stenderup, M. E. Allentoft, L. Saag, L. Pagani, M. Reidla, K. Tambets, E. Metspalu, Extensive farming in Estonia started through a sex-biased migration from the Steppe. *Curr. Biol.* **27**, 2185–2193.e2186 (2017).

73.. Harney, H. May, D. Shalem, N. Rohland, S. Mallick, I. Lazaridis, R. Sarig, K. Stewardson, S. Nordenfelt, N. Patterson, I. Hershkovitz, D. Reich, Ancient DNA from Chalcolithic Israel reveals the role of population mixture in cultural transformation. *Nat. Commun.* **9**, 3336 (2018).

74. M. Rasmussen, M. Sikora, A. Albrechtsen, T. S. Korneliussen, J. V. Moreno-Mayar, G. D. Poznik, C. P. E. Zollikofer, M. S. Ponce de León, M. E. Allentoft, I. Moltke, H. Jónsson, C. Valdiosera, R. S. Malhi, L. Orlando, C. D. Bustamante, T. W. Stafford Jr., D. J. Meltzer, R. Nielsen, E. Willerslev, The ancestry and affiliations of Kennewick Man. *Nature* **523**, 455–458 (2015).

75. C. Jeong, S. Wilkin, T. Amgalantugs, A. S. Bouwman, W. T. T. Taylor, R. W. Hagan, S. Bromage, S. Tsolmon, C. Trachsel, J. Grossmann, J. Littleton, C. A. Makarewicz, J. Krigbaum, M. Burri, A. Scott, G. Davaasambuu, J. Wright, F. Irmer, E. Myagmar, N. Boivin, M. Robbeets, F. J. Rühli, J. Krause, B. Frohlich, J. Hendy, C. Warinner, Bronze Age

population dynamics and the rise of dairy pastoralism on the eastern Eurasian steppe. *Proc. Natl. Acad. Sci. U.S.A.* **115**, E11248–E11255 (2018).

76. M. Raghavan, M. Steinrücken, K. Harris, S. Schiffels, S. Rasmussen, M. DeGiorgio, A. Albrechtsen, C. Valdiosera, M. C. Ávila-Arcos, A.-S. Malaspinas, A. Eriksson, I. Moltke, M. Metspalu, J. R. Homburger, J. Wall, O. E. Cornejo, J. V. Moreno-Mayar, T. S. Korneliussen, T. Pierre, M. Rasmussen, P. F. Campos, P. d. B. Damgaard, M. E. Allentoft, J. Lindo, E. Metspalu, R. Rodríguez-Varela, J. Mansilla, C. Henrickson, A. Seguin-Orlando, H. Malmström, T. Stafford, S. S. Shringarpure, A. Moreno-Estrada, M. Karmin, K. Tambets, A. Bergström, Y. Xue, V. Warmuth, A. D. Friend, J. Singarayer, P. Valdes, F. Balloux, I. Leboreiro, J. L. Vera, H. Rangel-Villalobos, D. Pettener, D. Luiselli, L. G. Davis, E. Heyer, C. P. E. Zollikofer, M. S. P. de León, C. I. Smith, V. Grimes, K.-A. Pike, M. Deal, B. T. Fuller, B. Arriaza, V. Standen, M. F. Luz, F. Ricaut, N. Guidon, L. Osipova, M. I. Voevoda, O. L. Posukh, O. Balanovsky, M. Lavryashina, Y. Bogunov, E. Khusnutdinova, M. Gubina, E. Balanovska, S. Fedorova, S. Litvinov, B. Malyarchuk, M. Derenko, M. J. Mosher, D. Archer, J. Cybulski, B. Petzelt, J. Mitchell, R. Worl, P. J. Norman, P. Parham, B. M. Kemp, T. Kivisild, C. Tyler-Smith, M. S. Sandhu, M. Crawford, R. Villems, D. G. Smith, M. R. Waters, T. Goebel, J. R. Johnson, R. S. Malhi, M. Jakobsson, D. J. Meltzer, A. Manica, R. Durbin, C. D. Bustamante, Y. S. Song, R. Nielsen, E. Willerslev, Genomic evidence for the Pleistocene and recent population history of Native Americans. *Science* **349**, aab3884 (2015).

77. T. C. Lamnidis, K. Majander, C. Jeong, E. Salmela, A. Wessman, V. Moiseyev, V. Khartanovich, O. Balanovsky, M. Ongyerth, A. Weihmann, A. Sajantila, J. Kelso, S. Pääbo, P. Onkamo, W. Haak, J. Krause, S. Schiffels, Ancient Fennoscandian genomes reveal origin and spread of Siberian ancestry in Europe. *Nat. Commun.* **9**, 5018 (2018).

78. M. Rasmussen, Y. Li, S. Lindgreen, J. S. Pedersen, A. Albrechtsen, I. Moltke, M. Metspalu, E. Metspalu, T. Kivisild, R. Gupta, M. Bertalan, K. Nielsen, M. T. P. Gilbert, Y. Wang, M. Raghavan, P. F. Campos, H. M. Kamp, A. S. Wilson, A. Gledhill, S. Tridico, M. Bunce, E. D. Lorenzen, J. Binladen, X. Guo, J. Zhao, X. Zhang, H. Zhang, Z. Li, M. Chen, L. Orlando, K. Kristiansen, M. Bak, N. Tommerup, C. Bendixen, T. L. Pierre, B. Grønnow, M. Meldgaard, C. Andreasen, S. A. Fedorova, L. P. Osipova, T. F. G. Higham, C. B. Ramsey, T. O. Hansen, F. C. Nielsen, M. H. Crawford, S. Brunak, T. Sicheritz-Pontén, R. Villems, R. Nielsen, A.

Krogh, J. Wang, E. Willerslev, Ancient human genome sequence of an extinct Palaeo-Eskimo. *Nature* **463**, 757–762 (2010).

79. M. Krzewińska, G. M. Kılınç, A. Juras, D. Koptekin, M. Chyleński, A. G. Nikitin, N. Shcherbakov, I. Shuteleva, T. Leonova, L. Kraeva, F. A. Sungatov, A. N. Sultanova, I. Potekhina, S. Łukasik, M. Krenz-Niedbała, L. Dalén, V. Sinika, M. Jakobsson, J. Storå, A. Götherström, Ancient genomes suggest the eastern Pontic-Caspian steppe as the source of western Iron Age nomads. *Sci. Adv.* **4**, eaat4457 (2018).

80. M. Unterländer, F. Palstra, I. Lazaridis, A. Pilipenko, Z. Hofmanová, M. Groß, C. Sell, J. Blöcher, K. Kirsanow, N. Rohland, B. Rieger, E. Kaiser, W. Schier, D. Pozdniakov, A. Khokhlov, M. Georges, S. Wilde, A. Powell, E. Heyer, M. Currat, D. Reich, Z. Samashev, H. Parzinger, V. I. Molodin, J. Burger, Ancestry and demography and descendants of Iron Age nomads of the Eurasian Steppe. *Nat. Commun.* **8**, 14615 (2017).

81. M. A. Yang, X. Gao, C. Theunert, H. Tong, A. Aximu-Petri, B. Nickel, M. Slatkin, M. Meyer, S. Pääbo, J. Kelso, Q. Fu, 40,000-year-old individual from Asia provides insight into early population structure in Eurasia. *Curr. Biol.* **27**, 3202–3208.e9 (2017).

82. J. V. Moreno-Mayar, B. A. Potter, L. Vinner, M. Steinrücken, S. Rasmussen, J. Terhorst, J. A. Kamm, A. Albrechtsen, A.-S. Malaspinas, M. Sikora, J. D. Reuther, J. D. Irish, R. S. Malhi, L. Orlando, Y. S. Song, R. Nielsen, D. J. Meltzer, E. Willerslev, Terminal Pleistocene Alaskan genome reveals first founding population of Native Americans. *Nature* **553**, 203–207 (2018).

83. Q. Fu, H. Li, P. Moorjani, F. Jay, S. M. Slepchenko, A. A. Bondarev, P. L. F. Johnson, A. Aximu-Petri, K. Prüfer, C. de Filippo, M. Meyer, N. Zwyns, D. C. Salazar-García, Y. V. Kuzmin, S. G. Keates, P. A. Kosintsev, D. I. Razhev, M. P. Richards, N. V. Peristov, M. Lachmann, K. Douka, T. F. G. Higham, M. Slatkin, J.-J. Hublin, D. Reich, J. Kelso, T. B. Viola, S. Pääbo, Genome sequence of a 45,000-year-old modern human from western Siberia. *Nature* **514**, 445–449 (2014).

84. I. Lazaridis, N. Patterson, A. Mittnik, G. Renaud, S. Mallick, K. Kirsanow, P. H. Sudmant, J. G. Schraiber, S. Castellano, M. Lipson, B. Berger, C. Economou, R. Bollongino, Q. Fu, K. I. Bos, S. Nordenfelt, H. Li, C. de Filippo, K. Prufer, S. Sawyer, C. Posth, W. Haak, F.

Hallgren, E. Fornander, N. Rohland, D. Delsate, M. Francken, J.-M. Guinet, J. Wahl, G. Ayodo, H. A. Babiker, G. Bailliet, E. Balanovska, O. Balanovsky, R. Barrantes, G. Bedoya, H. Ben-Ami, J. Bene, F. Berrada, C. M. Bravi, F. Brisighelli, G. B. J. Busby, F. Cali, M. Churnosov, D. E. C. Cole, D. Corach, L. Damba, G. van Driem, S. Dryomov, J.-M. Dugoujon, S. A. Fedorova, I. Gallego Romero, M. Gubina, M. Hammer, B. M. Henn, T. Hervig, U. Hodoglugil, A. R. Jha, S. Karachanak-Yankova, R. Khusainova, E. Khusnutdinova, R. Kittles, T. Kivisild, W. Klitz, V. Kucinskas, A. Kushniarevich, L. Laredj, S. Litvinov, T. Loukidis, R. W. Mahley, B. Melegh, E. Metspalu, J. Molina, J. Mountain, K. Nakkalajarvi, D. Nesheva, T. Nyambo, L. Osipova, J. Parik, F. Platonov, O. Posukh, V. Romano, F. Rothhammer, I. Rudan, R. Ruizbakiev, H. Sahakyan, A. Sajantila, A. Salas, E. B. Starikovskaya, A. Tarekegn, D. Toncheva, S. Turdikulova, I. Uktveryte, O. Utevska, R. Vasquez, M. Villena, M. Voevoda, C. A. Winkler, L. Yepiskoposyan, P. Zalloua, T. Zemunik, A. Cooper, C. Capelli, M. G. Thomas, A. Ruiz-Linares, S. A. Tishkoff, L. Singh, K. Thangaraj, R. Villems, D. Comas, R. Sukernik, M. Metspalu, M. Meyer, E. E. Eichler, J. Burger, M. Slatkin, S. Paabo, J. Kelso, D. Reich, J. Krause, Ancient human genomes suggest three ancestral populations for present-day Europeans. *Nature* **513**, 409–413 (2014).

85. S. Mallick, H. Li, M. Lipson, I. Mathieson, M. Gymrek, F. Racimo, M. Zhao, N. Chennagiri, S. Nordenfelt, A. Tandon, P. Skoglund, I. Lazaridis, S. Sankararaman, Q. Fu, N. Rohland, G. Renaud, Y. Erlich, T. Willems, C. Gallo, J. P. Spence, Y. S. Song, G. Poletti, F. Balloux, G. van Driem, P. de Knijff, I. G. Romero, A. R. Jha, D. M. Behar, C. M. Bravi, C. Capelli, T. Hervig, A. Moreno-Estrada, O. L. Posukh, E. Balanovska, O. Balanovsky, S. Karachanak-Yankova, H. Sahakyan, D. Toncheva, L. Yepiskoposyan, C. Tyler-Smith, Y. Xue, M. S. Abdullah, A. Ruiz-Linares, C. M. Beall, A. Di Rienzo, C. Jeong, E. B. Starikovskaya, E. Metspalu, J. Parik, R. Villems, B. M. Henn, U. Hodoglugil, R. Mahley, A. Sajantila, G. Stamatoyannopoulos, J. T. S. Wee, R. Khusainova, E. Khusnutdinova, S. Litvinov, G. Ayodo, D. Comas, M. F. Hammer, T. Kivisild, W. Klitz, C. A. Winkler, D. Labuda, M. Bamshad, L. B. Jorde, S. A. Tishkoff, W. S. Watkins, M. Metspalu, S. Dryomov, R. Sukernik, L. Singh, K. Thangaraj, S. Pääbo, J. Kelso, N. Patterson, D. Reich, The simons genome diversity project: 300 genomes from 142 diverse populations. *Nature* **538**, 201–206 (2016).

86. S. Rubinacci, D. M. Ribeiro, R. J. Hofmeister, O. Delaneau, Efficient phasing and imputation of low-coverage sequencing data using large reference panels. *Nat. Genet.* **53**, 120–126 (2021).

87. P. Danecek, J. K. Bonfield, J. Liddle, J. Marshall, V. Ohan, M. O. Pollard, A. Whitwham, T. Keane, S. A. McCarthy, R. M. Davies, H. Li, Twelve years of SAMtools and BCFtools. *GigaScience* **10**, giab008 (2021).

88. H. Jónsson, A. Ginolhac, M. Schubert, P. L. Johnson, L. Orlando, mapDamage2.0: Fast approximate Bayesian estimates of ancient DNA damage parameters. *Bioinformatics* **29**, 1682–1684 (2013).

89. G. Renaud, V. Slon, A. T. Duggan, J. Kelso, Schmutzi: Estimation of contamination and endogenous mitochondrial consensus calling for ancient DNA. *Genome Biol.* **16**, 224 (2015).

90. T. S. Korneliussen, A. Albrechtsen, R. Nielsen, ANGSD: Analysis of next generation sequencing data. *BMC Bioinformatics* **15**, 356 (2014).

91. M. Caballero, D. N. Seidman, Y. Qiao, J. Sannerud, T. D. Dyer, D. M. Lehman, J. E. Curran, R. Duggirala, J. Blangero, S. Carmi, A. L. Williams, Crossover interference and sex-specific genetic maps shape identical by descent sharing in close relatives. *PLOS Genet.* **15**, e1007979 (2019).

92. C. Bhérer, C. L. Campbell, A. Auton, Refined genetic maps reveal sexual dimorphism in human meiotic recombination at multiple scales. *Nat. Commun.* **8**, 14994 (2017).

93. C. L. Campbell, N. A. Furlotte, N. Eriksson, D. Hinds, A. Auton, Escape from crossover interference increases with maternal age. *Nat. Commun.* **6**, 6260 (2015).

94. H. Ringbauer, J. Novembre, M. Steinrücken, Parental relatedness through time revealed by runs of homozygosity in ancient DNA. *Nat. Commun.* **12**, 5425 (2021).

95. H. Weissensteiner, D. Pacher, A. Kloss-Brandstätter, L. Forer, G. Specht, H.-J. Bandelt, F. Kronenberg, A. Salas, S. Schönherr, HaploGrep 2: Mitochondrial haplogroup classification in the era of high-throughput sequencing. *Nucleic Acids Res.* **44**, W58–W63 (2016).

96. G. D. Poznik, Identifying Y-chromosome haplogroups in arbitrarily large samples of sequenced or genotyped men. *bioRxiv* **088716**, (2016).

97. R. Maier, P. Flegontov, O. Flegontova, U. Işıldak, P. Changmai, D. Reich, On the limits of fitting complex models of population history to f-statistics. *eLife* **12**, e85492 (2023).

98. M. Chintalapati, N. Patterson, P. Moorjani, The spatiotemporal patterns of major human admixture events during the European Holocene. *eLife* **11**, e77625 (2022).

99. C. Bronk Ramsey, Bayesian analysis of radiocarbon dates. *Radiocarbon* **51**, 337–360 (2009).

100. E. A. Bullion, *Trans-Eurasian Exchanges: Contemporary Dialogues and Archaeological Enquiry, Final Osteological Report* (Nazarbayev University's Anthropology Laboratory Archive, 2020).

101. L. Lacher, *Report on the Burial Ground Koken* (Nazarbayev University's Anthropology Laboratory Archive, 2022).

102. I. A. Kukushkin, E. A. Dimitriev, A. I. Kukushkin, Fedorov burials of the Tallinsky-1 burial ground (ФЕДОРОВСКИЕ ЗАХОРОНЕНИЯ МОГИЛЬНИКА ТАЛЛИНСКИЙ-1). *Preservation Study Cultural Heritage Altai Territory* **26**, 76–83 (2020).

103. V. V. Varfolomeev, E. A. Dmitriev, V. G. Loman, Burial Korzhar—New Bronze Age monument of central Kazakhstan. *History Archaeology Semirechye (История и археология Семиречья)* **6**, 50–74 (2019).

104. A. Tishkin, Y. V. Frolov, Bronze Age axes from the forest-steppe Altai. *Archaeology Ethnology Anthropology Eurasia* **45**, 87–96 (2017).
